# Phosphodiesterase 7: a potential novel therapeutic target in ovarian cancer

**DOI:** 10.3389/fphar.2025.1566330

**Published:** 2025-06-04

**Authors:** Nayara Gusmão Tessarollo, Isabella dos Santos Guimarães, Diandra Zipinotti dos Santos, Taciane Barbosa Henriques, Paulo Cilas Morais Lyra-Junior, Josiany Carlos de Souza, Tatiana Massariol Pimenta, Bárbara da Silva Martins, Solenny Maria Silva Butzene, José Matheus Simões Padilha, Leide Laura Figueiredo Maciel, João Carlos de Aquino Almeida, Ian Victor Silva, Leticia Batista Azevedo Rangel

**Affiliations:** ^1^ Biotechnology Program/RENORBIO, Health Sciences Center, Federal University of Espírito Santo, Vitória, Espírito Santo, Brazil; ^2^ Division of Clinical Research and Technological Development, Brazilian National Cancer Institute (INCA), Rio de Janeiro, Brazil; ^3^ Department of Pharmaceutical Sciences, Health Sciences Center, Federal University of Espírito Santo, Vitória, Espírito Santo, Brazil; ^4^ Laboratory of Physiology and Biochemistry of Microorganisms, State University of North Fluminense Darcy Ribeiro, Campos, Brazil; ^5^ Department of Morphology, Health Sciences Center, Federal University of Espírito Santo, Vitória, Espírito Santo, Brazil; ^6^ Biochemistry Program, Health Sciences Center, Federal University of Espírito Santo, Vitória, Espírito Santo, Brazil

**Keywords:** ovarian cancer, phosphodiesterase 7-A, PI3K/AKT/mTOR signaling pathway modulation, paclitaxel, mitochondrial cristae morphology alteration

## Abstract

**Introduction:**

Chemoresistance and disease relapses in epithelial ovarian cancer (EOC) highlight the need for novel therapeutic strategies. Here, we investigated phosphodiesterase 7A (PDE7A) as a potential target in ovarian cancer treatment.

**Methods:**

Gene expression was performed by RNA sequencing data comparing high-grade serous ovarian carcinoma (HGSOC) and fallopian tube samples. The PDE7 inhibitor BRL 50481, alone or combined with paclitaxel (PTX), was tested in drug-sensitive A2780 and multi-resistant OVCAR3 cells by Diphenyltetrazolium bromide (MTT) assay. To validate data from the high throughput RNA-sequencing assays, RT-qPCR and Immunoblotting were performed. Cytokine expression was analyzed by RT-qPCR and the quantification was obtained by ELISA. Scanning and Transmission Electron Microscopy were also carried out.

**Results and discussion:**

MTT assays revealed that while BRL 50481 reduced metabolic cellular viability (MCV) in A2780 (IC50 = 200 μM), its combination with PTX decreased MCV in both lines, reducing PTX IC50 by 103- and 625-fold in A2780 and OVCAR3, respectively. PDE7 inhibition suppressed the PI3K/AKT/mTOR pathway, upregulated the pro-apoptotic protein Bcl-2 Associated X-protein (BAX) in A2780, and increased IL-6 expression in OVCAR3. Pretreatment with BRL 50481 followed by PTX downregulated vimentin and octamer-binding transcription factor (OCT4), while inducing morphological changes and mitochondrial cristae alterations. Inhibiting PDE7 can enhance the paclitaxel-induced apoptosis by promoting mitochondrial dysfunction and suppressing survival pathways, thereby improving ovarian cancer treatment efficacy. The results need to be validated in additional in vivo models.

## 1 Introduction

Ovarian cancer (OC) is the leading cause of deaths by gynecological tumors (Lele, 2022). Data from the American Cancer Society ranked OC as the fifth cause of cancer-related deaths amongst women; 20,890 new cases and 12,730 deaths caused by the disease being estimated in the United States in 2025 (American Cancer Society, 2025). Epithelial ovarian cancer (EOC) is not a homogeneous pathology but rather a group of diseases with morphological and etiological differences that share the same anatomical site. Molecular and pathological findings have shown that tumors can derive from non-ovarian tissues and different histological subtypes share molecular similarities (Prat and FIGO Committee on Gynecologic Oncology, 2014; Vaughan et al., 2011).

In this context, Kurman and Shih proposed, in 2016, a dualistic model comprising two major groups of EOC: i) type I tumors, which include low-grade serous, endometrioid, clear cell and mucinous carcinomas, malignant Brenner tumor and seromucinous carcinoma; ii) type II tumors that encompass high-grade serous ovarian carcinoma (HGSOC), carcinosarcoma and undifferentiated carcinoma (Kurman and Shih, 2016). Types I and II tumors have distinct genetics, molecular characteristics, morphological patterns, and etiology. Robust evidence shows that HGSOC arises from a non-invasive occult carcinoma in the distal fallopian tube, designated serous tubal intraepithelial carcinoma (STIC) (Kurman and Shih, 2011). STICs have been identified in 10%–15% specimens obtained from women carrying germline BRCA mutations (Chui et al., 2009; Colgan et al., 2001). The initial response of HGSOC to the first-line therapeutic approach based on platinum and taxane derivatives is greater than 80% (Hennessy et al., 2009). However, the majority of patients have tumor recurrence, so that the mean disease-free survival rate is lower than 18 months following its diagnosis (Modugno and Edwards, 2012). Due to the high rate of acquisition of the chemoresistant phenotype by cancer cells, researchers and clinicians are dedicating their efforts to identify new strategies to fight EOC through novel cellular targets, drug discovery and different drug combinations.

Paclitaxel, a naturally occurring hydrophobic compound, belongs to the taxane family of anticancer drugs and is widely used as a potent cytotoxic agent to treat different cancer types, including breast, ovarian, lung, esophageal, gastric, pancreatic cancer, and neck cancer cells (Hassan et al., 2024; Nawara et al., 2021; Boyd and Muggia, 2018). Its primary mechanism of action involves targeting β-tubulin, leading to microtubule stabilization, cell cycle arrest and apoptosis (Alqahtani et al., 2019). In addition, paclitaxel has been found to target the mitochondria and inhibit the function of the apoptosis inhibitor protein B cell Leukemia 2 (Bcl-2) (Ferlini et al., 2003). While cancer cells are initially susceptible to paclitaxel, resistance often develops through both intrinsic and acquired mechanisms. These include DNA mutations that alter metabolic pathways involved in drug resistance and degradation, activation of drug efflux, modifications in apoptotic signaling pathways, and upregulation of paclitaxel resistance-associated gene-3 (TRAG-3/CSAG2) expression (Alatise et al., 2022).

High throughput RNA sequencing assays were run aiming the comparison of transcriptomes from fresh samples of HGSOC and fallopian tube, from which 243 differentially expressed transcripts were identified. Amongst them, PDE7A was one of the twenty highly overexpressed transcripts in HGSOC in comparison to fallopian tube cells (2.54 times; p = 0.0001).

PDE enzymes are formed by eleven structurally related gene superfamilies (PDE1 to PDE11) that differ in their primary structures, cellular functions, affinities for the nucleotides cAMP and cGMP, catalytic properties and regulatory mechanisms (Francis et al., 2011; Keravis and Lugnier, 2012; Kelly et al., 2025). PDE7, including isoforms *PDE7A* and *PDE7B*, is a high-affinity cAMP-specific PDE. Three variants of splicing are described in humans, *PDE7A1, PDE7A2* and *PDE7A3*, which differ from each other in N and C-terminal regions (Han et al., 1997). The distribution of PDE isoenzymes in the ovary differs according to their family, with *PDE7A* and *PDE7B* are found in oocytes (Petersen et al., 2015).

Several series of PDE7 inhibitors have been developed, belonging to different chemical groups, including thiadiazole derivatives (Redondo et al., 2012), sulfonamide derivatives (Smith et al., 2004), thioxoquinazoline derivatives (Castaño et al., 2009) and pyrimidine-based inhibitors (Guo et al., 2009), among others. These inhibitors differ in their affinity for PDE family members. For example, BMS-586353 exhibits selectivity against multiple PDE isoenzymes, including PDE1 and PDE3-6 (Yang et al., 2003), while 8-bromo-9-substituted guanine derivatives act as selective PDE7A inhibitors with minimal activity against PDE3 and PDE4 (Barnes et al., 2001). The discovery of BRL 50481 (N, N, 2-Trimethyl-5-nitro-benzenesulfonamide), a sulfonamide-based selective PDE7 inhibitor, represented a significant advancement in targeting alternative cAMP phosphodiesterases to alleviate chronic inflammation in chronic obstructive pulmonary disease (COPD) (Smith et al., 2004). BRL 50481 showed to selectively inhibit PDE7A1 (IC_50_ = 0.26 and 2.4 µM, at 0.05 µM and 1 µM of cAMP, respectively), exhibiting a 200-fold selectivity over other PDE isoenzymes. In addition, kinetic analysis revealed that BRL 50481 acts as a competitive inhibitor of hrPDE7A1 expressed in baculovirus-infected *Spodoptera frugiperda 9* cells (Ki ∼180 nM). Also, the compound was found to be significantly less potent against other PDE isoenzymes (Smith et al., 2004).

Inhibition of PDEs isoforms has been proven efficient in treating diseases such as erectile dysfunction, asthma, obstructive pulmonary chronic disease, and hematologic cancer (Ahmad et al., 2015; Yamamoto et al., 2015; Lin et al., 2013; Hirsh et al., 2004). PDE4 inhibitors often lead to side effects, such as nausea and vomiting, which limit their clinical utility (Schick and Schlegel, 2022). In contrast, PDE7 inhibitors are considered a good strategy to mitigate these adverse effects through a more subtle modulation of cAMP levels (Giembycz, 2005) while avoiding emetogenic activity. This was demonstrated by García et al. (2014) who compared the emetogenic effects of the PDE7 inhibitor BRL 50481 (5 mg/kg) with those of the PDE4 inhibitors rolipram (1 mg/kg) and roflumilast (1 mg/kg). Despite using a five-fold higher dose, BRL 50481 did not induce any side effects in a mouse model (García et al., 2014). The same was found by Świerczek et al. (2021) using GRMS-55, a potent PDE7A inhibitor, which led to a high hepatoprotective activity in mice with autoimmune hepatitis without inducing an emetic effect (Świerczek et al., 2021). Although the PDE7 inhibition appears to offer a more favorable side effect profile, further studies are needed to better clarify this issue.

Yamamoto et al. (2015) demonstrated that PDE7A expression is increased in samples of endometrioid carcinoma. Silencing this protein significantly inhibited cell migration and invasion. Similarly, in triple-negative breast cancer cell lines MDA-MB231 and Hs578T, PDE7A has been shown to promote tumor growth (Zhang et al., 2019). Nonetheless, the role of PDE7 in ovarian tumors remains unclear.

Beyond its role in cAMP degradation, the regulatory domain of the PDE7A1 splice variant contains a tandemly repeated PKA pseudosubstrate motif. This suggests that, similar to PKI and PKA regulatory subunits, PDE7A1 may regulate cAMP signaling by binding to and inhibiting the catalytic subunit C (Han et al., 2006). In ovarian cancer, growth and metabolism could be driven by alterations in the cAMP-PKA-CREB signaling axis (Kilanowska et al., 2022). Özeş et al. (2018) demonstrated that PKA can phosphorylate EZH2, leading to mitochondrial dysfunction and the interaction of EZH2 with STAT3, ultimately inhibiting STAT3 phosphorylation and suppressing epithelial ovarian cancer cell growth (Özeş et al., 2018).

Given the role of PDE7 in cAMP degradation and its regulatory interaction with PKA, targeting PDE7 may increase the cAMP-PKA signaling, enhancing the paclitaxel-induced apoptosis via mitochondrial dysfunction and suppression of survival pathways, thus improving the effectiveness in ovarian cancer.

## 2 Materials and methods

### 2.1 Cell lines and cell culture

A2780 (Sigma-Aldrich, German), ovarian endometrioid adenocarcinoma tumor cells provided from untreated patient and not mutant to p53, and OVCAR3, high grade serous ovarian cancer cell line provided from malignant ascites of a patient with progressive ovarian adenocarcinoma and resistant to cisplatin, melphalan, and adriamycin (Cell Bank of the Federal University of Rio de Janeiro, Brazil) were cultured in RPMI-1640 medium (Gibco) supplemented with 10% (v/v) fetal bovine serum (FBS) (Gibco), 0.2% (w/v) sodium bicarbonate, 1% (v/v) stabilized Penicillin (100 units/mL) and Streptomycin (100 μg/mL) solution (Gibco, United States), 1% (v/v) Amphotericin B (Sigma-Aldrich, Germany) at 37°C in a humidified atmosphere of 5% CO2.

STR documents from OVCAR5, OVCAR8, OVCAR429, and SKOV3 are attached to the Supplementary Material of the manuscript (Supplementary Figure S1). The other cell lines were genotyped earlier.

### 2.2 Validation of RNA-Sequencing by RT-qPCR and immunoblotting

To validate data from the high-throughput RNA sequencing assays (data kindly provided by Dr. Shih from the Johns Hopkins University), RT-qPCR experiments were conducted for the nine higher and lower expressed transcripts in the same samples of HGSOC and fallopian tube. Samples are pre-de-identified legacy cases. This study is classified as exempt under category 4 and is considered non-human subject research (HSR). In most cases we handle, HSRs are clinical trial research studies. Approximately 8 years ago, the Collaborative Institute at Johns Hopkins started to consent all patients donating their tissues for research. Nonetheless, cases in the present study are legacy samples collected before the referred implementation.

The gene expression level, p-value and q-value from RNA sequencing data are presented in Supplementary Table S1. The sequences of the primers are available in Supplementary Table S2. Focusing on our molecule of interest, PDE7 expression was further screened by RT-qPCR in EOC cell lines (OVCAR3, OVCAR4, OVCAR5, OVCAR8, OVCAR249, SKOV3, EOF21, TKMN, CaOV3, MPSC1, ES-2, and OVTOKO), fallopian tube cell line FT2821 and in four lines derived from the normal ovary surface (OSE4, OSE7, OSE10, and IOSE80). Briefly, RNA samples were extracted using the RNeasy kit Mini Kit (Qiagen, Germany) according to the manufacturer’s protocol, and the concentration of RNA was determined by Nanodrop^®^ Spectrophotometer ND-1000 (Nanodrop^®^, United States). cDNA was synthesized by iScript cDNA Synthesis Kit (Bio-Rad^®^, United States), according to the manufacturer. RT-qPCR was performed on CFX96 equipment (Bio-Rad^®^, United States) with the SYBR Green I detection system (Invitrogen, United States). Quantification of relative gene expression was performed by the 2^−ΔΔCT^ method using GAPDH expression for normalization and experimental internal control.

PDE7 protein expression was also evaluated by immunoblotting using the polyclonal antibody HPA027340 (anti-PDE7A, 1:10; Sigma-Aldrich, Germany). AKT (anti-AKT, 1:250; Cell Signaling Technology, United States), Phospho-AKT (anti-Phospho-AKT, 1:250; Cell Signaling Technology, United States), BAX (anti-BAX, 1:500; BD Biosciences, EUA) and GAPDH (anti-GAPDH; 1:5,000, Cell Signaling Technology, United States) protein expression were also evaluated by immunoblotting. In brief, total cell extracts were obtained with RIPA lysis buffer (50 mM Tris-HCl pH 7.4, 150 mM NaCl, 1% (v/v) Triton 100x, 0.5% (w/v) sodium deoxycholate, 0.1% (w/v) SDS) plus phosphatase and protease inhibitors (Sigma-Aldrich, Germany) (Cao et al., 2005). Protein concentration was quantified by Bradford assay (Sigma-Aldrich, Germany) with bovine serum albumin as the standard protein, according to the manufacturer’s protocol. Thirty μg of total protein were applied to 10% (w/v) SDS polyacrylamide gel and separated by electrophoresis under reducing conditions (Laemmli, 1970). Proteins were transferred to nitrocellulose membranes (Amersham Biosciences, United Kingdom). Non-specific binding was blocked with 5% (w/v) skim milk in Tris-Buffer Saline (TBS) containing 0.05% (v/v) Tween 20 for 1 h at room temperature. Following the incubation with the primary antibody, samples were incubated with horseradish peroxidase (HRP)-conjugated anti-rabbit IgG (Pierce Biotechnology, United States) secondary antibody (1:5,000) diluted in TBS containing 0.1% (v/v) Tween 20% and 0.5% (w/v) skim milk. A chemiluminescence detection kit (Amersham Biosciences, United Kingdom) was used to develop the film following the manufacturer’s protocol. Bands densitometry was quantified using Image Lab 5.1 Software (Bio-Rad^®^, United States) and normalized by GAPDH. Blots were run as n ≥ 3 independent experiments.

### 2.3 In silico molecular docking to assess BRL50481 binding to PDE7

The BRL50481 (N,N,2-trimethyl-5-nitrobenzenesulfonamide) 3D conformer was obtained from the PubChem database (National Center for Biotechnology Information, 2025a). The molecule was optimized using WebMO (MOPAC) (Polik and Schmidt, 2021). Then, the structure was converted to Sybyl Mol2 format using Discovery Studio 2024 (BIOVIA and Dassault Systèmes, 2025). Additionally, the crystallized PDE7A protein (PDB ID: 1ZKL) (Wang et al., 2005) was collected from the Protein Data Bank (PDB) to examine the coordinates of the protein’s active site through the already crystallized ligand in complex with it, using the Discovery Studio platform. Afterward, the AutoDock Vina (SwissDock) (Trott and Olson, 2010; Eberhardt et al., 2021) software was employed to perform the BRL 50481 docking with the protein. The validation method was carried out through re-docking of the already crystallized ligand (3-isobutyl-1-methylxanthine) (National Center for Biotechnology Information, 2025b) using the same method. Re-docking was performed using the DockRMSD software (Bell and Zhang, 2019), a docking program designed to re-fit a compound with known conformation and orientation into the active site of the target (Bell and Zhang, 2019). It is important to note that positions with a root-mean-square deviation (RMSD) below a preselected threshold (typically 2 Å) from the known conformation are considered successfully reproduced, indicating that the ligand position closely resembles the experimental state (Hevener et al., 2009).

### 2.4 MTT assays

The cytotoxic effect of paclitaxel (PTX) (Acoord Farmaceutica) and the PDE7 inhibitor BRL 50481 (Tocris Bioscience, United Kingdom) was investigated by 3-(4,5-dimethylthiazol-2-yl)-2,5-diphenyltetrazolium bromide (MTT) assay (Sigma-Aldrich, Germany) (Riss et al., 2013). Briefly, cells were seeded (2 × 10^4^ to 1 × 10^5^ cells/well) on 96-well plates and allowed to adhere before treatment with PTX and BRL 50481, for 24 and 48 h. The concentration range of drugs for each cell line is provided in the figure legend. Drug-free medium was added to cells for experimental control. Plates were incubated with 0.5 mg/mL MTT solution for 4 h in the dark. Tetrazolium crystals were dissolved with DMSO. MTT absorbance was acquired at 540 nm in a microplate reader Bio-Rad^®^, United States). For the IC_50_ calculation, GraphPad Prism software version 8.0 was used.

### 2.5 Flow cytometry analysis

A2780 and OVCAR3 cell lines were seeded at 0.8x10^6^ cells/mL in six-well plates and treated with BRL 50481 and PTX as monotherapies and combined therapies. The concentration of drugs for each cell line is provided in the figure legend. After 24 h, both supernatant and the cells were collected and resuspended in a binding buffer (10 mM HEPES, 150 mM NaCl, 5 mM KCl, 1 mM MgCl_2_, and 1.8 mM CaCl_2_). Samples were then incubated with Annexin V-FITC and subsequently stained with propidium iodide (PI) for flow cytometric analysis, using a BD Accuri^®^ C6 Flow Cytometer. A total of 10,000 events per sample were analyzed, assessing apoptotic and necrotic cell populations with Summit v.4.3 software.

### 2.6 Gene expression analysis by RT-qPCR

Cells from the A2780 and OVCAR3 were seeded in a 6-well plate (0.8 × 10^6^ cells/mL) and, after 24 h of adhesion, treated with BRL 50481 and PTX as monotherapies and combined therapies. Briefly, A2780 cells were treated with 200 μM BRL 50481, 1 × 10^−7^ nM PTX and pre-treated with 200 μM BRL 50481 following the 200 BRL 504881 μM + PTX 1 x 10^−7^ nM; OVCAR3 cells received 400 μM BRL 50481, 5 × 10^−4^ nM PTX and pre-treated with 400 μM BRL 50481 following the 400 μM BRL 50481 + PTX 5 × 10^−4^ nM. Gene expression level was compared to the control (DMSO-treated condition). After 24 h of treatment, the culture medium was collected for cytokine analysis, and total RNA was extracted using Trizol (Invitrogen, United States), following the manufacturer’s protocol. Gene expression of Interleukin-6 (IL-6), IL-1α, IL-1β, vimentin, OCT4 and Nanog was evaluated by RT-qPCR. Quantification of relative gene expression was performed by the 2^−ΔΔCT^ method using GAPDH expression for normalization and experimental internal control. Sequences of the primers are listed in Supplementary Table S1.

### 2.7 Dosage of IL-6 by ELISA

Interleukin-6 secreted in A2780 and OVCAR3 conditioned culture medium was quantified by ELISA (PeproTech, United States), according to the manufacturer’s instructions. The cytokine concentration was calculated using the linear regression derived from the standard curve.

### 2.8 Scanning electron microscopy

A2780 and OVCAR3 cells were seeded at 4 × 10^5^ cells/mL density on 12-well plates under sterile coverslips for cell growth; then treated with PTX (0.53 μM and 12.5 μM, respectively) and BRL 50481 (200 μM and 400 μM, respectively) in monotherapy or under combined conditions. Then, cells were washed twice with PBS 1x pH 7.2 (Sigma-Aldrich, Germany) at room temperature. Thereafter, the material was fixed on the coverslips with 2.5% (w/v) glutaraldehyde and 2% (w/v) formaldehyde (Sigma-Aldrich, Germany) in the cacodylate buffer 0.1 M pH 7.2. Fixed cells were washed twice for 5 min each with PBS 1x pH 7.2 (Sigma-Aldrich, Germany), post-fixed with 1% (w/v) osmium tetroxide (Sigma-Aldrich, Germany) and 0.8% (w/v) potassium ferricyanide (Sigma-Aldrich, Germany), washed three times with the same buffer and dehydrated for 20 min each in increasing series of ethanol concentrations: 50% (v/v), 70% (v/v), 90% (v/v) and 100% (v/v). Samples were dried in a CPD 030 Critical Point Dryer (BAL-TEC), sprayed with palladium (metallization) in the Sputter Coater SDC 050 and observed in an electron microscope scanning (ZEISS EVO 40 XVP) at 15 KV.

### 2.9 Ultrastructural analysis of cells organelles by transmission electron microscopy

A2780 and OVCAR3 cells were seeded at 4 × 10^5^ cells/mL on 12-wells plate, at 37°C in a humidified atmosphere of 5% CO2; then treated with PTX (0.53 μM and 12.5 μM, respectively) and BRL 50481 (200 μM and 400 μM, respectively) in monotherapy or under combined condition. Cells were washed with PBS 1X pH 7.2 (Sigma-Aldrich, Germany) at room temperature. Cells were trypsinized and centrifuged at 300 *g* for 3 min, the pellet being washed twice with PBS 1X. The material was fixed for 2 h at room temperature using 2.5% (w/v) glutaraldehyde (Sigma-Aldrich, Germany), 2% (w/v) formaldehyde (Sigma-Aldrich, Germany) and cacodylate buffer 0.1M pH 7.2 and post-fixed for 20 min in a 1:1 1% (w/v) solution of osmium tetroxide (Sigma-Aldrich, Germany) and 0.8% (w/v) potassium ferrocyanide (Sigma-Aldrich, Germany). Then, cells were dehydrated sequentially for 20 minutes in each step in a graduated series of acetone concentrations: 50% (v/v), 70% (v/v), 90% (v/v) and 100% (v/v) for later incorporation in epoxy resin (Epon 812^®^, Sigma-Aldrich, Germany). Cells embedded in resin were placed in a silicone mold at 60°C for 48 h for polymerization. Blocks were sectioned in ultrafine sections (70 nm), stained with uranyl acetate and lead citrate, and observed in transmission electron microscope (JEOL-1400 Plus) at 120 KV.

### 2.10 Statistical analysis

All results are presented as mean ± standard deviation (SD) of at least 3 independent experiments. Statistical analyses were performed using GraphPad Prism version 8.0 for Windows (GraphPad Software). p < 0.05 was considered statistically significant. p-values are indicated in the legends. Statistical tests and post-tests used for each experiment are cited in the figures’ legends.

## 3 Results

### 3.1 Validation of PDE7A expression in HGSOC by RT-qPCR and Western blot

**FIGURE 1:**
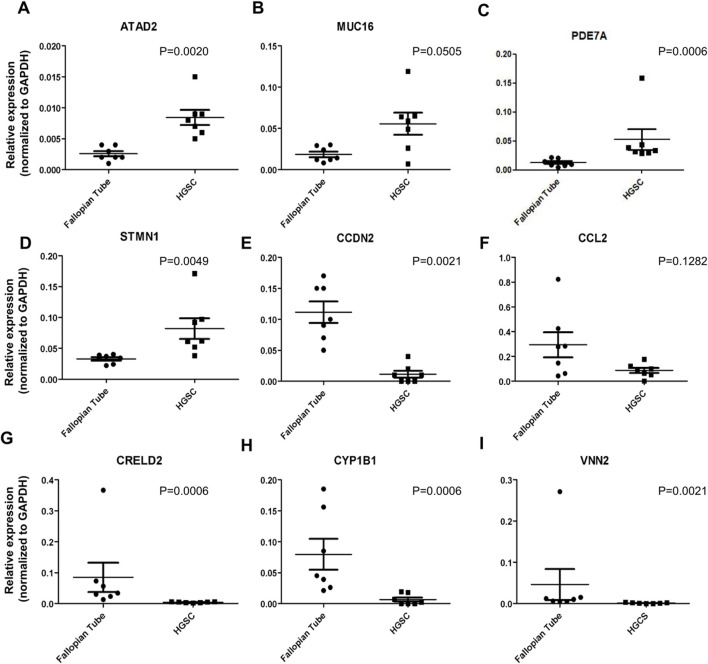
Validation of gene expression by RT-qPCR. **(A-I)** The relative expression of genes in seven fallopian tube samples and seven samples from HGSC patients, as determined by RNA sequencing data, was validated using RT-qPCR.

Amongst the first twenty genes from the high-throughput RNA sequencing data (data gently provided by Dr. Shih from Johns Hopkins University), we designed primers for nine of them. The log2 fold change, p-value and results were validated by RT-qPCR in the same samples used for the sequencing experiments aforementioned: ATAD2, MUC16, PDE7A, STMN1 genes were found upregulated (p < 0.05; except MUC16) in HGSOC samples, while CCND2, CRELD2, CYP1B1, CCL2 and VNN2 (p < 0.05; except CCL2) were downregulated compared to fallopian tube samples. Analysis of gene expression of nine targets listed above is demonstrated in Figure 1. We highlight the relative expression of the *PDE7A* gene was 0.052 in HGSOC samples, compared to 0.012 in fallopian tube samples (p-value = 0.006) (Figure 1C).

We also confirmed the highest expression of target PDE7A in HGSOC cell lines compared to the fallopian tube and normal ovarian cells by RT-qPCR (Supplementary Figure S2A). RT-qPCR analysis showed that *PDE7A* gene was 2.3 and 2.8 more expressed in OVCAR3 and OVCAR4 cell lines, respectively, compared to the fallopian tube cell FT2821 (p < 0.05). We observed a similar profile in protein expression detected by Western blot (Supplementary Figure S2B). PDE7A level was higher in OVCAR3 cell line, a HGSOC multi-resistance lineage, than in FT2821 and OSE4, OSE7, and OSE10, normal Fallopian tube and normal ovarian epithelial cells, respectively. These results are consistent with the analysis from The Human Protein Atlas (Supplementary Figure S2C), which showed that the PDE7A exhibits one of the highest expression levels among ovarian cancer cell lines and is higher compared to other cell lines classified as HGSOC.

### 3.2 Molecular docking analysis of BRL50481 binding to the PDE7 active site

Interesting in understand the interaction between PDE7 and the BRL 504481, molecular docking studies were conducted to reinforce the interaction capability of the BRL 50481 inhibitor within the active site of the PDE7A enzyme, as previously demonstrated by El-Malah et al. (2024). For methodology validation, the crystallographic ligand complexed with the protein was used for re-docking, yielding an RMSD of 1.92 Å. The re-docking results show that the SwissDock software was able to generate docking positions closely resembling the experimental state. Additionally, a binding energy of −6.220 kcal/mol was observed. The binding energy obtained for the crystallographic ligand supports the findings reported by Arias et al. (2024). The same methodology was applied to the docking of BRL 50481, which resulted in a binding energy of −7.01 kcal/mol. The aromatic ring of BRL 50481 was found to be crucial for its activity, as it was able to interact with three amino acid residues in the enzyme’s active site—valine 380, phenylalanine 416, and phenylalanine 384. Furthermore, this ring also interacted with the inhibitor’s sulfone group. These interactions are consistent with the study by Elfeky et al. (2020), who performed molecular docking of BRL 50481 with PDE7. The interaction pattern of the most favorable BRL 50481 position within the PDE7 active site is demonstrated in Supplementary Figure S3.

### 3.3 PDE7 inhibition decreased the metabolic cellular viability in A2780 and OVCAR3 cell lines

High PDE7A expression in tumors has been associated with poor prognosis (Hao and Yu, 2017). Selecting suitable cell line models can maximize the relevance of experiments (McCabe et al., 2023), as identifying cell lines with alterations in PDE7A expression may provide insights into the clinical aspects of ovarian cancer. To better address this issue, we analyzed the effects of PDE7 inhibitor in combination with PTX in 2 cell lines with different cisplatin sensitivities: A2780 (Beaufort et al., 2014), a cisplatin-sensitive cell line isolated from an untreated patient, and OVCAR3 (Hamilton et al., 1983), a cell line established from the malignant ascites of a patient with progressive ovarian adenocarcinoma following combination chemotherapy with cyclophosphamide, adriamycin, and cisplatin.

**FIGURE 2:**
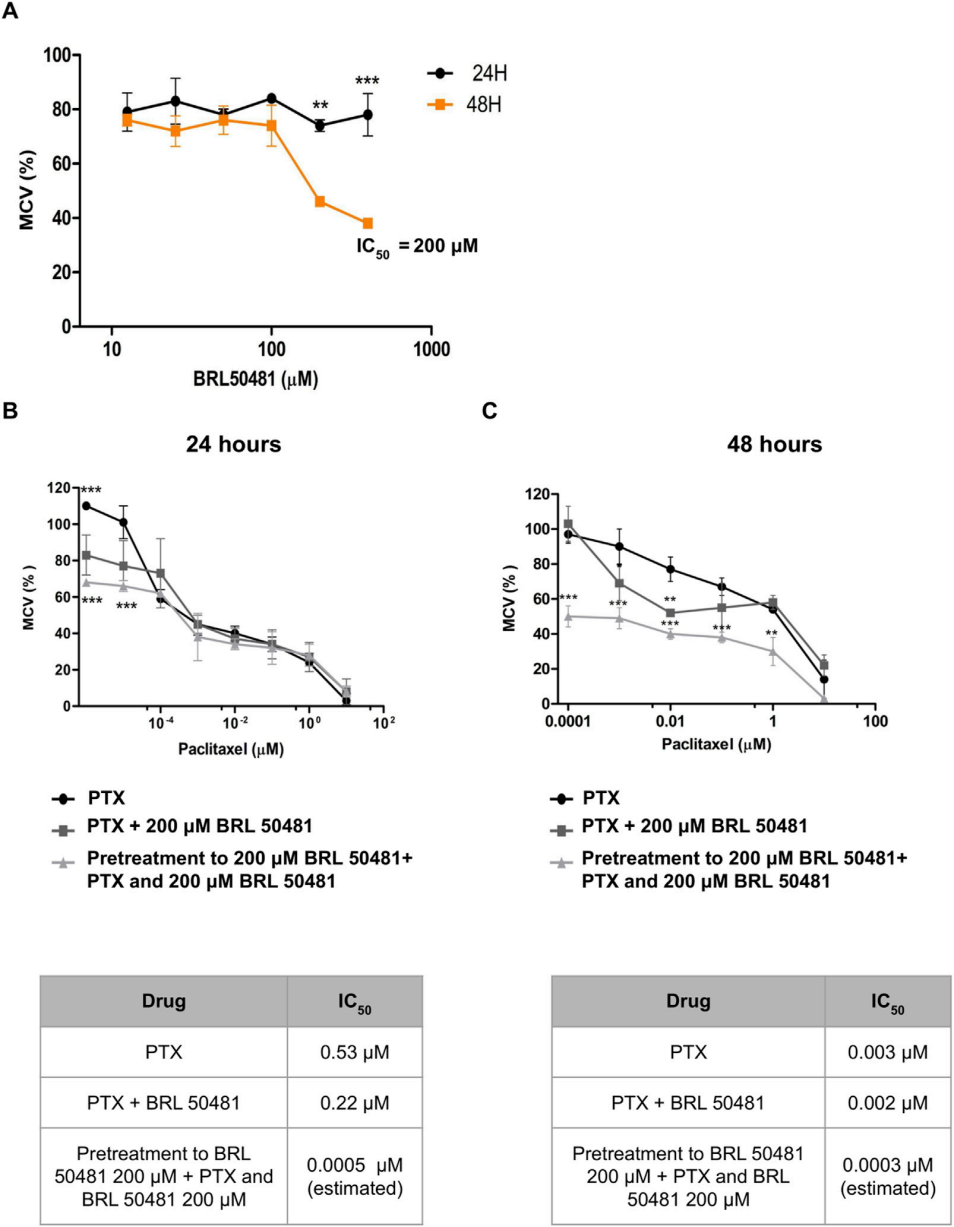
Effect of BRL 50481 and PTX on metabolic cellular viability of A2780 cells. **(A)** Cells were treated with BRL 50481 in monotherapy at 24 and 48 h. Additionally, the cells were treated with PTX as a monotherapy, PTX combined with 200 μM BRL 50481, or pretreated with 200 μM BRL 50481 followed by the combined treatment of PTX and 200 μM BRL 50481 for **(B)** 24 h and **(C)** 48 h. Range of PTX concentration used at 24 h treatment was 10 a 0.0001 μM and at 48 h 10 a 0.000001 μM. The experiments were performed in technical replicates. For each experiment, four biological replicates were performed. The mean and standard deviation are shown on the graphs. Statistical analysis: Two-way ANOVA test followed by Bonferroni post-test. *p < 0.05, **p < 0.01 ***p < 0.001.

**FIGURE 3:**
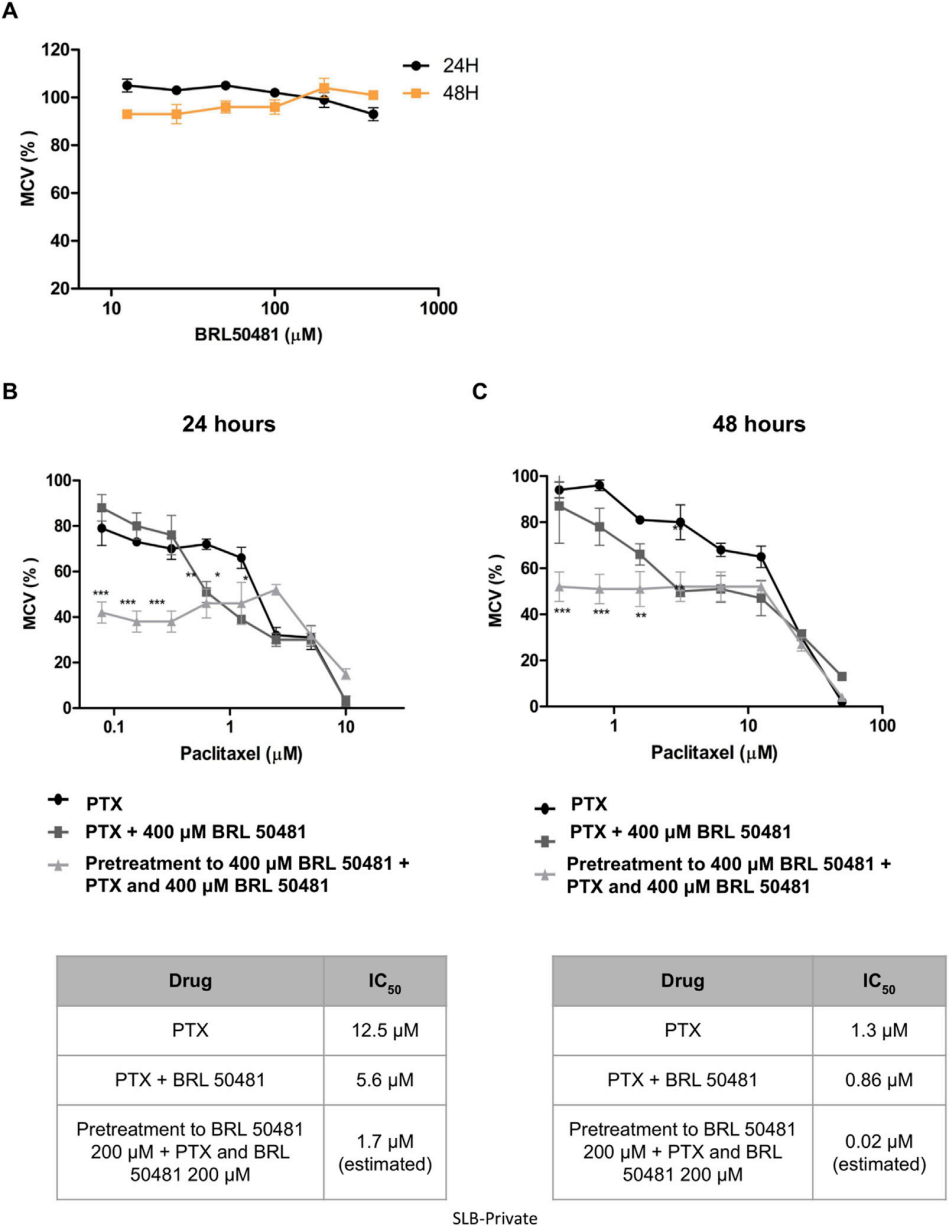
Effect of BRL 50481 and PTX on metabolic cellular viability of OVCAR3 cells. **(A)** Cells were treated with BRL 50481 in monotherapy at 24 and 48 h. Additionally, the cells were treated with PTX as a monotherapy, PTX combined with 400 μM BRL 50481, or pretreated with 400 μM BRL 50481 followed by the combined treatment of PTX and 400 μM BRL 50481 for **(B)** 24 h and **(C)** 48 h. The range of PTX concentration used at 24 h treatment was 50 a 0.390 μM and 48 h 10 a 0.078 μM. The experiments were performed in technical replicates. For each experiment, four biological replicates were performed. Mean and standard deviation are shown on the graphs. Statistical analysis: Two-way ANOVA test followed by Bonferroni post-test. *p < 0.05, **p < 0.01 ***p < 0.001.

**FIGURE 4:**
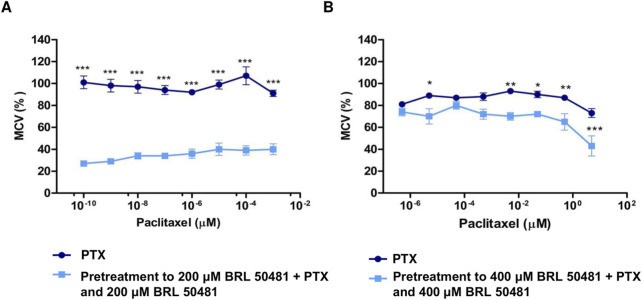
Effect of PDE7 Inhibitor on metabolic cellular viability prior to PTX treatment. **(A)** A2780 cells were treated with PTX (10^−9^ to 10^−16^ M) as monotherapy or pretreated with 200 μM BRL 50481 followed by the combined treatment of PTX and BRL 50481 for 24 h. All concentrations showed statistical significance. ***p < 0.001. **(B)** OVCAR3 cells were treated with PTX (5 μM–5 × 10^−7^ μM) as monotherapy or pretreated with 400 μM BRL 50481 followed by the combined treatment of PTX and BRL 50481 at 24 h. The experiments were performed in technical replicates. For each experiment, four biological replicates were performed. Mean and standard deviation are shown on the graphs. Two-way ANOVA tests followed by Bonferroni post-test were run. *p < 0.05, **p < 0.01 and ***p < 0.001.

Aiming the elucidation of the role of PDE7A in EOC cell lines, we first performed the analysis of metabolic cellular viability (MCV) of the cells using the PDE7 inhibitor BRL 50481 (Tocris Bioscience, Bristol, United Kingdom). The compound BRL 50481 decreased A2780 MCV by approximately 60% comparing 24 and 48 h of treatment (p < 0.01) (Figure 2A). The effect of BRL 50481 in monotherapy was dose-dependent; A2780 cells proved to be more sensitive to the inhibitor than OVCAR3 (Figures 3, 4). The IC_50_ value calculated was 200 μM to A2780 after 48 h of treatment with BRL 50481 (Figure 2A). For OVCAR3 cells, the IC_50_ value could not be determined. Therefore, a concentration of 400 μM, the maximum permissible level to avoid exceeding the DMSO diluent concentration, was selected for cell treatment (Figure 3A). We also calculated the IC_50_ values for PTX in these cell lines and found 0.53 μM to A2780 and 12.5 μM to OVCAR3 after 24 h of treatment (Figures 2B, 3B).

We then combined the PDE7 inhibitor and PTX, a drug commonly used in ovarian cancer treatment (Boyd and Muggia, 2018). The combination of BRL 50481 (200 μM) with PTX (0.53 μM) in A2780 cells resulted in a decrease of PTX IC_50_ of about 2.4-fold after 24 h of treatment (0.53 μM vs. 0.22 μM; p < 0.05) (Figure 2B). However, the same was not observed after 48 h (Figure 2C), suggesting that the treatment efficacy and the drug’s potency are time dependent. Interestingly, the pretreatment with BRL 50481 (200 μM) and post-treatment with the combination of BRL 50481 (200 μM) and PTX reduced PTX IC_50_ to a thousand-fold following 24 h (0.53 μM vs. 0.0005 μM; p < 0.01), and 10-fold after 48 h (0.003 μM vs. 0.0003 μM; p < 0.01) of cells exposure to the drugs.

In OVCAR3, the combination of BRL 50481 (400 μM) with PTX reduced PTX IC_50_ by 2.2-fold after 24 h of cells’ treatment (12.5 μM–5.6 μM; p < 0.01) (Figure 3B). Pretreatment with BRL 50481 (400 μM) followed by the association of BRL 50481 (400 μM) with PTX was also effective, PTX IC_50_ being decreased by 7- and 65-fold following 24- and 48-h treatment, respectively (Figures 3B,C) (p < 0.001).

To determine whether the MCV of the cells could remain suppressed even at lower PTX concentrations than previously used, A2780 and OVCAR3 cells were pretreated with BRL 50481 for 24 h, followed by a combined treatment with reduced PTX concentrations. We observed a significant reduction in PTX IC_50_ in both cell lines. In A2780, even though using the concentration range 10^−3^ μM to 10^−10^ μM, the pretreatment resulted in a reduction in MCV of around 70% in the presence of 10^−10^ μM PTX (p < 0.001); a concentration in which 100% of the cells remain metabolically active when PTX was used alone. The estimated IC_50_ value was 4.86 x 10–^11^ μM (vs. 0.01134; p < 0.001) (Figure 4A). Similarly, the pretreatment with BRL 50481 (400 μM) followed by the association with BRL 50481 (400 μM) and PTX (5 μM–5 x 10^−7^ μM) resulted in an approximately 20% reduction in OVCAR3 MCV compared to treatment with PTX alone (5 × 10^−6^ μM; p < 0.001). The estimated IC_50_ value was 1.9 μM (vs. 12.32 μM; p < 0.01) (Figure 4B).

The effect of combining the PDE7 inhibitor with PTX on MCV was found to be time-dependent across both cell lines, with the 24-h treatment showing the most substantial benefits *in vitro*. Moreover, our findings emphasized the critical role of treatment timing in ovarian cancer therapy, enabling a reduction in PTX concentration, which could potentially minimize toxicity for patients. It should be noted that the estimated IC_50_ calculated through extrapolation was only intended to demonstrate the efficacy of the pretreatment in reducing the IC_50_ concentration.

### 3.4 Inhibition of PDE7 modulates PI3K/AKT/mTOR signaling pathway in EOC

Regarding the PI3K/AKT/mTOR pathway, which is typically overactivated in ovarian cancer (Mabuchi et al., 2015), it represents a regulatory mechanism controlling the activity of pro- and anti-apoptotic Bcl-2 family proteins. This regulation can influence the signaling outcome of the mitochondrial apoptosis pathway. AKT activation leads to Bcl-2 phosphorylation, an essential regulator of apoptosis (Mabuchi et al., 2015; Hassan et al., 2014), and the permeabilization of the mitochondrial outer membrane is associated with the release of mitochondrial proteins into the cytosol and, consequently, cell death. Additionally, the potential of inhibition of the catalytic subunit C of PKA by PDE7A, which has been linked to mitochondrial dysfunction, has been previously reported (Özeş et al., 2018). Thus, we investigate the possible correlation between modulation of this pathway and apoptosis in EOC cells.

**FIGURE 5:**
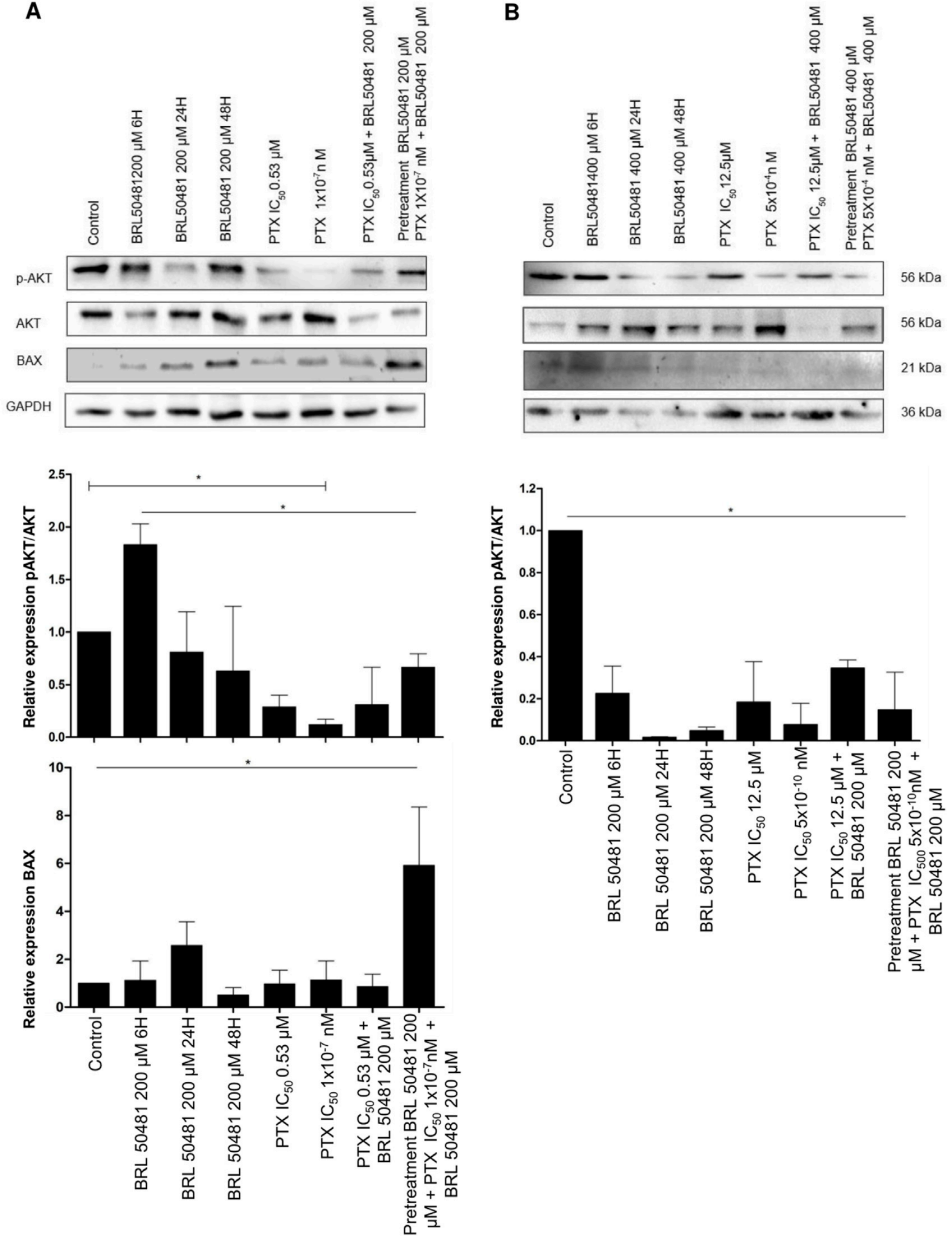
PI3K/AKT/mTOR pathways and apoptosis modulation after PDE7 inhibition. A2780 **(A)** and OVCAR3 **(B)** cells were incubated with BRL 50481 in monotherapy at different times (6, 24 and 48 h), PTX as monotherapy (24 h), PTX plus BRL 50481 (24 h), and pretreated with BRL 50481 (24 h), followed by treatment with PTX plus BRL 50481 at another 24 h. GAPDH was used as a loading control. Experiments were performed in biological triplicate, except for BAX (n = 2 for OVCAR3 cells). Statistical analysis ANOVA with Bonferroni post-test. *p < 0.05.

PI3K pathway activation was seen via the increase in AKT phosphorylation after 6 h compared to 24 and 48 h treatment of A2780 cells with 200 μM BRL 50481 (2.3-fold; 2.4-fold, respectively; p < 0.05), PTX 0.53 μM and 1x10^−7^ nM in monotherapy (5.2-fold; 12.5-fold, respectively; p < 0.001), the combination of 200 μM BRL 50481 and 0.53 μM PTX (4.8-fold; p < 0.001) and the pretreatment with 200 μM BRL 50481 followed by the association with BRL 50481 (200 μM) and PTX (1x10^−7^ nM) (2.25-fold; p < 0.05). Nonetheless, the opposite was noted following the treatment of A2780 with the combined treatment of BRL 50481 (200 µM) and PTX (0.53 µM) (Figure 5A).

Conversely, the PI3K pathway was inhibited under all experimental conditions in OVCAR3 (Figure 5B). Comparing the control (DMSO-treated condition) with the treatment with BRL 50481 400 µM by 6, 24 and 48 h led to a decrease in AKT phosphorylation (4.5, 5.0, 3.0-fold, respectively; p < 0.01), indicating a time-dependent treatment. Also, the treatment with PTX (12.5 μM and 5 × 10^−4^ nM) in monotherapy promoted a decrease in AKT phosphorylation by 5.4 and 12.8-fold, respectively (p < 0.01). As expected, the combination with BRL 50481 (400 μM) and PTX (12.5 μM) and the pretreatment with BRL 50481 (400 μM) followed by the association with BRL 50481 (400 μM) and PTX (5 × 10^−4^ nM) promoted a decrease in AKT phosphorylation by 2.9 and 6.8-fold, respectively (p < 0.01) (Figure 5B).

Therefore, we evaluated the expression of the pro-apoptotic protein BAX, an important member of the Bcl-2 family. We found that the pretreatment of A2780 with 200 μM BRL 50481 followed by the association with BRL 50481 (200 μM) and PTX (1 × 10^−7^ nM) led to a 6-fold increase in BAX expression compared to control (p < 0.01); PTX 0.53 μM in monotherapy (p < 0.01) and the combination of 200 μM BRL 50481 and PTX 0.53 μM (p < 0.01); 5.2-fold compared to PTX 1 × 10^−7^ nM (p < 0.01). The high level of BAX was also found in the cells treated with 200 μM BRL 50481 in monotherapy for 6, 24 and 48 h (5.2, 2.3 and 11.7-fold, respectively; p < 0.01) (Figure 5A). Thus, the BAX increase in A2780 cell can be explained, at least partially, by the decreased AKT phosphorylation and the consequent upregulation of an apoptosis pathway modulator observed when the cells were pretreated with the PDE7 inhibitor, followed by the combined treatment with PTX and BRL 50481.

Preliminary results revealed that in A2780 cells, 0.53 µM PTX significantly increased hypodiploid cells compared to the control and BRL 50481, with no additional effect from the combination treatment. In OVCAR3 cells, the combination of 400 µM BRL 50481 and 12.5 µM PTX significantly increased hypodiploid cells and PI+/Annexin V+ cells compared to the control (p < 0.05), indicating necrosis. Further studies are needed to clarify the cell death mechanism in both cell lines (Supplementary Figures S4, S5).

### 3.5 PDE7 inhibition controls cytokines gene expression in EOC microenvironment

**FIGURE 6:**
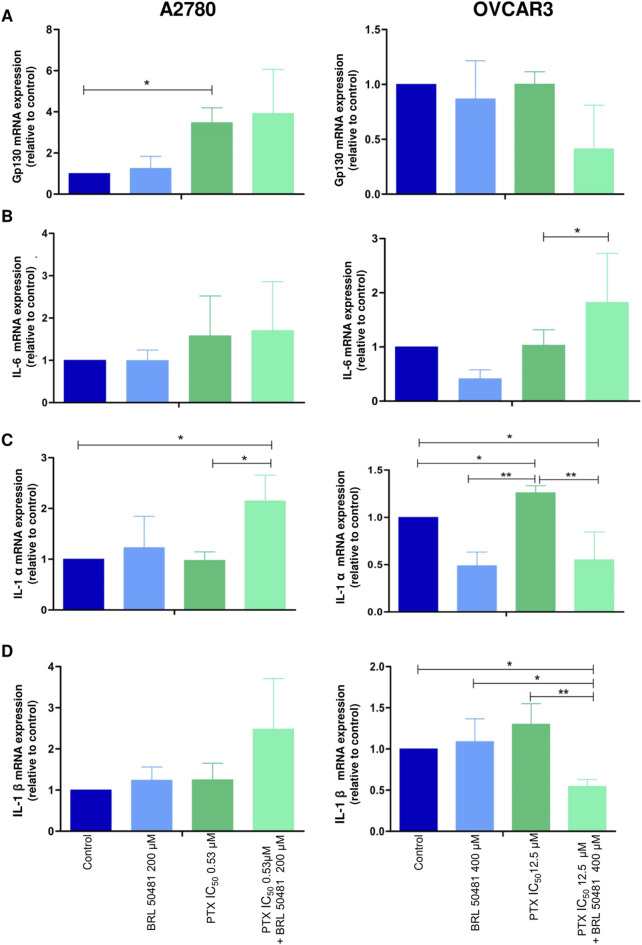
Effect of PD7 inhibition on cytokines **(A)** gp130, **(B)** IL-6, **(C)** IL-1α and **(D)** IL-1β modulation in A2780 and OVCAR3 cells. Total RNA was obtained from the cells treated with BRL 50481 or PTX, both in monotherapy, at 24 h and pretreated with BRL 50481 (24 h) following the combination of BRL 50481 plus PTX at another 24 h. GAPDH was used as a housekeeping gene. The relative quantification of gene expression was performed using the 2^−ΔΔCT^ method. All experiments were run with at least three biological replicates and data are shown as mean ± SD. Statistical analysis one-way ANOVA followed by the Bonferroni post-test. *p < 0.05; **p < 0.01.

**FIGURE 7:**
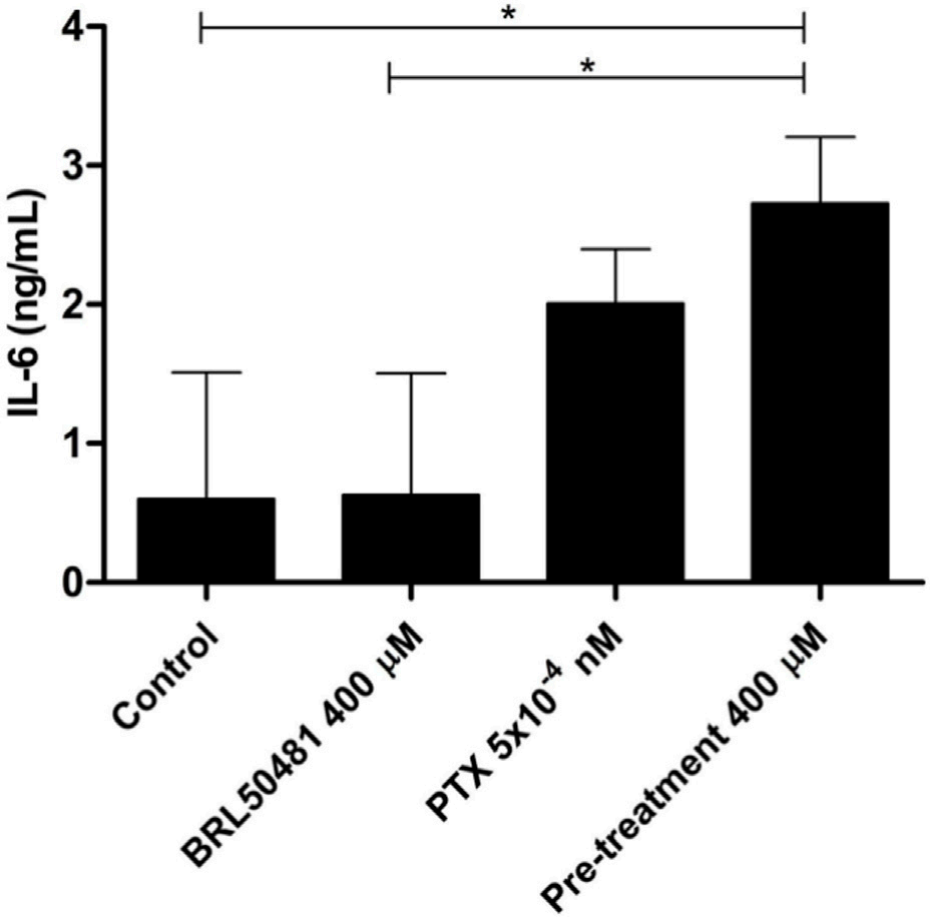
IL-6 cytokine level was measured in the supernatant of cells by ELISA. OVCAR3 cells were pretreated with 400 μM BRL 50481 at 24 h followed by the combination of 400 μM BRL 50481 plus PTX 5 × 10^−10^ μM at another 24 h. An increased expression of IL-6 of about 3.85-fold was observed in the cells treated with 400 μM BRL 50481 compared to untreated cells. All experiments were performed in triplicate and data are shown as mean ± SD. Statistical analyzes were done by one-way ANOVA followed by the Bonferroni post-test. *p < 0.05.

Considering the pivotal role of inflammation and immune components in shaping the tumor microenvironment, we investigated how PDE7 inhibition influences cytokine modulation within the HGSOC microenvironment, aiming to uncover its potential impact on tumor-immune interactions (Figures 6, 7).

Although A2780 pretreated with 200 µM BRL 50481 at 24 h followed by the treatment of cells with 200 µM BRL 50481 and 1 x 10^−7^ nM PTX enhanced approximately 4-fold the mRNA expression of glycoprotein 130 (gp130) compared to control (p < 0.05) (Figure 6A), no difference was observed regarding IL-6 mRNA expression (Figure 6B). To better understand the role of this cytokine in the A2780 cell, we should investigate key molecules involved in the signaling cascade, such as the expression of IL-6Rα and STAT3, as well the cytokine signaling suppressor 3 (SOCS3), a molecule that inhibits IL-6-mediated signaling through a negative feedback mechanism. A differential pattern was noted in OVCAR3, in which the pretreatment of cells with 400 µM BRL 50481 followed by the combination of 400 µM BRL 50481 and 5 x 10^−4^ nM PTX increased IL-6 mRNA expression by 2-fold compared to BRL 50481 (p < 0.05) (Figure 6B). The increase of IL-6 mRNA expression was translated into higher protein expression in OVCAR3 by 3.85-fold when compared to cells that received 400 µM BRL 50481 as monotherapy (p < 0.05) (Figure 7).

Concerning IL-1α, the pretreatment with 200 μM BRL 50481 at 24 h, followed by the combination of 200 μM BRL 50481 and 1 x 10^−7^ nM PTX increased its expression 2-fold compared to control and 1x10^−7^ nM PTX (p < 0.05) in A2780 (Figure 6C). In contrast, OVCAR3, both the treatment with 400 μM BRL 50481 at 24 h and pretreated with 400 μM BRL 50481 at 24 h followed by the combination with 400 µM BRL 50481 and 5 × 10^−4^ nM PTX at another 24 h reduced by 2-fold compared to control (p < 0.05), while 5 x 10^−4^ nM PTX increased 3-fold its expression (p < 0.01) (PTX vs. BRL 50481 and PTX vs. pretreatment) (Figure 6C). The same was observed for interleukin IL-1β (Figure 6D). The pretreatment scheme decreased IL-1β gene expression by 2-fold in comparison to control (p < 0.05), 5 × 10^−4^ nM PTX (p < 0.01) and 400 µΜ BRL 50481 (p < 0.05). No difference in IL-1β expression was found in A2780 cells.

### 3.6 Effect of PDE7 inhibition on epithelial plasticity and cancer stem cells

**FIGURE 8:**
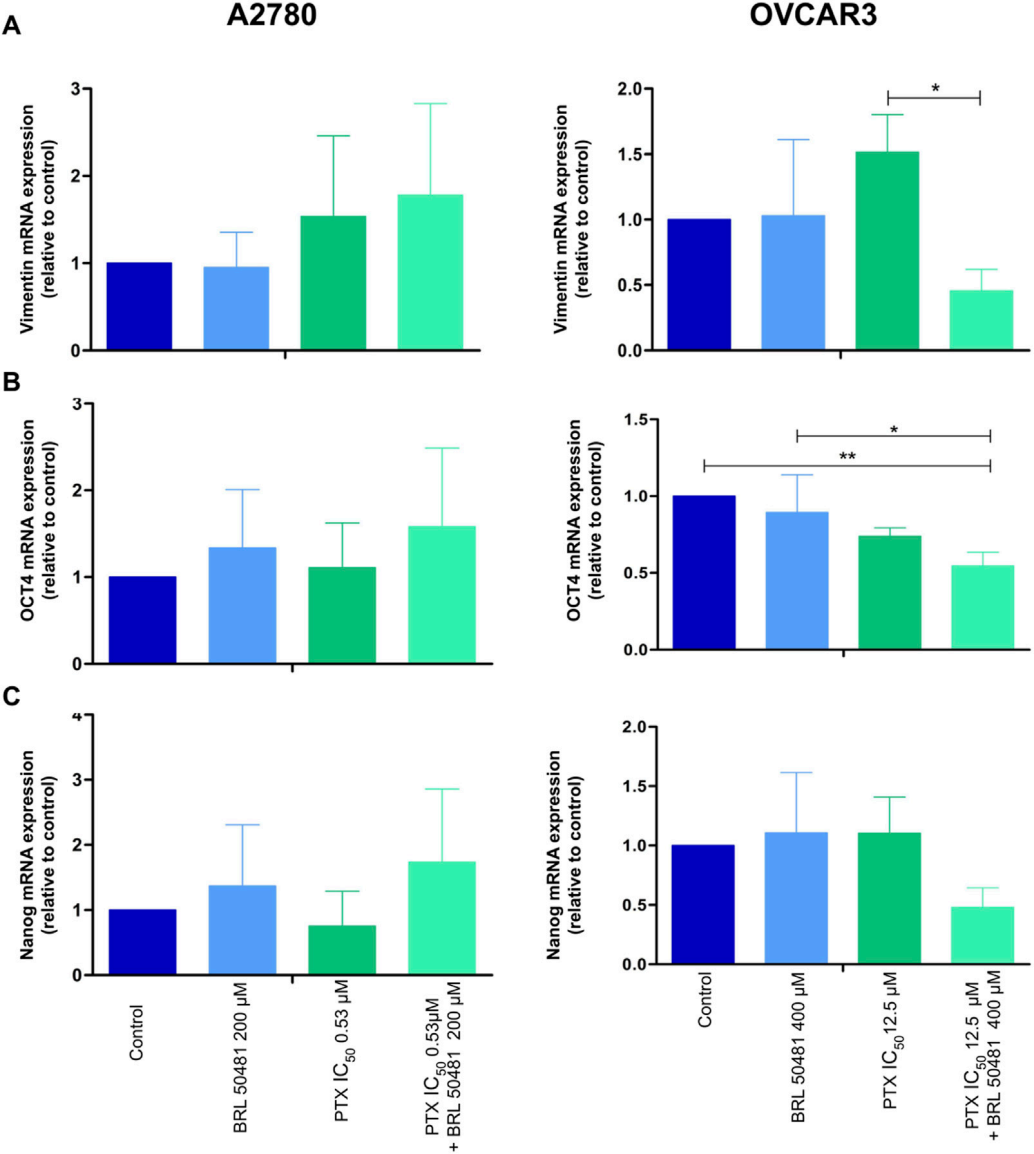
Gene expression analysis of epithelial-mesenchymal transition **(A)** vimentin and cancer stem cell markers **(B)** OCT4 and **(C)** NANOG in A2780 and OVCAR3 cells. The samples were treated with BRL 50481 or PTX, both in monotherapy, at 24 h or pretreated with BRL 50481 at 24 h followed by treatment with BRL 50481 plus PTX at another 24 h. The relative quantification of gene expression was performed using the 2^−ΔΔCT^ method. GAPDH was used as a housekeeping gene. All experiments were run with at least three biological replicates and data are shown as mean ± SD. Statistical analysis one-way ANOVA with Bonferroni post-test. *p < 0.05; **p < 0.01.

Frequently, the tumor loses its epithelial characteristics and acquires a mesenchymal pattern in a phenomenon that contributes to cancer cells’ invasion and metastasis called Epithelial-Mesenchymal Transition (EMT) (Davidson et al., 2012). The pretreatment of OVCAR3, but not of A2780, with 400 μM BRL 50481 followed by the combined treatment with 400 μM BRL 50481 and 5 × 10^−4^ nM PTX decreased vimentin expression by 2-fold compared to 5 × 10^−4^ nM PTX as monotherapy (Figure 8A).

We also evaluated cancer stem cell markers OCT4 and NANOG. In OVCAR3, the pretreatment of cells with 400 μM BRL 50481 followed by the treatment with 400 μM BRL 50481 and 5 × 10^−4^ nM PTX decreased OCT4 mRNA expression by 2-fold compared to control (p < 0.01) and by 1.6-fold compared to 400 μM BRL 50481 as monotherapy (p < 0.05) (Figure 8B). Moreover, there was a decreasing trend in NANOG expression compared to the control group (Figure 8C). No significant changes of these markers were observed in A2780 (Figure 8).

The outcomes suggest that PDE7 inhibition can modulate phenotypic traits involved in ovarian tumorigenesis. And the mechanisms involved in the decrease in MCV in both cells are different. To gain a deeper understanding of this complex phenotype, further evaluation of gene expression related to additional cancer stem cell markers, such as CD133, SOX2, aldehyde dehydrogenase, and CD44 could provide new insights.

### 3.7 The inhibition of PDE7 altered the morphology of the cells and the mitochondrial ridges

**FIGURE 9:**
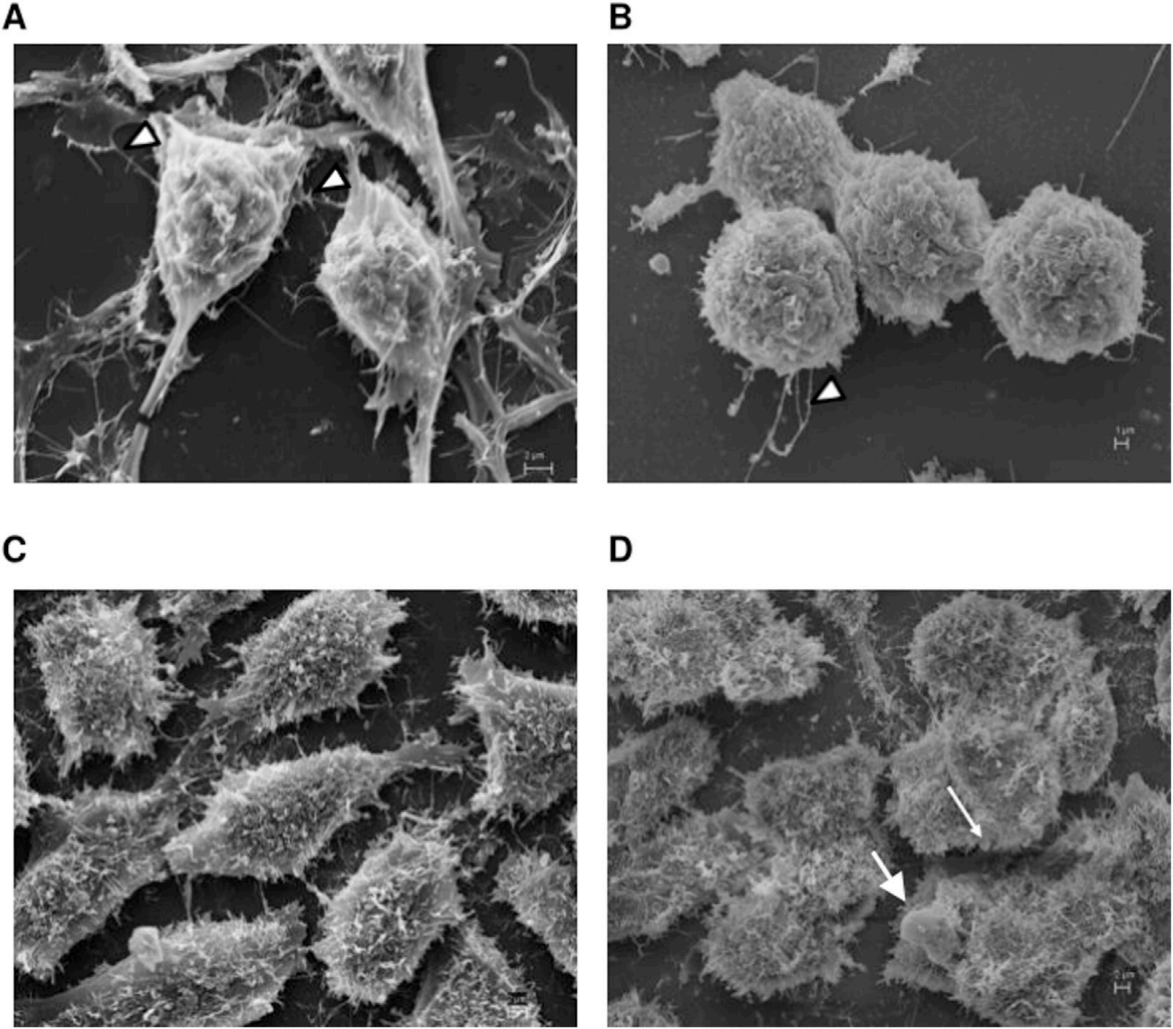
Scanning electron microscopy analysis. A2780 **(A,B)** and OVCAR3 **(C,D)** cells were seeded at the density 0.4 × 10^6^ cell/mL in 12 well plates under their own coverslip and treated with DMSO (control **(A,C)**) and pretreated with BRL 50481 at 24 h followed by treatment with BRL 50481 plus PTX at another 24 h **(B,D)**. The observed filaments are usually related to tumor spreading. In **(B)**, more rounded cells with significant lower incidence of attaching filaments (arrowheads) in comparison with **(A)** were noted. In **(D)**, we observed more rounded cells and the presence of membrane blebbing, which is typical of apoptotic cells. The images were analyzed using Electron Microscope Scanning (ZEISS EVO 40 XVP). Bar scale is 2 μm.

Motivated in assessing whether the inhibition of PDE7 could alter the cell morphology, EOC cells were analyzed by Scanning Electron Microscopy (SEM) and Transmission Electron Microscopy (TEM). A2780 pretreated with the PDE7 inhibitor showed a dramatic change in the cell surface morphology in comparison to untreated cells (Figures 9A,B). Cells acquired a spherical shape and had fewer microvilli, which can cause the detachment of the cells from the matrix, leading to cell death. When OVCAR3 was analyzed, the pretreatment scheme of the cells promoted subtler alterations in their morphology compared to the untreated sample (Figures 9C,D). This result is in agreement with the MCV data, in which the A2780 was more sensitive to the pretreatment with BRL 50481 compared to OVCAR3 (Figures 2, 3).

**FIGURE 10:**
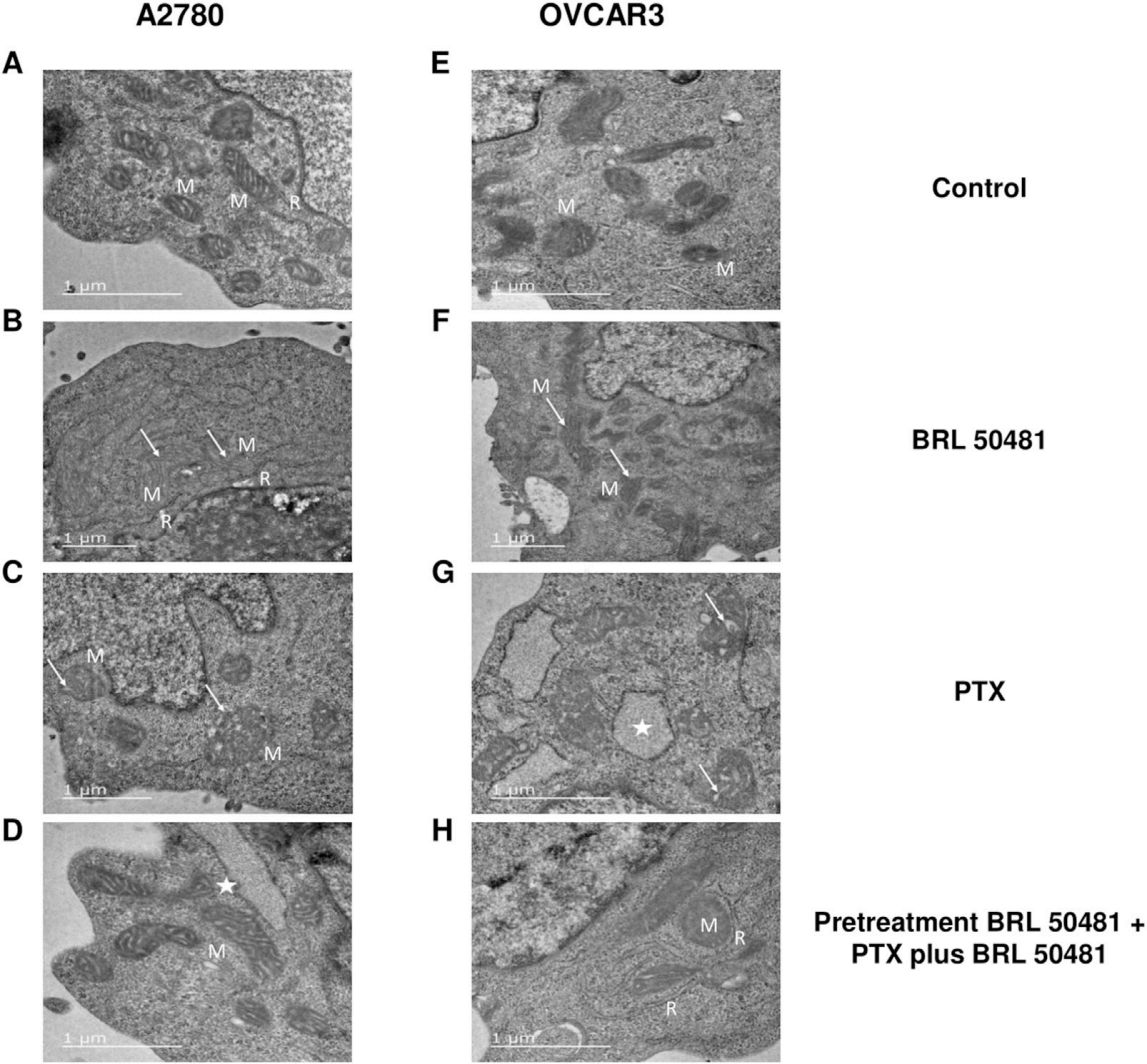
Ultrastructural analysis by transmission electron microscopy. A2780 **(A–D)** and OVCAR3 **(E–H)** cells were seeded at the density of 0.4 x 10^6^ cells/mL in 12 well plates. In A2780 control condition **(A)**, distinct profiles of mitochondria (M) in association with rough endoplasmic reticulum (R) were observed. A2780 cells treated with PTX for 24 h **(B)** resulted in enlarged mitochondria (M) with cristae (arrow). Note the higher amount of rough endoplasmic reticulum (R) in association with mitochondria in comparison to control cells. When cells were treated with BRL 50481 for 6 h **(C)**, rounded-shaped mitochondria (M) with altered and enlarged cristae (arrow). Pretreated cells with BRL 50481 **(D)** had a mitochondria (M) pattern as the control condition. However, larger vacuoles with structures like ribosomes were observed in their periphery, resembling an enlarged reticulum profile (star). As for the OVCAR3 cells’ control condition, normal mitochondria (M) were seen **(E)**. When cells were treated with PTX for 24 h, mitochondria presenting numerous and larger internal cristae were observed (arrow) **(F)**. In turn, the treatment of cells with BRL 50481 for 6 h resulted in altered mitochondria with rounded shape and enlarged internal cristae (arrow) **(G)**. Larger vacuoles, resembling enlarged rough endoplasmic reticulum (R) with ribosomes attached to their periphery, were noted close to the mitochondria (M) (star). Pretreated cells with BRL 50481 followed by treatment with BRL 50481 and PTX resulted in the occurrence of mitochondria (M) similarly to the control condition, but enlarged reticulum (R) was observed in close association with mitochondria (M). The images were analyzed using a Transmission Electron Microscope (JEOL -1,400 Plus). Bar scale in 1 μm.

TEM experiments revealed that the treatment of A2780 with 1 × 10^−7^ nM PTX increased the number of endoplasmic reticulum units in the cells (Figure 10). Untreated cells are demonstrated in Figure 10A. Both experimental conditions with 200 μM BRL 50481 for 6 h in monotherapy and the pretreatment of cells with 200 μM BRL 50481 followed by the treatment with 200 μM BRL 50481 and 1 × 10^−7^ nM PTX changed mitochondrial morphology, as well as promoted the enlargement of the mitochondrial ridges (Figures 10B–D). These findings are consistent with the damage caused by the combination of BRL 50481 and PTX in A2780 cells, also observed by the MCV assay (Figure 2).

In turn, no significant changes in cell ultrastructure were noted in OVCAR3 treated with 5 × 10^−4^ nM PTX. However, it is worthwhile to point out that there was a significant increase in the number of mitochondria units compared to the control (Figures 10E–G). As observed in A2780, the treatment of cells with 400 μM BRL 50481 for 6 h and the pretreatment of cells with 400 μM BRL 50481 followed by the treatment of cells with 400 μM BRL 50481 and 5 × 10^−4^ nM PTX changed mitochondria morphology (Figures 10F,H). Furthermore, we also observed an association between the endoplasmic reticulum and mitochondria. Nonetheless, the occurrence of apoptotic bodies needs to be better investigated.

## 4 Discussion

The present work is pioneering in showing higher expression of *PDE7A* in HGSOC samples compared to fallopian tubes. Intrigued by the findings, we pursued the investigation of the PDE7A role in EOC. PDE isoforms inhibitors have already been used in the treatment of asthma, chronic obstructive pulmonary disease, pulmonary hypertension and multiple sclerosis (Houslay et al., 2005; Naviglio et al., 2009), demonstrating that this class of drugs might be safe for human use. In cancer, the role of PDE isoforms in cell survival and motility in different tumors, such as prostate (Henderson et al., 2014; Powers et al., 2015), breast (Nidhyanandan et al., 2015), leukemia (Dong et al., 2010) and colon (Ahmad et al., 2015) has been shown. The synergistic effect was observed when combining adenosine A2A receptor agonists with cAMP-hydrolyzing PDE inhibitors in multiple myeloma and diffuse large B cell lymphoma cell lines, as well as in primary patient samples (Rickles et al., 2010). In addition, cAMP-hydrolyzing PDE inhibitors, such as dipyridamole and cilostazol, increased cAMP levels and potentiated statin-induced apoptosis in hematological disease (Longo et al., 2020). These findings support our results, showing that the PDE7 inhibitor BRL 50481 combined with the chemotherapy drug paclitaxel decreased EOC cell viability even at concentrations lower than those used clinically, which could cause severe neurotoxicity in patients.

Regarding the chronology of the treatment, our study showed that the pretreatment of EOC cells with the PDE7 inhibitor, followed by its combination with PTX was more efficient in controlling tumorigenesis, opening the discussion about the therapeutic scheme in cancer treatment. Several studies have demonstrated the effect of selective PDE inhibitors. For example, the use of selective PDE4D inhibitors, NVP-ABE171 and cilomilast, in prostate cancer, both *in vitro* and *in vivo*, promoted a decrease in cell proliferation (Powers et al., 2015). In addition, rolipram and Ro-20-1724, PDE4 inhibitors, provided suppression of chemotaxis in colon cancer cells (Murata et al., 2000). In consonance, roflumilast induced apoptosis and inhibited the proliferation of cisplatin-resistant OC cells (Gong et al., 2018). Experiments *in vivo* need to be conducted to demonstrate the effects of PDE7A inhibition on tumor progression and to evaluate the safety of the combination treatment with PTX.

Studies have shown that AKT has been frequently hyperactive in cisplatin-resistant EOC by inhibiting the phosphorylation of p53 (Fraser et al., 2008). Abnormal expression of AKT or its activation may also confer resistance to the PTX (Page et al., 2000). Furthermore, PI3K inhibition increased the efficacy of the treatment of epithelial EOC (Hu et al., 2002). Thus, there is a huge demand for the rational development of PI3K/AKT/mTOR pathway inhibitors, particularly for cancer subtypes with alterations in this pathway that could benefit from combination therapy (Guimarães et al., 2015). Although the PDE7 inhibitor negatively modulated PI3K/ATK pathway in OVCAR3 cells, this did not reflect on the regulation of the pro-apoptotic molecule BAX. This molecule migrates to the mitochondria (Wolter et al., 1997), releasing the cytochrome C (Jurgensmeier et al., 1998), and activating the dimerization of the apoptotic protease 1 activation factor (APAF-1) and, consequently, the apoptosis cascade. In A2780 cells, pretreatment with BRL 50481 followed by treatment with both PTXplus BRL 50481 increased BAX expression, indicating that cell death can occur via apoptosis. Studies with PDE inhibitors sildenafil and vardenafil have demonstrated caspase-dependent apoptosis in chronic lymphocytic leukemia (Sarfati et al., 2003). Additionally, Moon and colleagues (2002) (Moon et al., 2002) found that inhibiting PDE3-B and PDE4 increased apoptosis in certain patients with chronic lymphocytic leukemia.

A study demonstrated that HCT116 colon cancer cells treated with 300 µM sildenafil for 48 h showed inhibited proliferation, which correlated with decreased β-catenin levels, increased redox stress, and G1 cell cycle arrest. The results suggest that while higher concentrations of inhibitors over extended periods could cause additional toxicity, direct targeting of specific proliferative pathways likely does not play a significant role in treatment outcomes for colon cancer cell lines. Furthermore, high doses of PDE inhibitors exhibit an antiproliferative effect as an off-target action of these drugs, while specific signaling pathways have been implicated in this response. The primary mechanism involves the cGMP/PKG pathway, which downregulates β-catenin protein levels, leading to reduced expression of TCF-target genes. Additionally, inhibition of the AKT and ERK pathways has been observed in cells expressing ectopic PKG2 (Hou et al., 2022).

Preliminary results suggest that necrosis may be a mechanism of cell death in OVCAR3 cells. Recent evidence indicates that the pro-apoptotic proteins BAX and Bcl-2 homologous antagonist/killer (BAK) are essential for necrosis, as they regulate the permeability of the outer mitochondrial membrane (Karch and Molkentin, 2015; Whelan et al., 2012). Also, during necrosis, the mitochondria become dysfunctional, in part through the opening of the mitochondrial permeability transition pore (Karch and Molkentin, 2015). Our TEM results showed alterations in mitochondrial morphology as well as the enlargement of the mitochondrial ridge. All these findings led us to hypothesize that one possible mechanism for PDE7 inhibition involves mitochondrial damage and ATP release.

Cytokines are involved in several physiological processes such as inflammation (Hansson, 2005), cell migration (Moser et al., 2004), angiogenesis (Belperio et al., 2000) and apoptosis (Janes et al., 2005). The pretreatment of OVCAR3 with BRL 50481 releases IL-6 in the supernatant compared to untreated cells. Regarding the pleiotropic effects of IL-6, one aspect is becoming clear: its action is influenced by tissue homeostasis and the type of cell producing it (Mauer et al., 2015). In the canonical signaling pathway, IL-6 interacts with gp130, which associates with interleukin-6 receptor subunit alpha (IL-6Rα), initiating the activation of the intracellular Janus kinase (JAK)-signal transducer and activator of transcription (STAT) signaling pathway (Hibi et al., 1990). Increased expression of IL-6Rα and constitutive activation of the STAT3 pathway have been associated with cell proliferation in EOC (Syed et al., 2002), promoting tumorigenesis and metastasis (Browning et al., 2018; Pinciroli et al., 2013). Furthermore, IL-6 is a key interleukin that regulates metabolism and mitochondrial function. Mitochondria, essential for energy metabolism, are implicated in various pathological conditions, including obesity-related insulin resistance, oxidative stress in skeletal muscle, and cancer.

Ji et al. (2011) observed that IL-6 led to decreased mitochondrial membrane potential, decreased cellular ATP production, increased intracellular ROS levels and alterations in mitochondria morphology during lipolysis in adipocytes. The treatment of preadipocytes and adipocytes with IL-6 also promoted mitochondrial fragmentation, altering the fusion state of mitochondrial dynamics (Nisr et al., 2019). The dysregulation of mitochondrial function remains one of the main components of the metabolic reprogramming of cancer, affecting gene expression, cellular differentiation and the tumor microenvironment (Frezza, 2020). Furthermore, Bindra and colleagues demonstrated a negative association between mitochondrial enzyme activities and IL-6 levels in ascites, indicating that as IL-6 levels rise, both mitochondrial function and mass decline. This relationship suggests that mitochondria may influence the inflammatory state of the tumor (Bindra et al., 2021).

In this context, PDE7 inhibition with concomitant treatment with PTX increased the release of IL-6, which may have contributed to changes in mitochondrial morphology and, consequently, the metabolism of tumor cells. This data is supported by MCV data, assays are used to assess metabolic activity, including mitochondrial activity (Bindra et al., 2021). However, further work is needed to better understand the interaction between tumor mitochondria and progression in EOC.

We also evaluated the IL-1 cytokine, important in inflammatory processes. Previous studies indicate that the constitutive production of IL-1β in EOC (Lewis et al., 2006) is linked to invasion (Denkert et al., 2003) and the production of proangiogenic factors, as the endothelial growth factor (Stadlmann et al., 2005). In contrast, IL-1α may primarily function as a suppressor in malignant cells by recruiting immunocompetent cells to the tumor microenvironment and stimulating an immune response to combat the tumor growth (Apte et al., 2006; Dinarello, 2010). IL-1β is recognized as a key proinflammatory molecule, and the low concentration of IL-1β is associated with a limited inflammatory response (Voronov et al., 2013). Evidence shows that the increase of cAMP levels enhances IL-1α and IL-1β mRNA expression in human myelomonocytic cell lines and monocytes (Sung and Walters, 1991). The pretreatment with the BRL 5048 decreased both IL-1α and IL-1β gene expression in OVCAR3, reinforcing that the inhibition of PDE7 and, consequently, the release of cAMP directly affects the inflammatory pathways.

The EOC is a heterogeneous tumor, and the maintenance of epithelial features is important for the success of treatment. Silencing of OCT4 and VIM genes resulted in decreased proliferation, migration, invasion, chemoresistance and tumor progression in embryonal carcinoma cells and embryonic stem cells (Boer et al., 2007; Wei et al., 2009). Our data showed that PDE7 inhibition promoted modulation in both markers, indicating a possible contribution of this treatment against metastasis and tumor recurrence.

We, herein, present a novel target in EOC derived from RNA-sequencing data. The PDE7 inhibition in association with PTX seems potentially beneficial in reducing EOC progression. In addition, the chronology of the treatment was proven to be important for the MCV with concomitant decrease in PTX concentration, demonstrating that the therapeutic scheme enhanced the potency of PTX against HGSOC. Taxane-induced neurotoxicity remains a significant challenge in cancer treatment, often necessitating dose reductions or discontinuation of therapy. Ongoing research is exploring strategies to mitigate these side effects. The association with PDE7 inhibitor and chemotherapy with taxane-based chemotherapy demonstrated, *in vitro,* reduced need for higher doses of PTX. This could lead to a lower risk of neurotoxicity, increased treatment adhesion, and, ultimately, an improved quality of life for EOC patients.

This work shows the effectiveness of PDE inhibitors in EOC cells. However, the IC_50_ values obtained are critical for assessing the relevance of *in vitro* findings. The high concentrations of PDE inhibitors required to directly suppress ovarian cancer cell growth may not be clinically practical, and animal models are required to test this approach. Therefore, modifications to the drug structure might be necessary. Furthermore, it is essential to clarify how PDE7 inhibition influences EOC proliferation through off-target mechanisms, which requires further investigation. These studies are essential for understanding the therapeutic potential of PDE7 in EOC.

## 5 Limitation of the study

STR is unavailable for some cell lines but genotyped. The Gene Ontology (GO) number for the RNA sequencing data and will be assigned later.

## Data Availability

The original contributions presented in the study are included in the article/Supplementary Material, further inquiries can be directed to the corresponding author.

## Supplementary material

SUPPLEMENTARY FIGURE S1 STR documents from OVCAR5, OVCAR8, OVCAR429 and SKOV3 cell lines were provided by GRCF DNA Service.

SUPPLEMENTARY FIGURE S2 Validation of RNA-sequencing data using cell lines by RT-qPCR **(A)** and Western blot **(B)**. FT 2821 sample was used as a reference and the results were normalized by GAPDH. The RT-qPCR analysis was run with three biological replicates and data are shown as mean ± SD. Statistical analysis one-way ANOVA with Tukey Post-test. *p< 0.05. The Western blot analysis was conducted using only one biological replicate. **(C)** Analysis of *PDE7A* expression in The Human Protein Atlas in ovarian cancer cells. As shown, the cell line OVCAR3 exhibits one of the highest expression levels among ovarian cancer cells. RNA expression data as normalized per million (nTPM) values. This plot shows each individual nTPM of the gene in each cell line. n=59 ovarian cancer cells.

SUPPLEMENTARY FIGURE S4 Percentage of hypodiploid cells under different treatment conditions.

SUPPLEMENTARY FIGURE S5 Gate strategy employed for analyzing apoptosis and necrosis events in (A) A2780 and (B) OVCAR3 cells. The experiments were performed on the BD Accuri® C6 Flow Cytometer using the FL-1 channel (530 nm) for Annexin V-FITC and the FL-2 (585 nm) for PI. A total of 10,000 events (cells) were acquired per sample, and the data were analyzed using Summit software v. 4.3.

SUPPLEMENTARY FIGURE S6 Western Blot raw images.
