# Supplementary material for: Phosphodiesterase 7: a potential novel therapeutic target in ovarian cancer
Source: Front Pharmacol. 2025 Jun 4;16:1566330. doi: 10.3389/fphar.2025.1566330 (PMC12174393; doi:10.3389/fphar.2025.1566330)
Supplement: Supplementary file 1 [file DataSheet7.pdf]

S1 Table: Log fold-change, p-value and adjusted p-value from the RNA-sequencing data for the genes selected for RT-qPCR analysis.

Gene	log2.fold_change	p_value	q_value
ATAD2	2,20564	0,00055	0,230529
MUC16	2,04797	1,2114	5,00E-05
PDE7A	2,54617	1,00E-04	0,0978
STMN1	2,76743	5,00E-05	0,0815
CCND2	-2,34213	5,00E-05	0,0815
CRELD2	-2,53722	5,00E-05	0,0815
CYP1B1	-3,12186	5,00E-05	0,0815
CCL2	-2,83886	1,00E-04	0,0978
VNN2	-4,05557	5,00E-05	0,0815
