# Supplementary figures and images for: Phosphodiesterase 7: a potential novel therapeutic target in ovarian cancer
Source: Front Pharmacol. 2025 Jun 4;16:1566330. doi: 10.3389/fphar.2025.1566330 (PMC12174393; doi:10.3389/fphar.2025.1566330)

S2 Figure

A

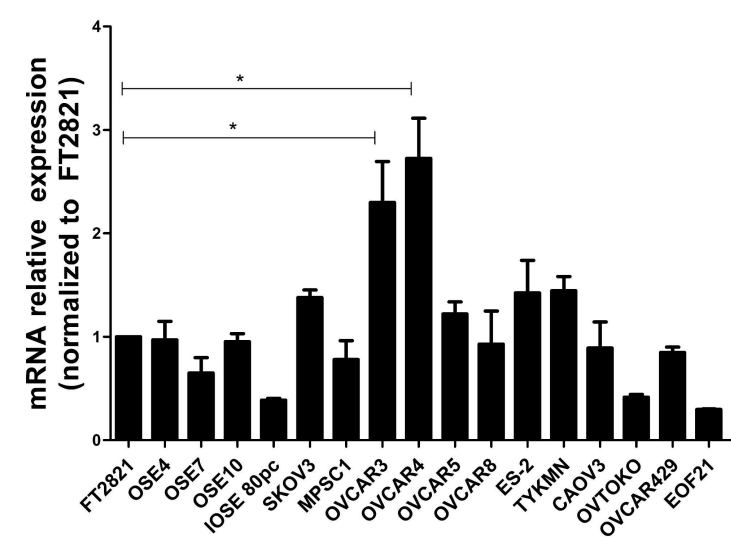

B

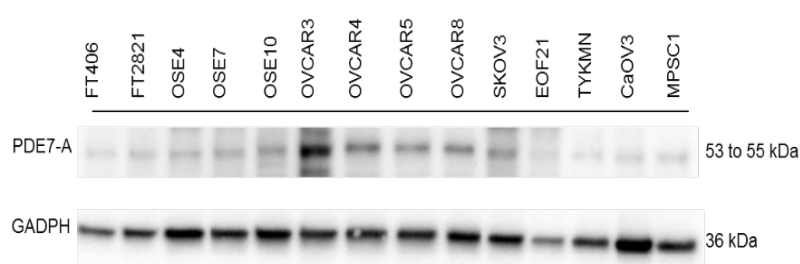

C

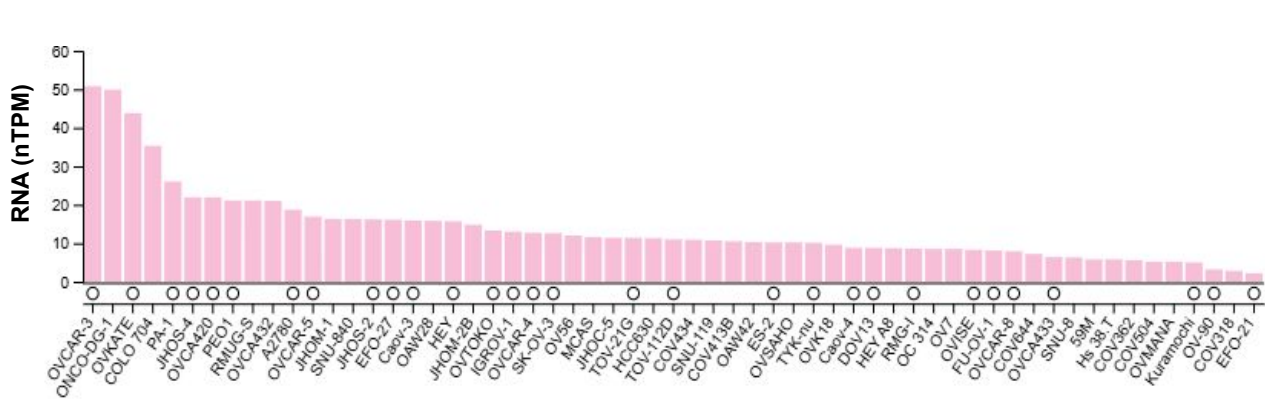

Supplement: Supplementary file 2 [file DataSheet2.pdf]

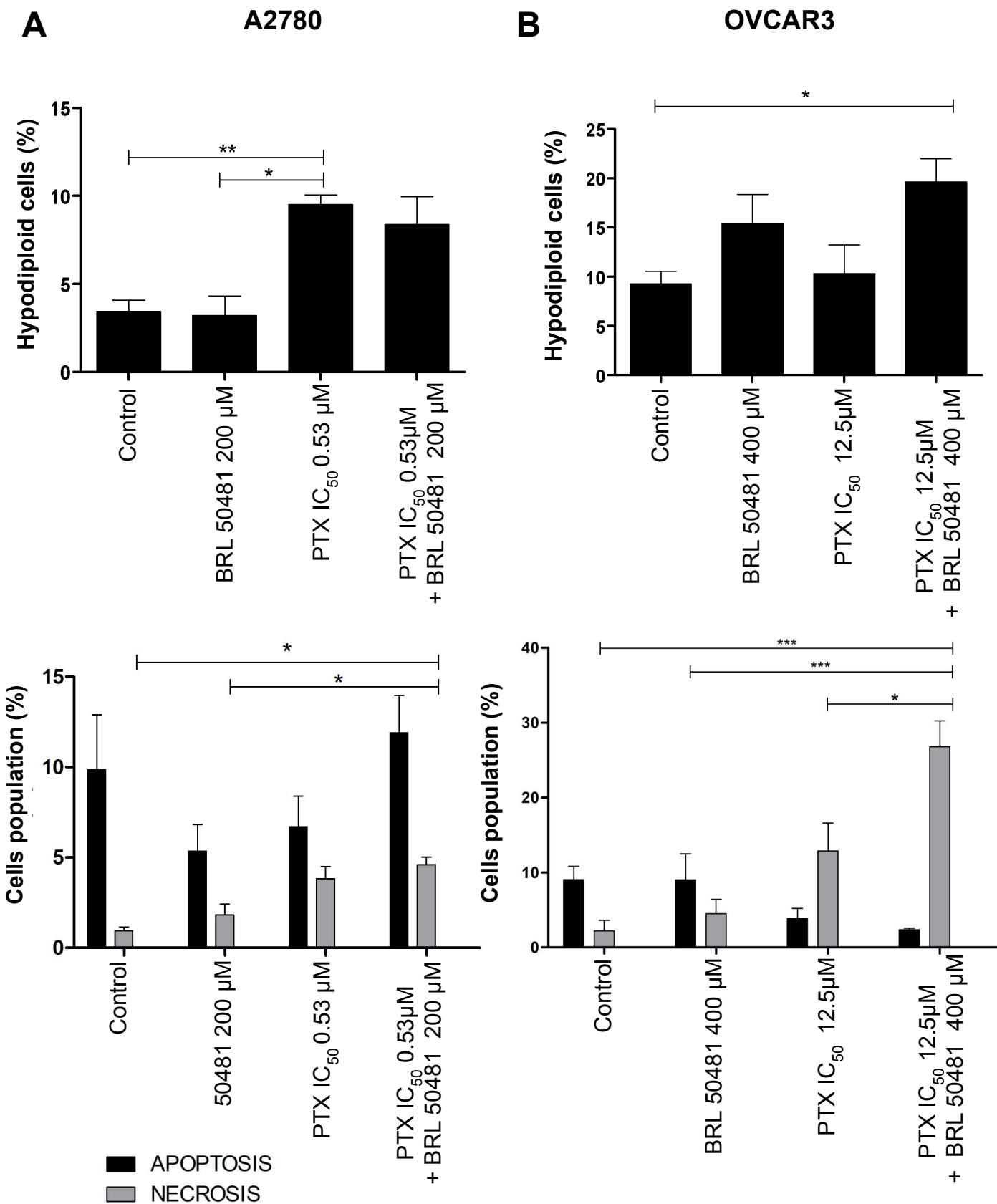

Supplement: Supplementary file 3 [file DataSheet4.pdf]

A A2780

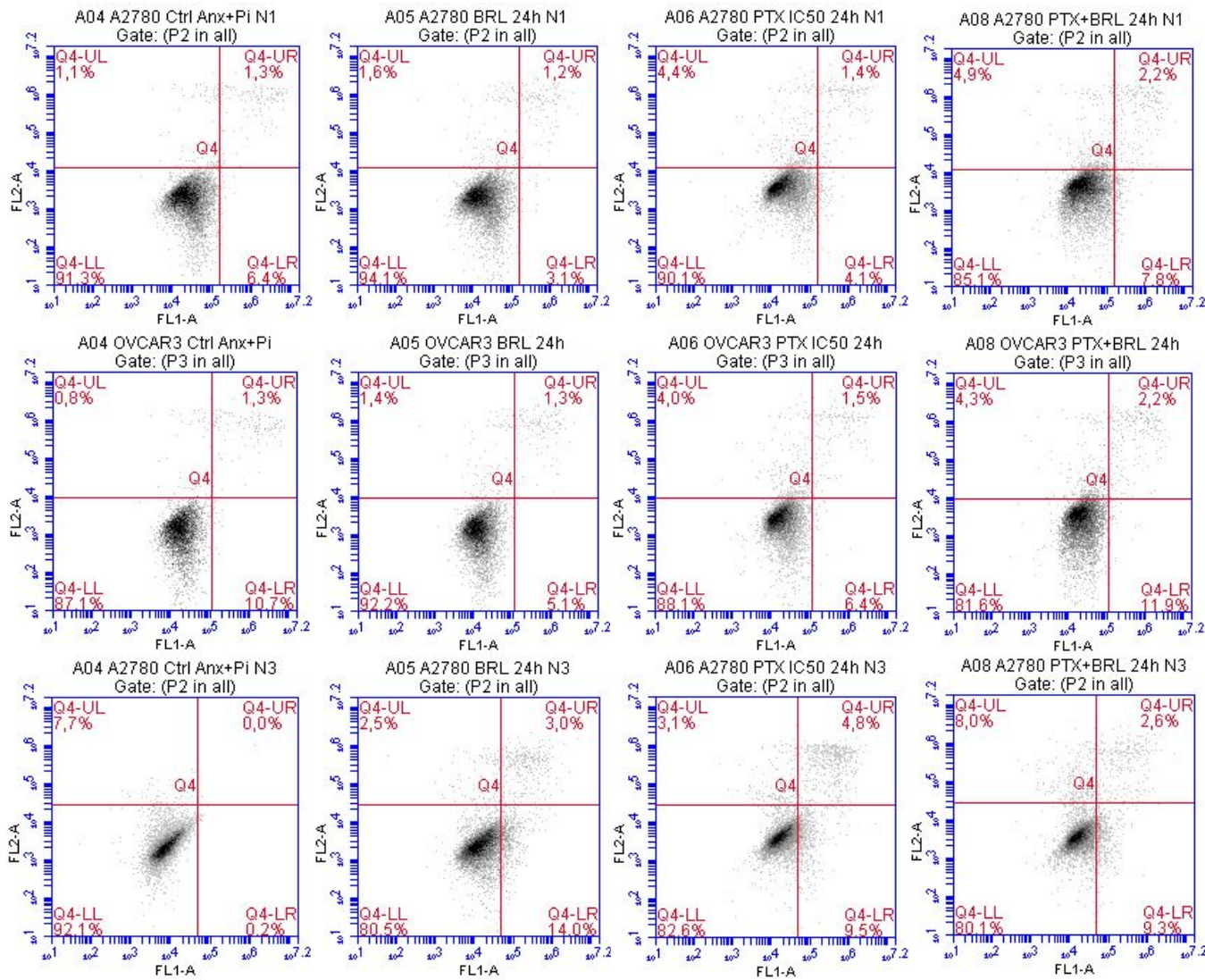

## B OVCAR3

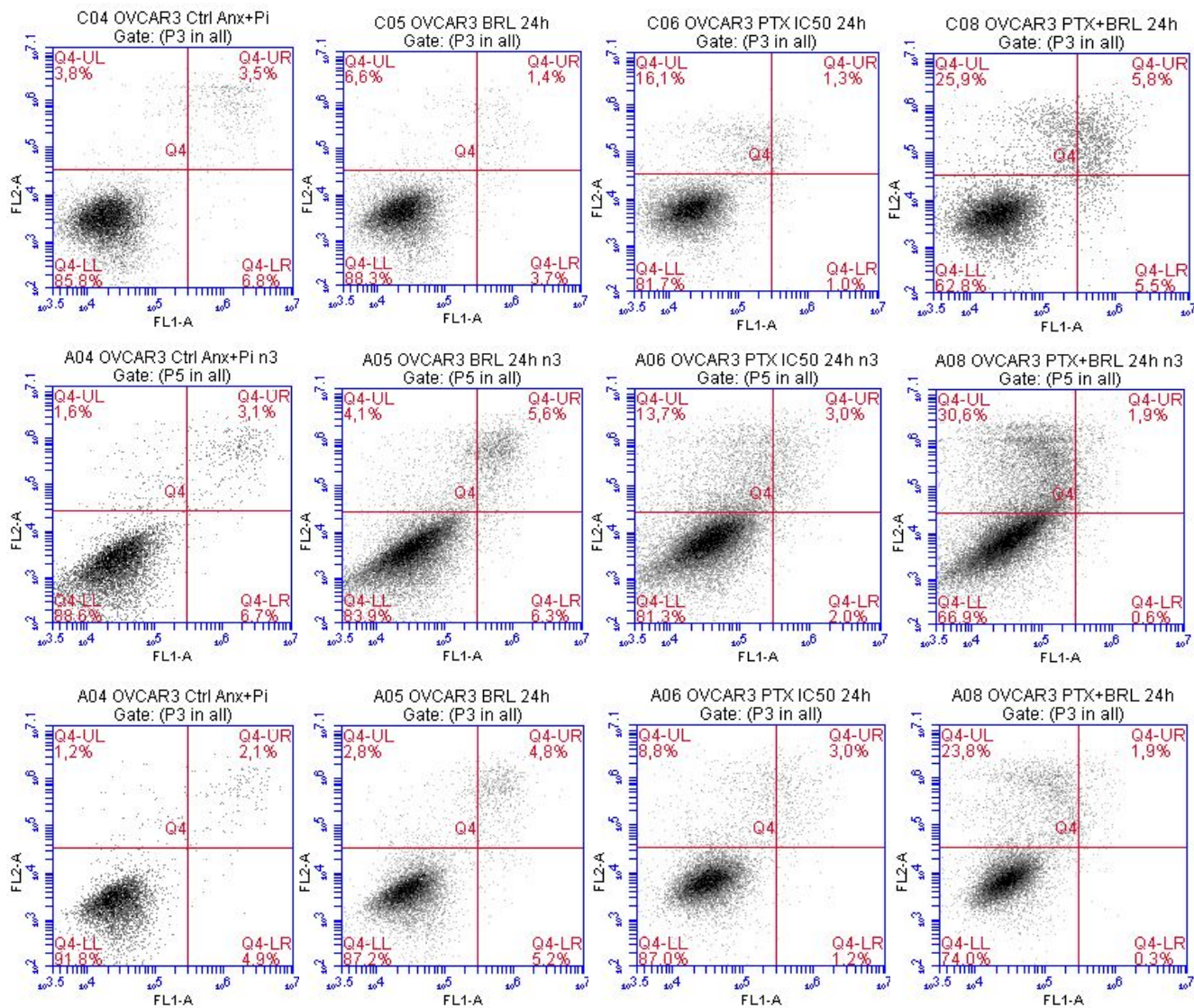

Supplement: Supplementary file 7 [file DataSheet5.pdf]
