# Supplementary material for: Phosphodiesterase 7: a potential novel therapeutic target in ovarian cancer
Source: Front Pharmacol. 2025 Jun 4;16:1566330. doi: 10.3389/fphar.2025.1566330 (PMC12174393; doi:10.3389/fphar.2025.1566330)

Blot PDE7A

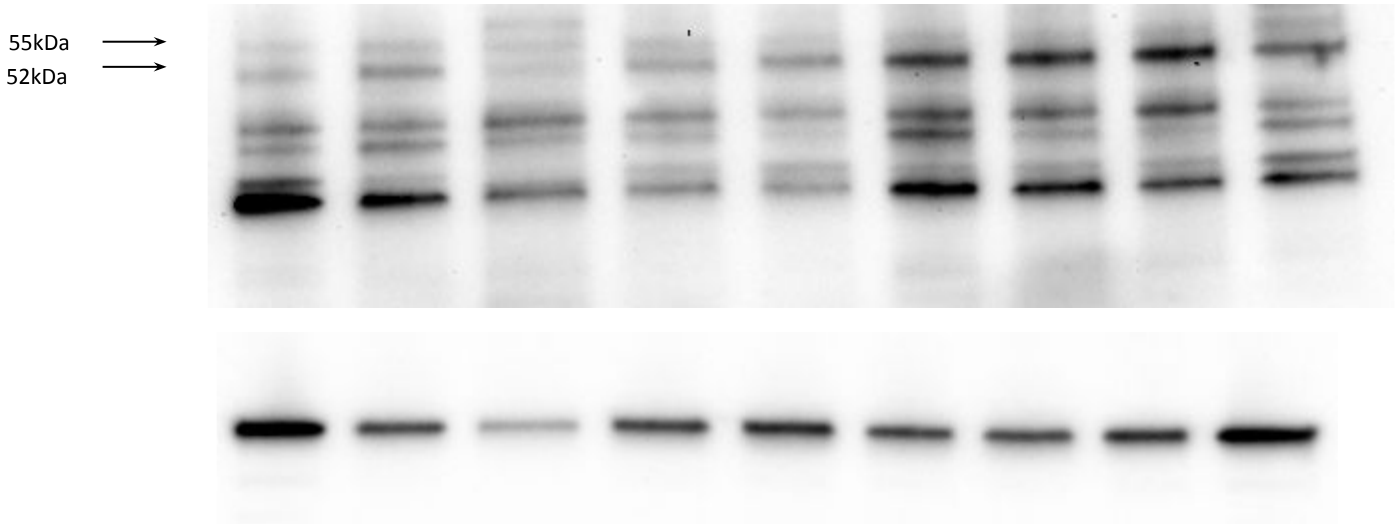

Blot AKT A2780 (n=1)

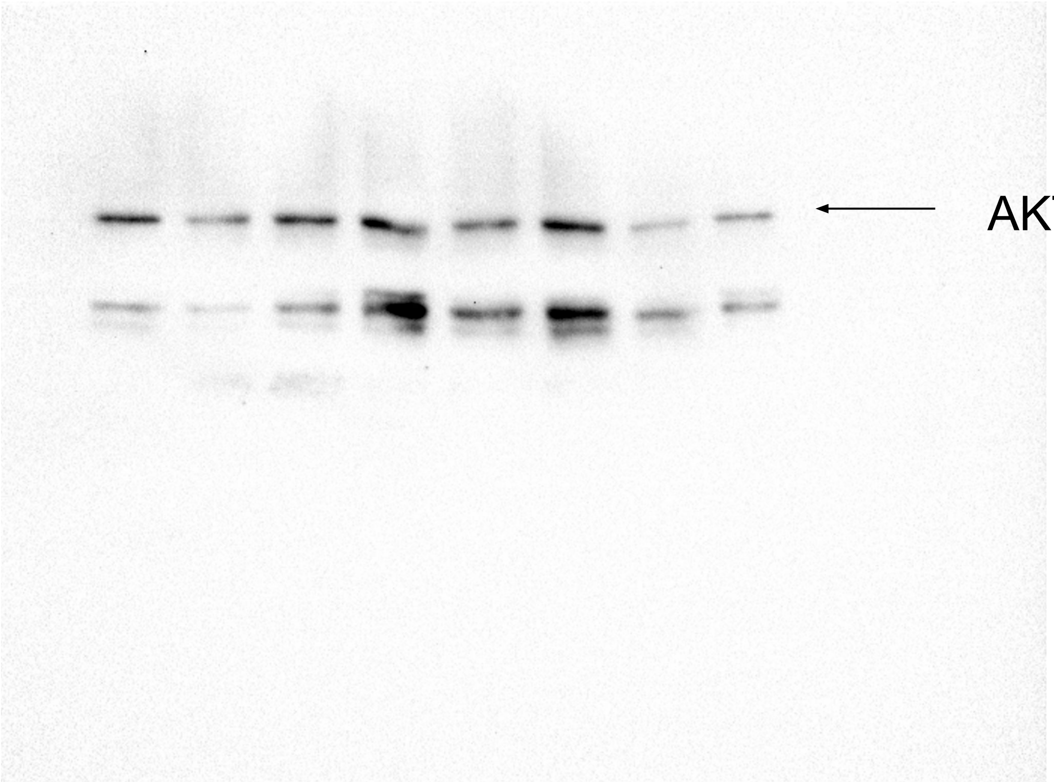

AKT

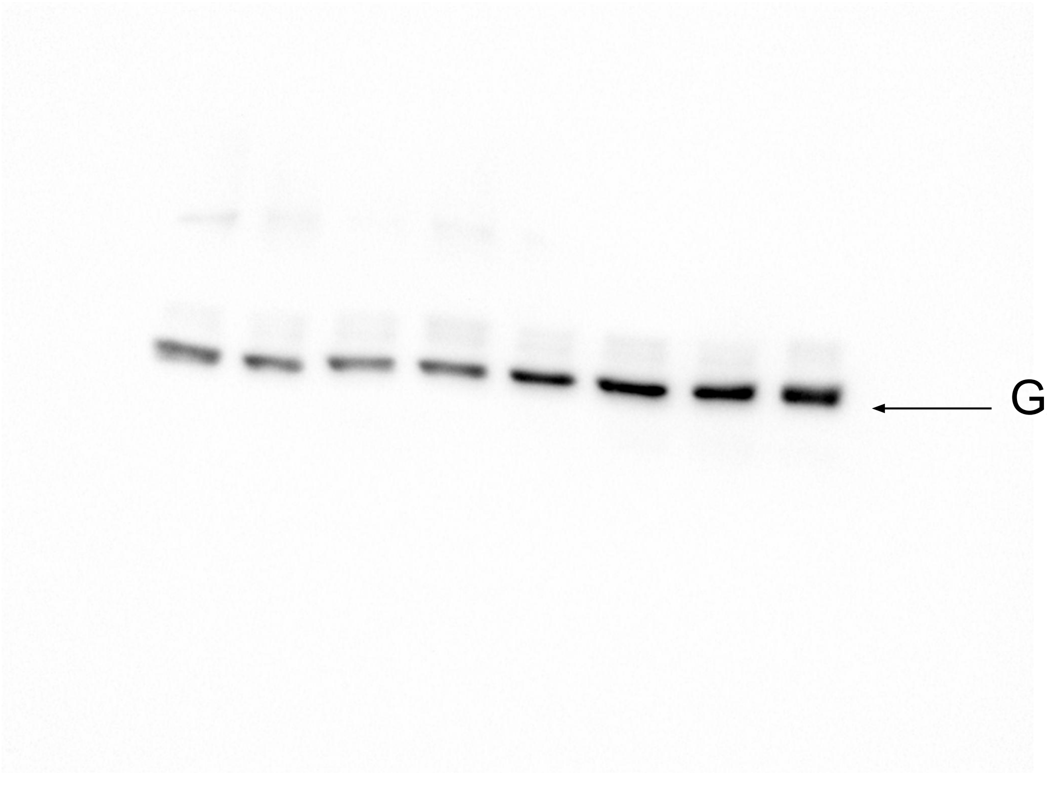

GAPDH

Blot AKT A2780 (n=2)

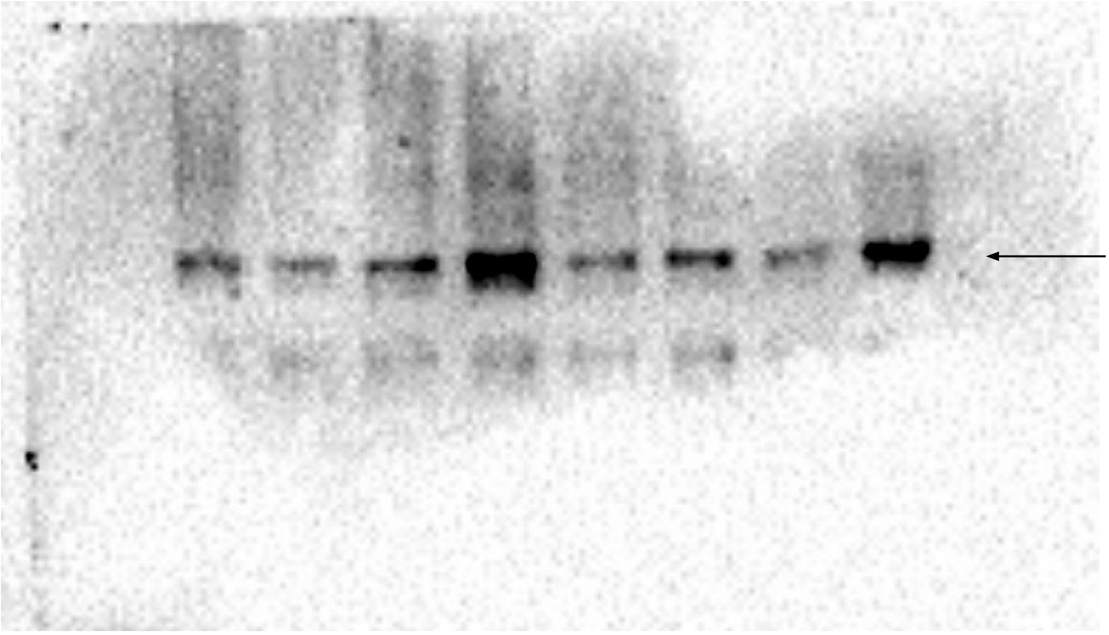

AKT

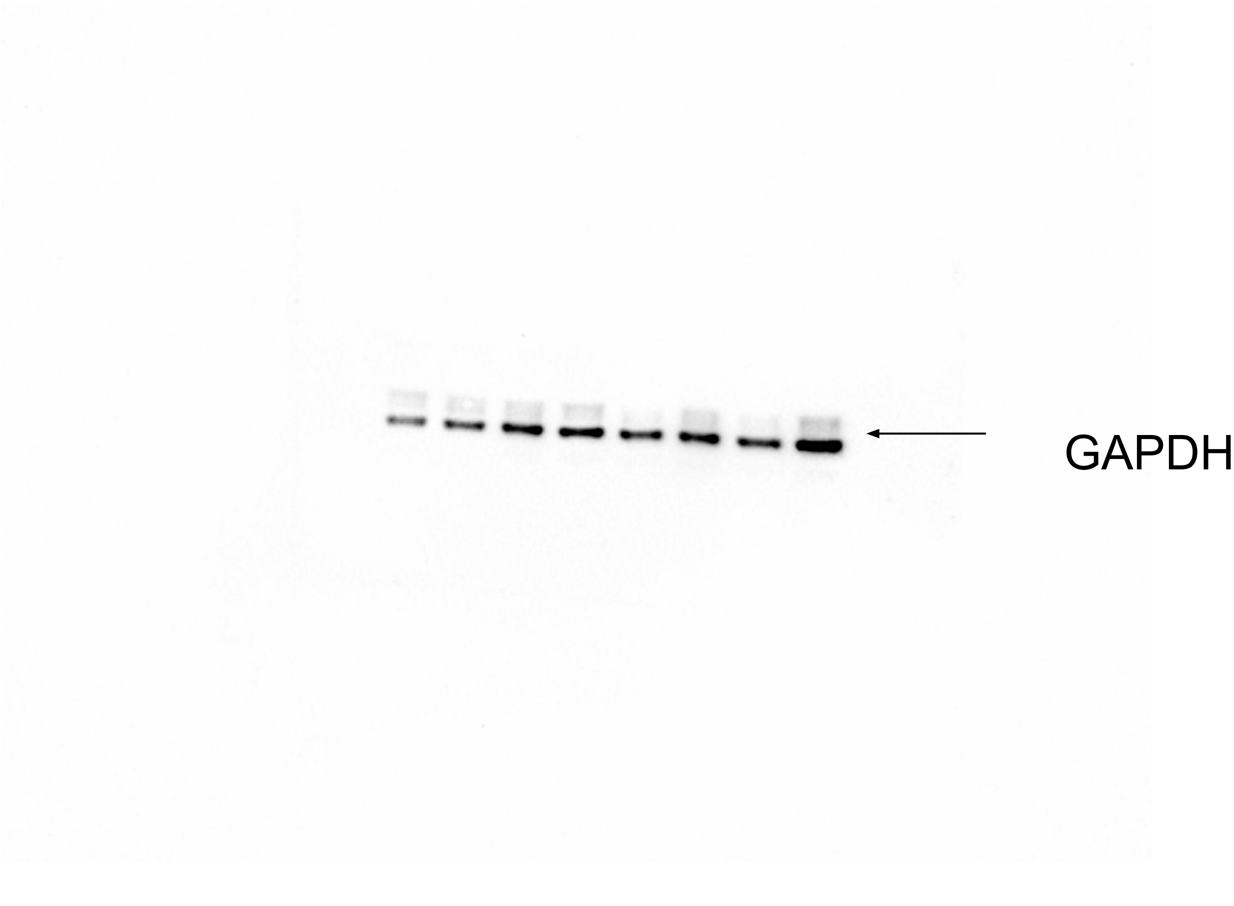

GAPDH

Blot AKT A2780 (n=3)

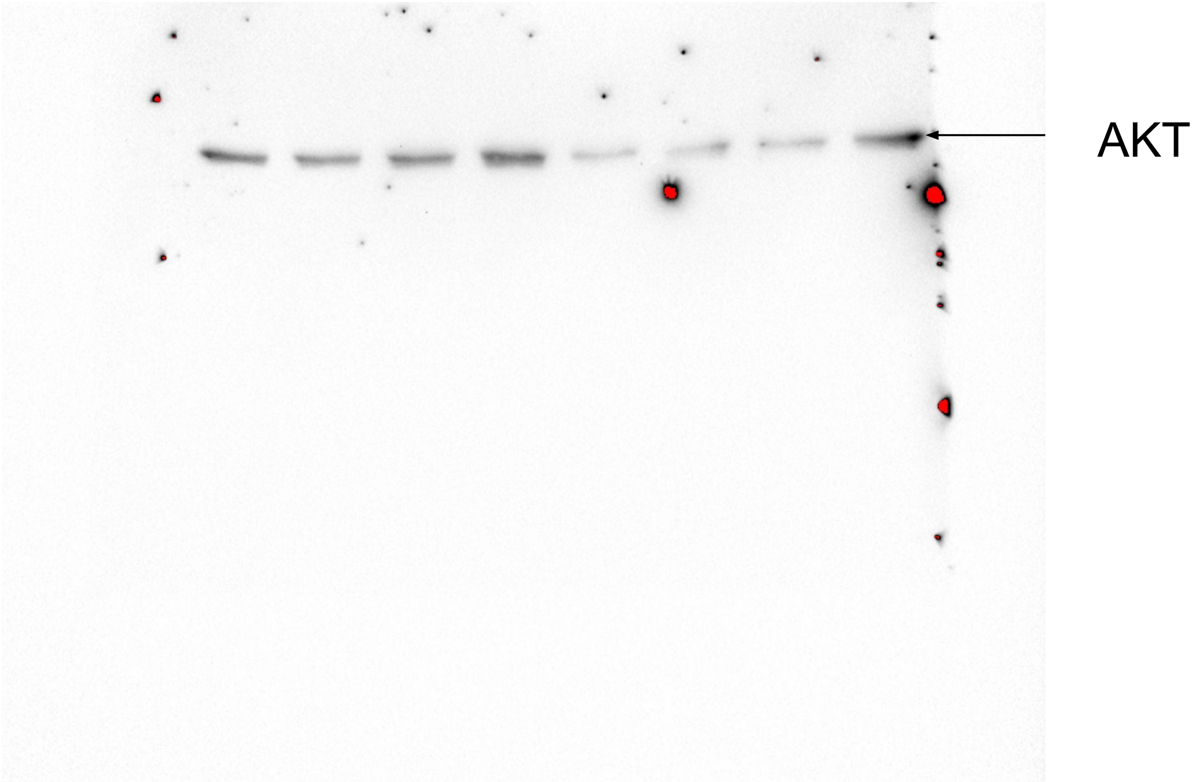

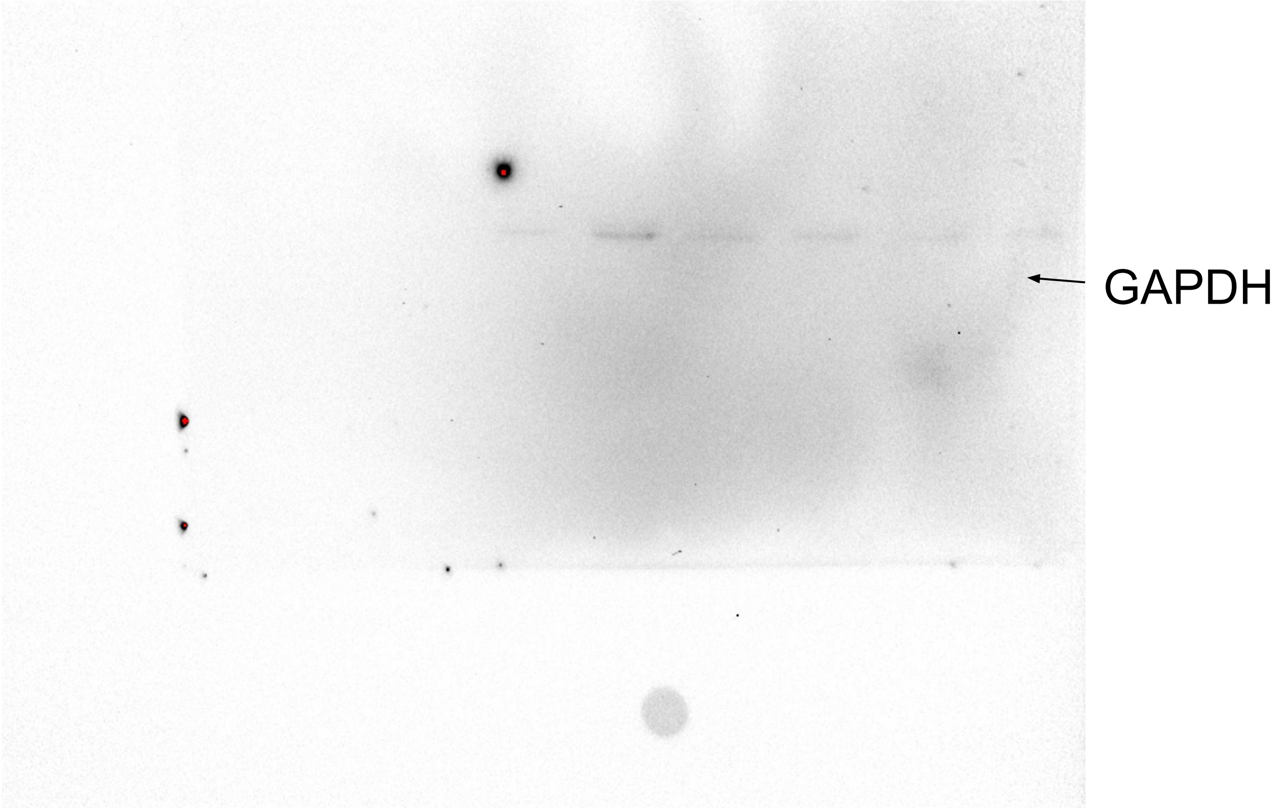

Blot AKT A2780 (n=4)

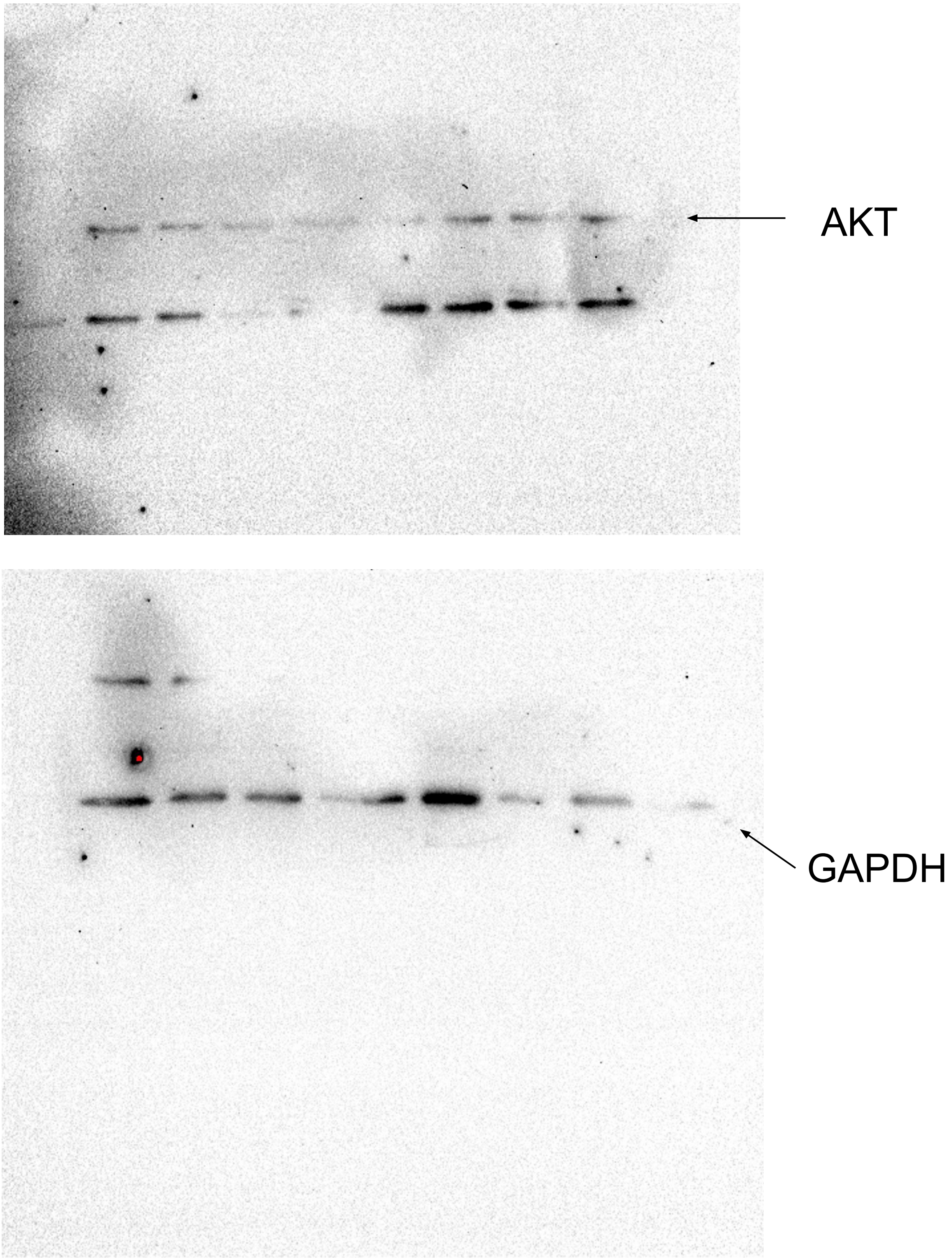

Blot pAKT A2780 (n=1)

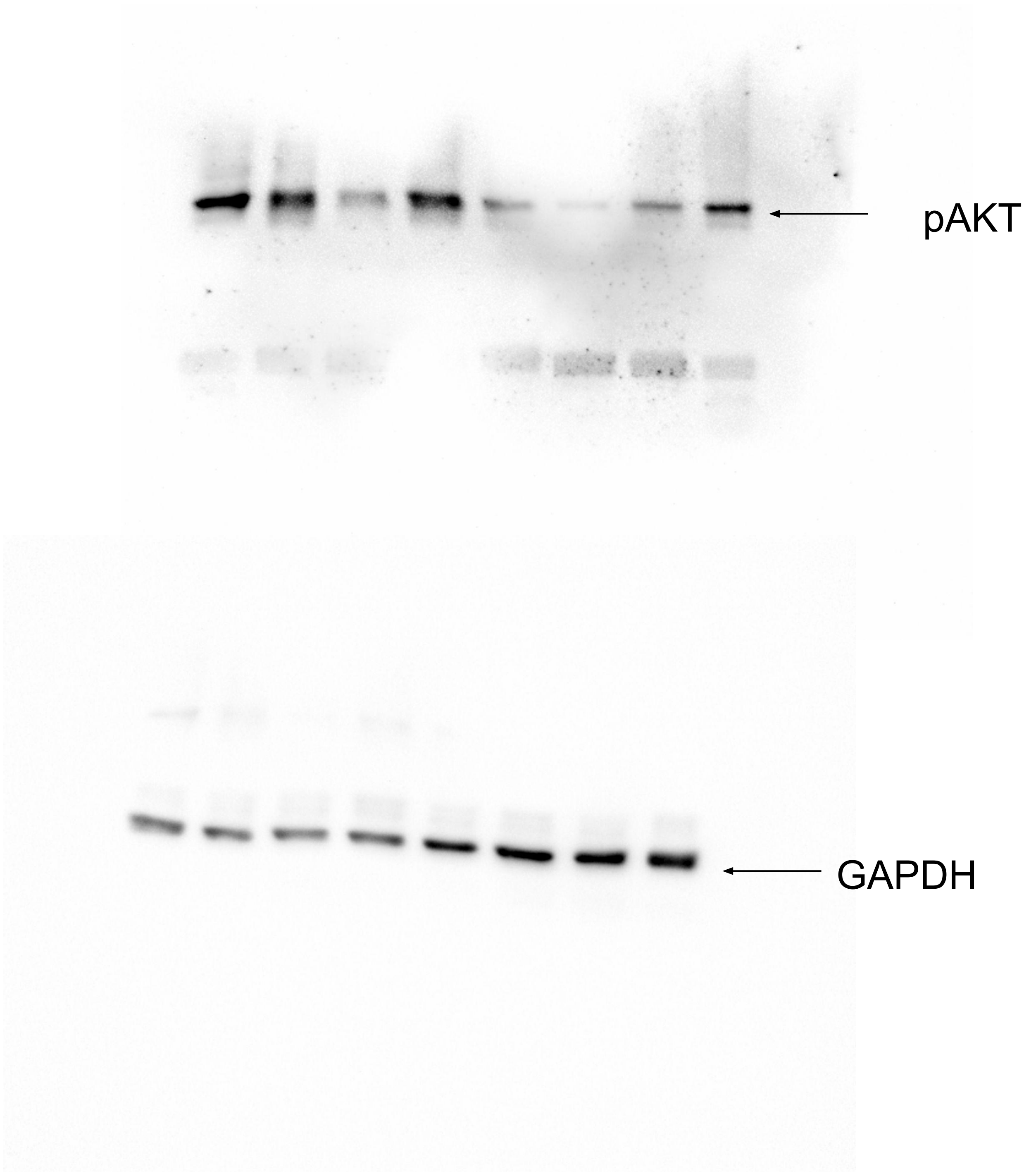

Blot pAKT A2780 (n=2)

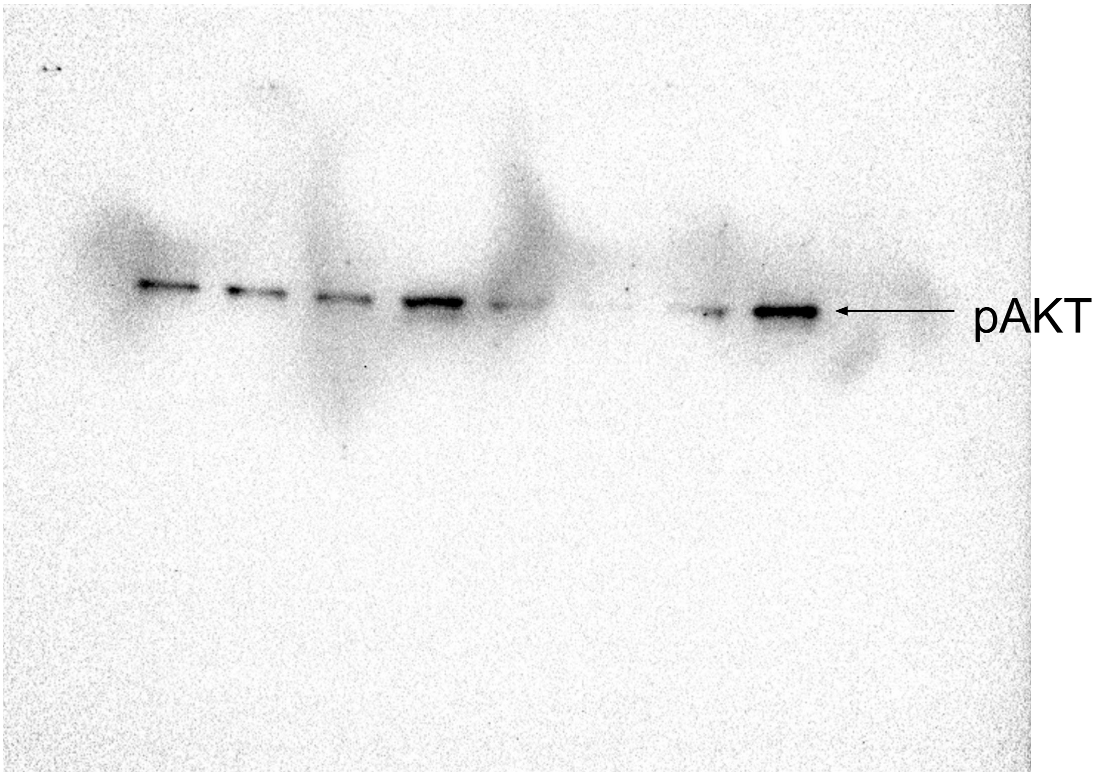

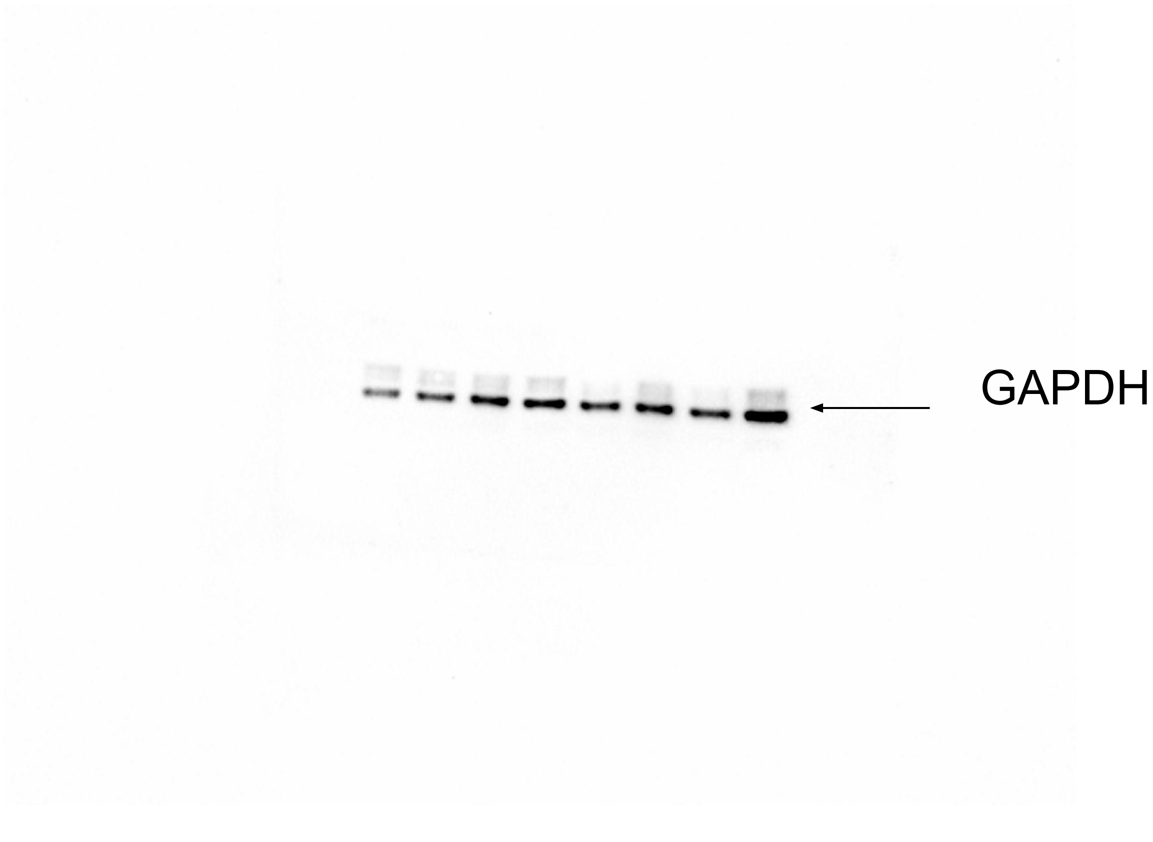

Blot pAKT A2780 (n=3)

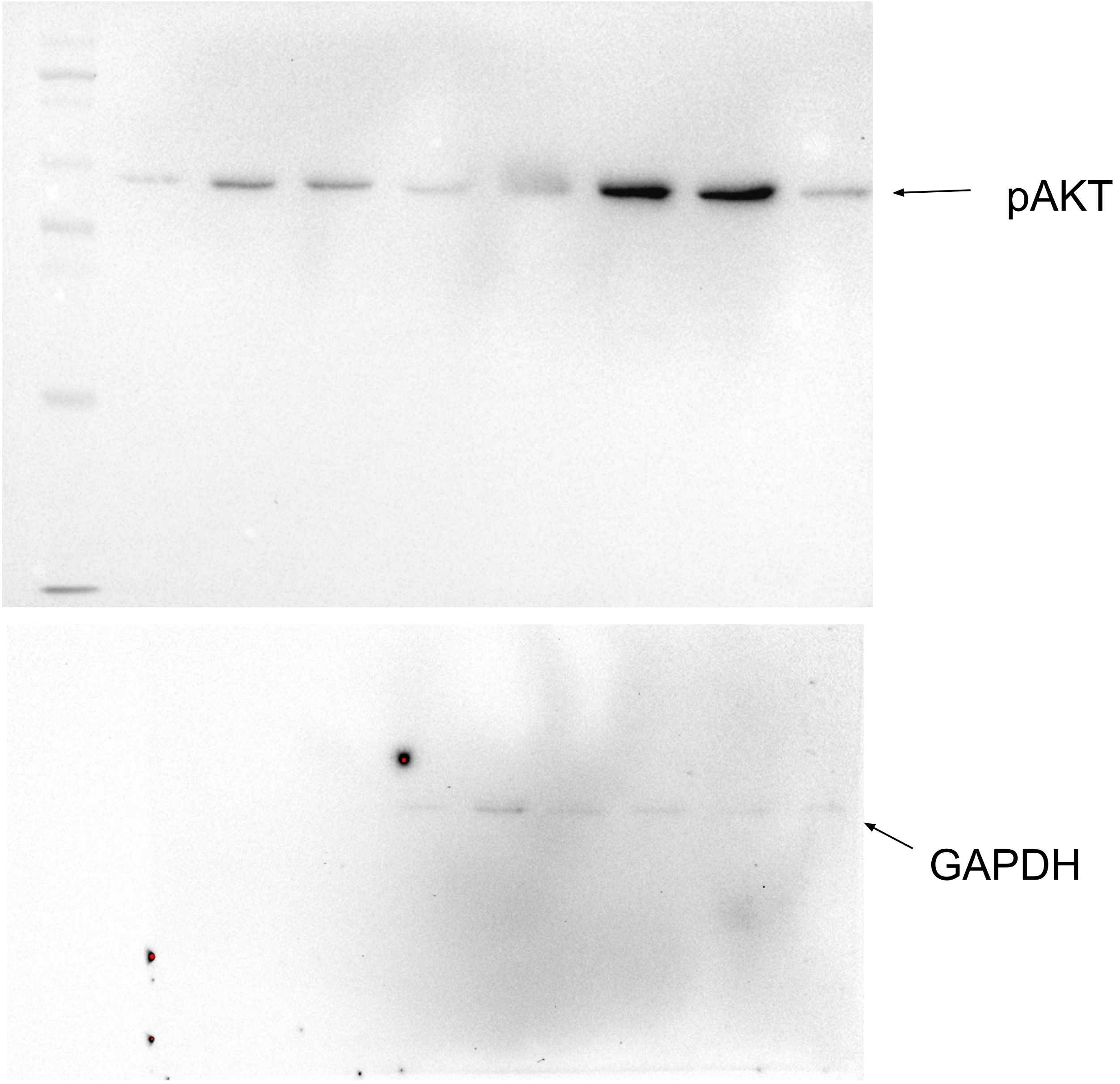

Blot pAKT A2780 (n=4)

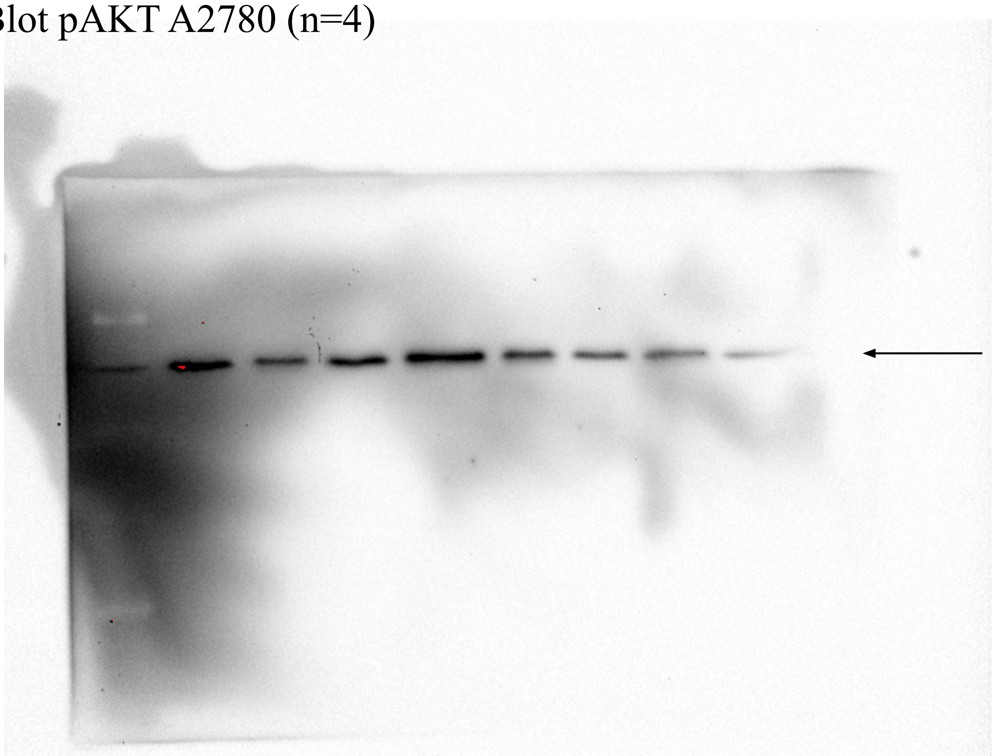

pAKT

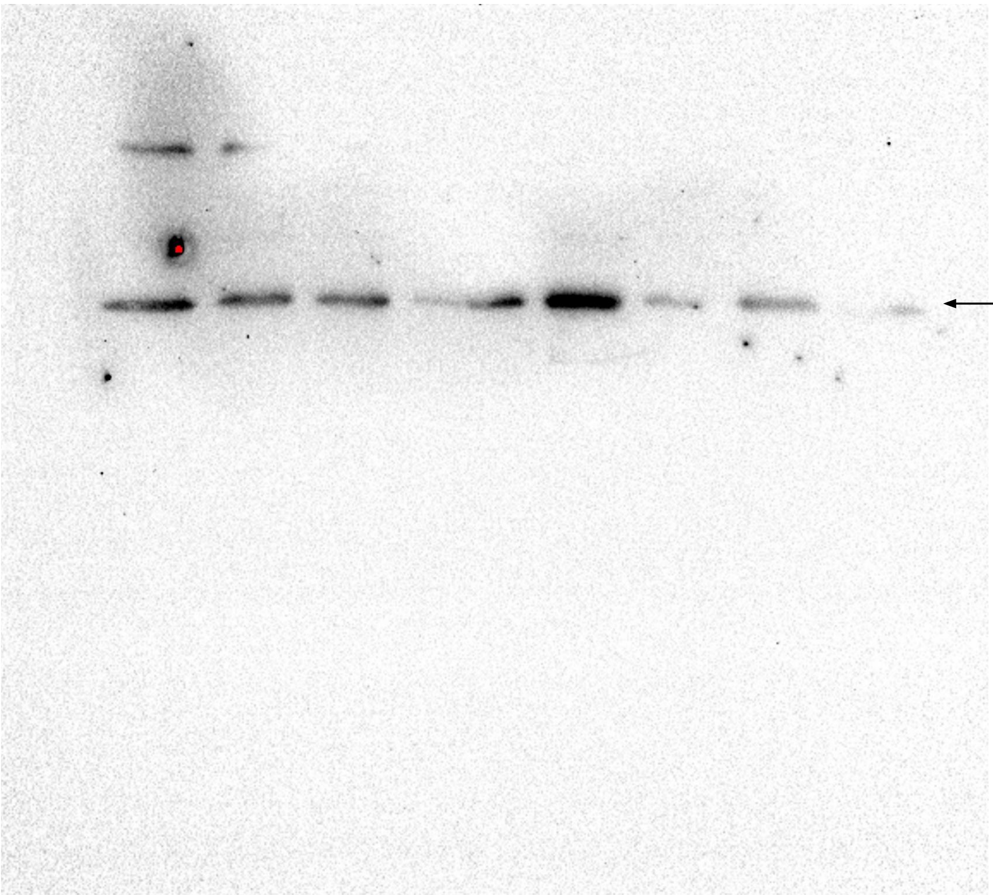

GAPDH

Blot BAX A2780 (n=1)

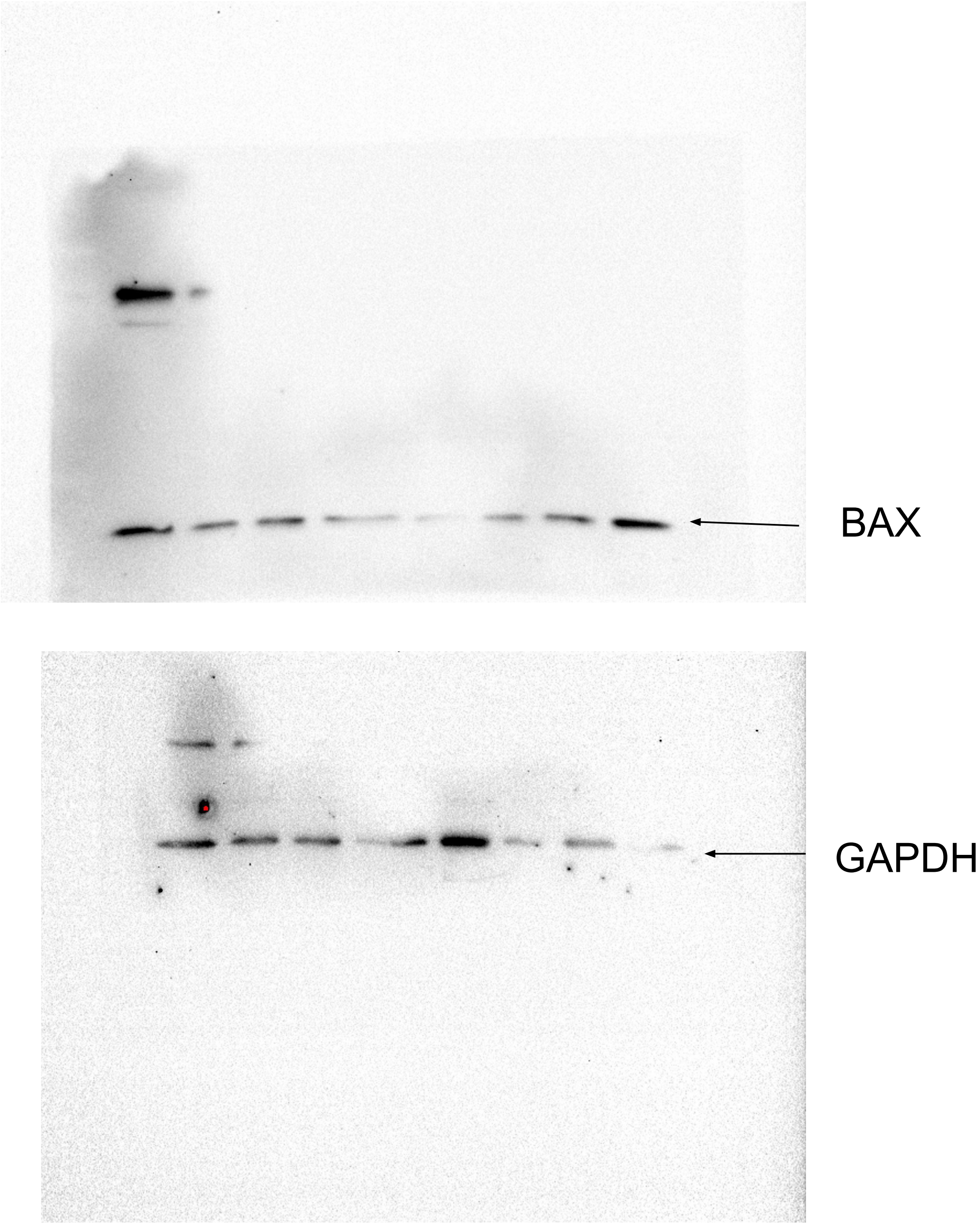

Blot BAX A2780 (n=2)

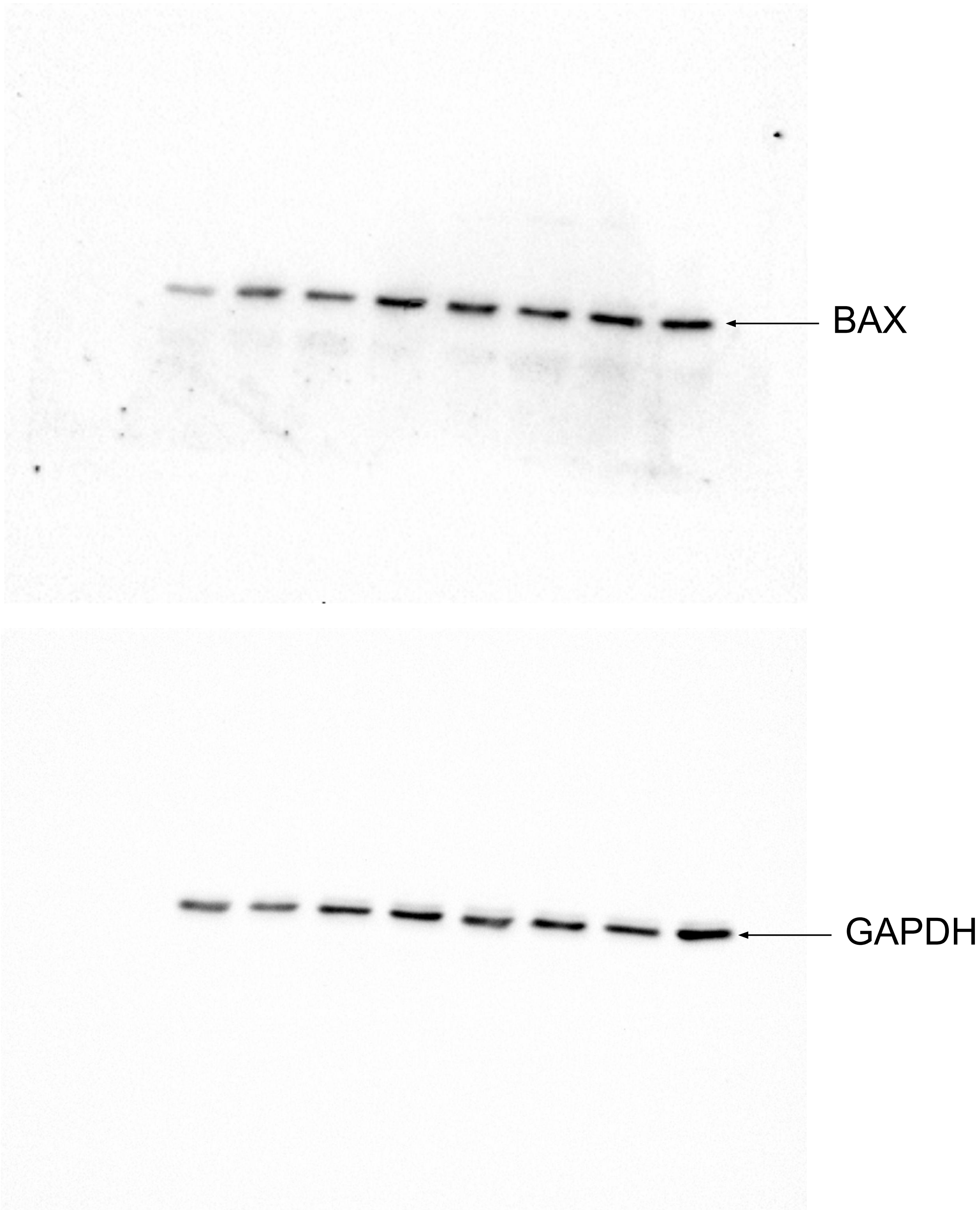

Blot BAX A2780 (n=3)

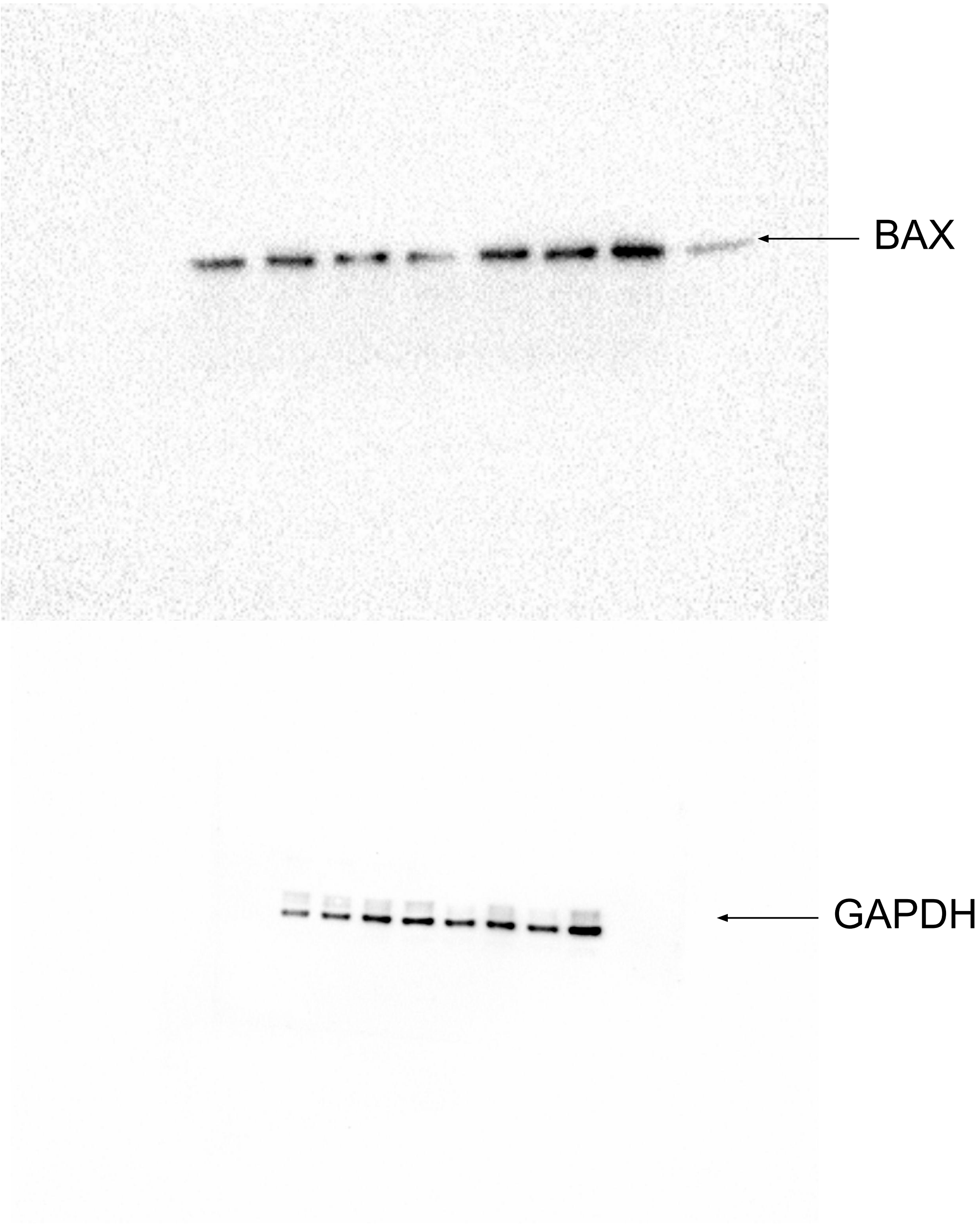

Blot AKT OVCAR3 (n=1)

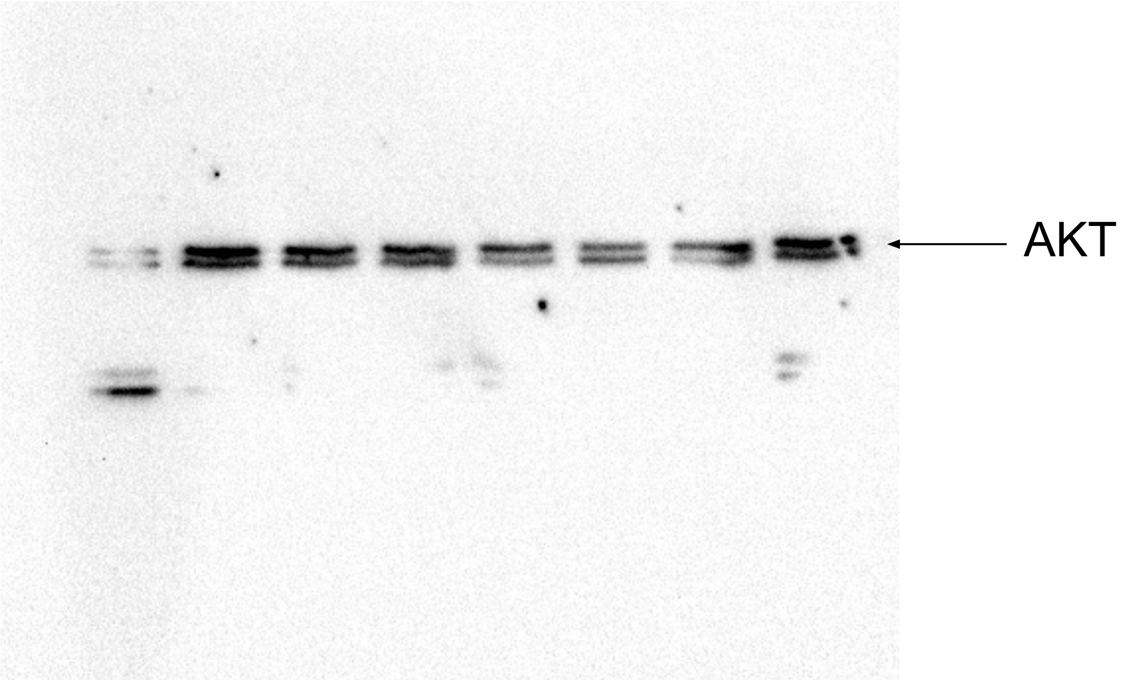

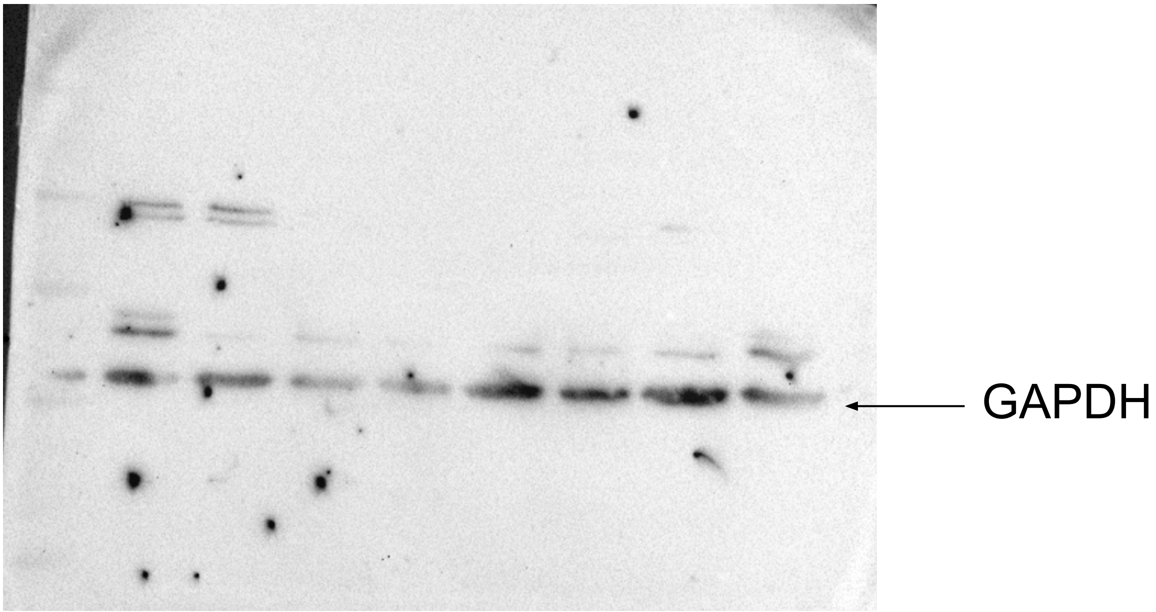

Blot AKT OVCAR3 (n=2)

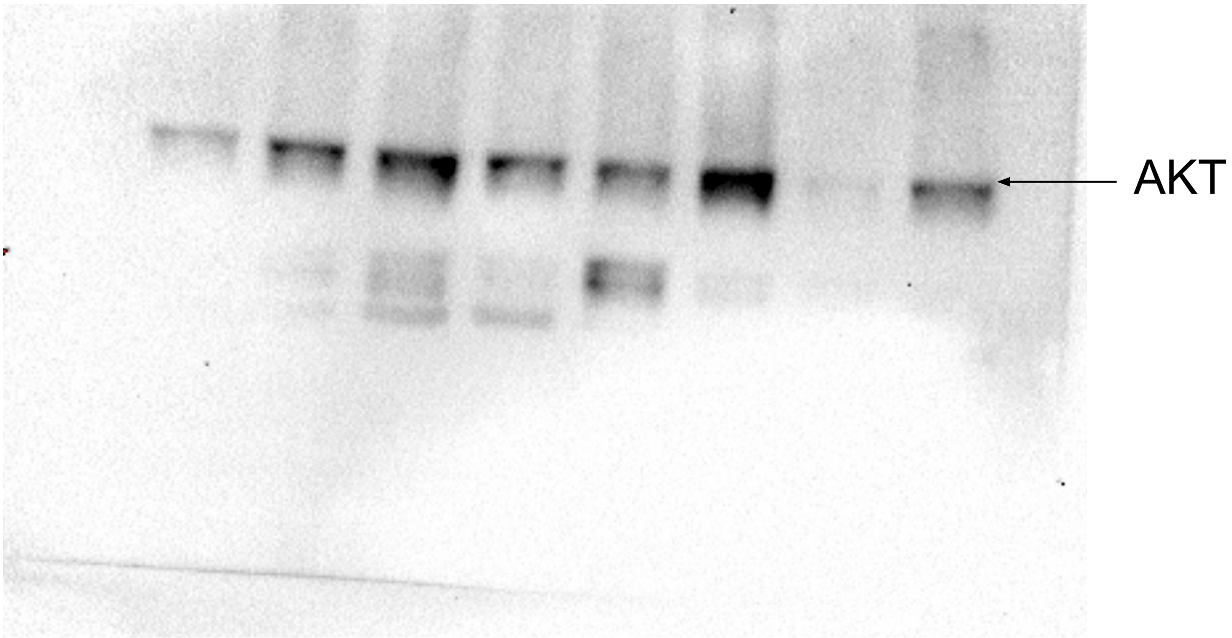

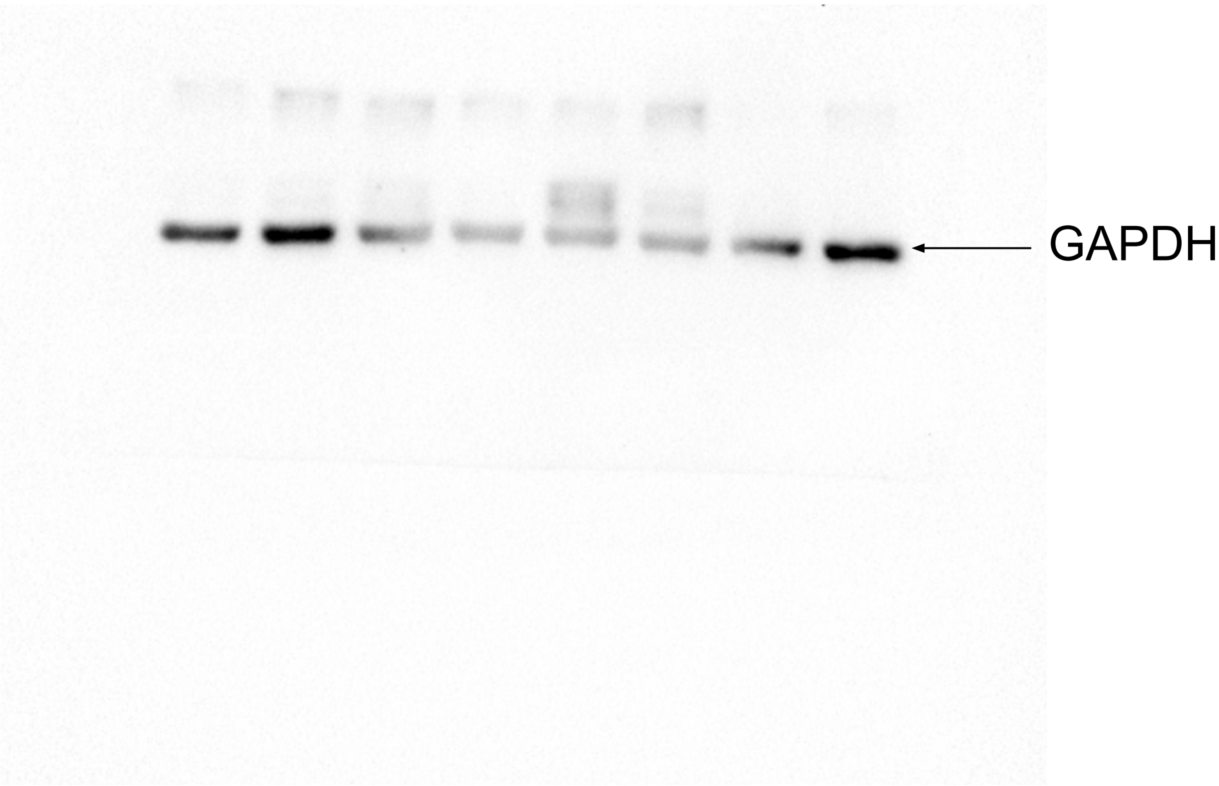

Blot AKT OVCAR3 (n=3)

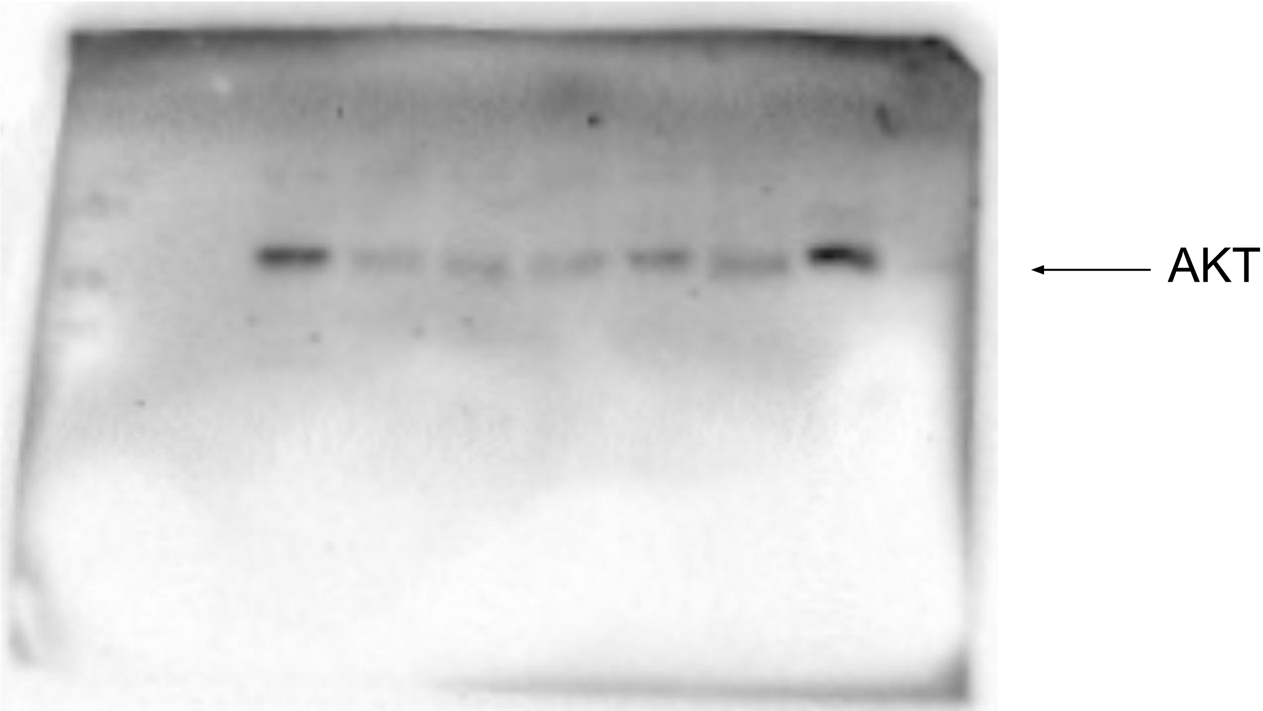

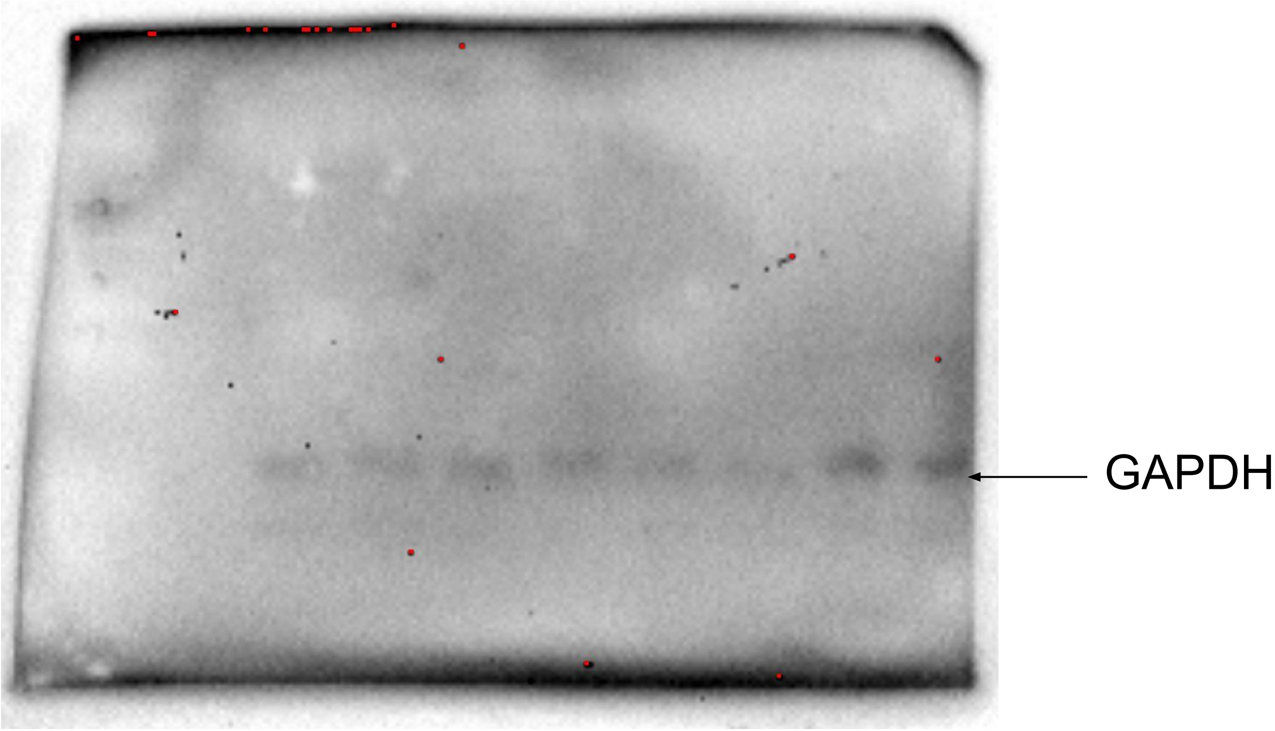

Blot pAKT OVCAR3 (n=1)

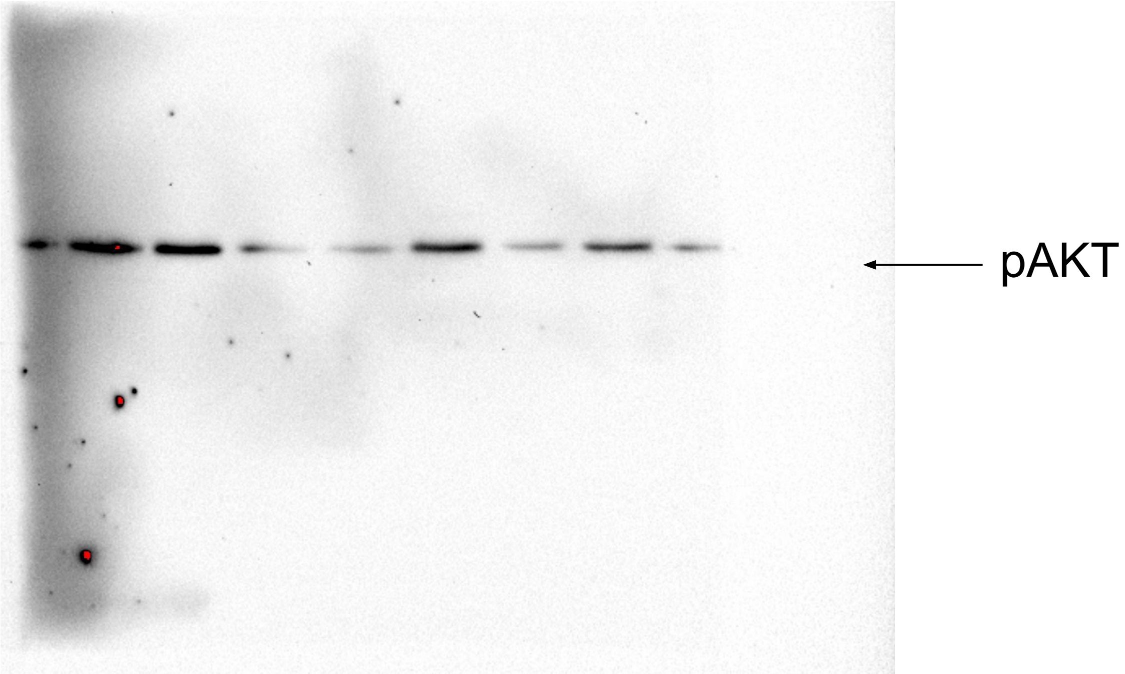

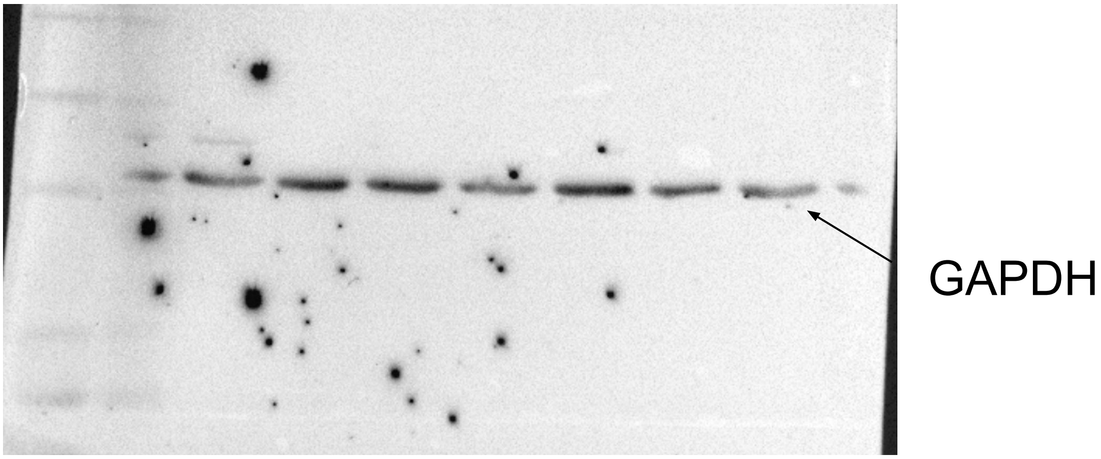

Blot pAKT OVCAR3 (n=2)

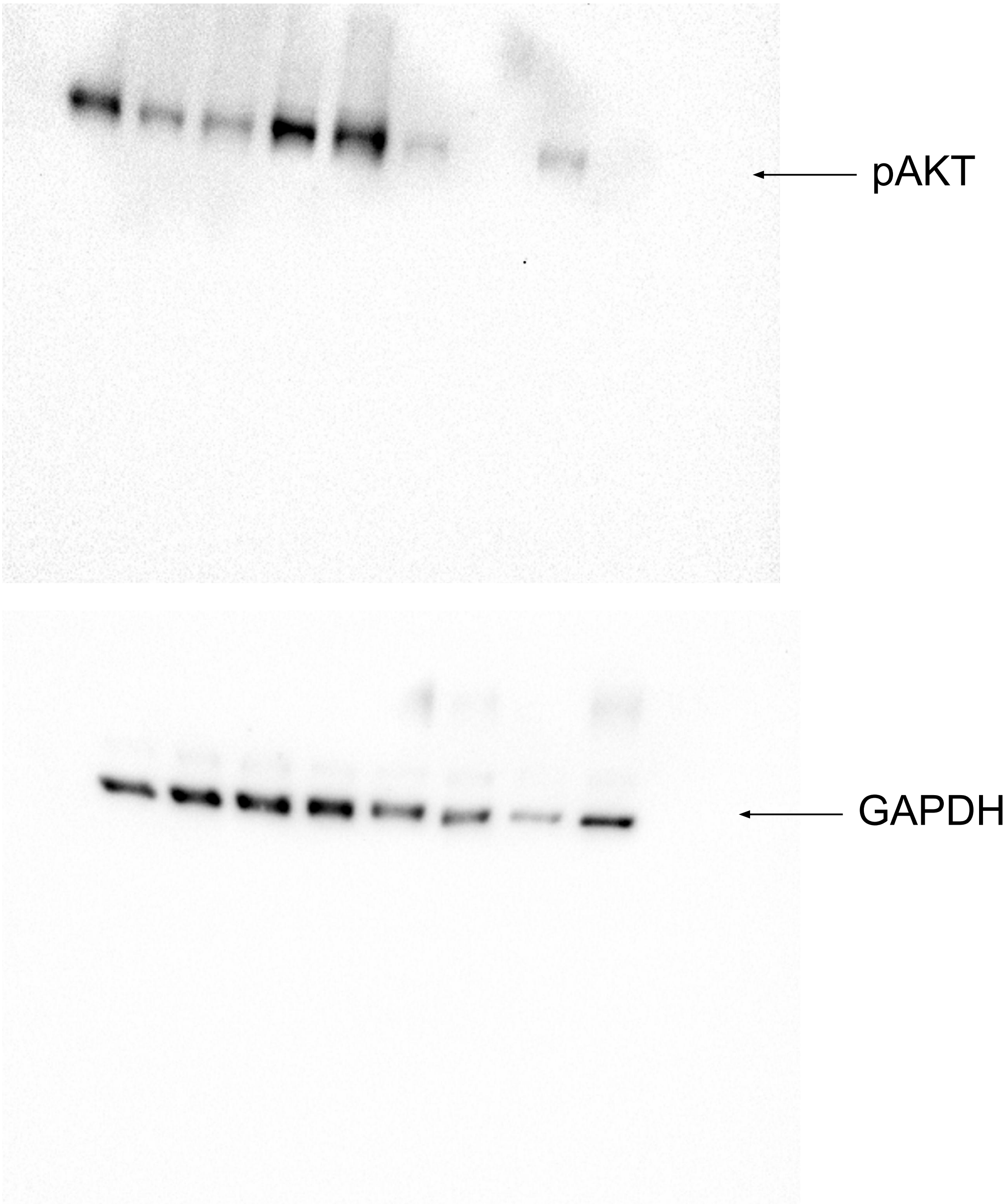

Blot pAKT OVCAR3 (n=3)

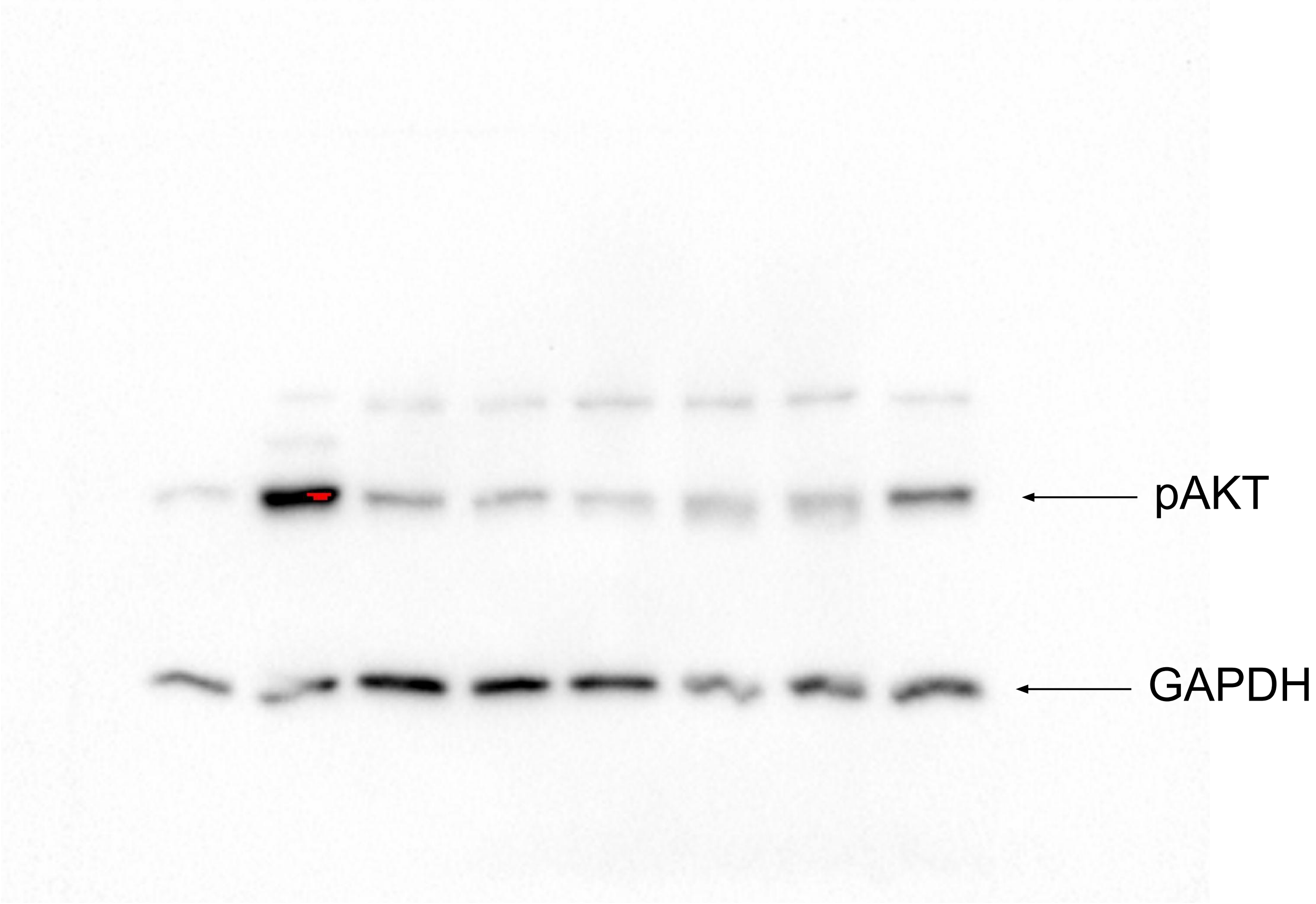

Supplement: Supplementary file 4 [file DataSheet6.pdf]
