# Supplementary material for: Phosphodiesterase 7: a potential novel therapeutic target in ovarian cancer
Source: Front Pharmacol. 2025 Jun 4;16:1566330. doi: 10.3389/fphar.2025.1566330 (PMC12174393; doi:10.3389/fphar.2025.1566330)
Supplement: Supplementary file 5 [file DataSheet3.pdf]

S3 Figure

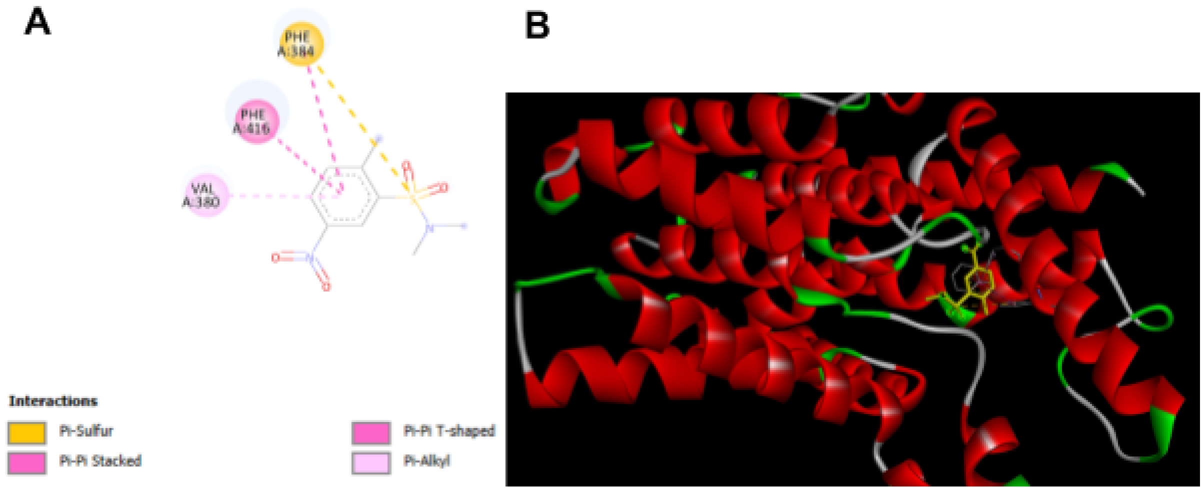

Supplementary Figure 3: Illustration of the molecular interactions between BRL50481 and the active site of PDE7A in both 2D (A) and 3D (B) representations. The sulfone group of BRL50481 forms a π-sulfur interaction with the Phe384 residue in the enzyme's active site. Additionally, the aromatic ring of BRL50481 interacts with Phe384, Phe416, and Val380 through π–π and π-alkyl interactions, contributing to its binding stability.
