# Supplementary material for: Phosphodiesterase 7: a potential novel therapeutic target in ovarian cancer
Source: Front Pharmacol. 2025 Jun 4;16:1566330. doi: 10.3389/fphar.2025.1566330 (PMC12174393; doi:10.3389/fphar.2025.1566330)
Supplement: Supplementary file 6 [file DataSheet1.pdf]

grcf at Johns Hopkins
DNA Services/FAF

Laura Kasch-Semenza
Johns Hopkins University
2760 Light House Point East, Suite 201
Baltimore, Maryland 21224
Phone: 410-614-3830
Email: lkasch@jhmi.edu

JOHNS HOPKINS
MEDICINE

STR Profile Report for Cell Line Authentication

Principal Investigator: Tian Li Wang
Institution: Johns Hopkins University
Cell Line ID: OVCAR5
Report Date: May 31, 2015
Submitting Researcher: Jin Jung
Sample Type Submitted: DNA
GeneSifter Tracking ID: 120058
Profile Kit Requested: GenePrint 10 (Promega)

STR Profile:

Loci	OVCAR5
AMEL	X
CSF1PO	10
D13S317	10,13
D16S539	11
D21S11	31
D5S818	11,13
D7S820	10
TH01	7,9.3
TPOX	8,11
vWA	16

STR Graphic Profile:

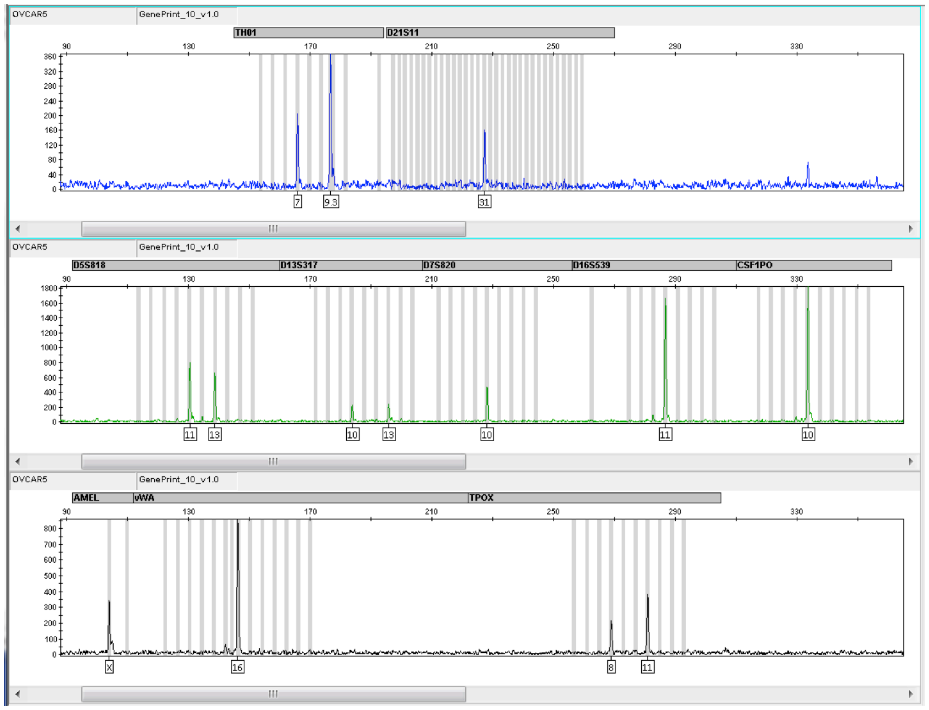

Peaks that appear to be legitimate stutter or are spectral bleed-through artifacts are not labeled.

e-Signature Director of Laboratory:

Laura Kasch

Digitally signed by Laura Kasch
DN: cn=Laura Kasch, o=Genetic Resources Core Facility, ou=Johns Hopkins University, email=lkasch@jhmi.edu, c=US
Date: 2015.05.31 20:21:27 -05'00'

FAF services are for research purposes only. Any data or samples generated by the facility may not be used for human diagnostic or therapeutic use. Results may not be returned to patients, placed into medical records, or be used to make clinical decisions.

grcf at Johns Hopkins
DNA Services/FAF

Laura Kasch-Semenza
Johns Hopkins University
2760 Light House Point East, Suite 201
Baltimore, Maryland 21224
Phone: 410-614-3830
Email: lkasch@jhmi.edu

JOHNS HOPKINS MEDICINE

Principal Investigator: Tian Li Wang
Institution: Johns Hopkins University
Cell Line ID: OVCAR5
Report Date: May 31, 2015

Procedure:
A Promega GenePrint 10 Kit was used to polymerase chain (PCR) amplify eight short tandem repeat (STR) loci plus a gender determining marker, Amelogenin. The PCR product was electrophoresed on an ABI Prism® 3730xl Genetic Analyzer using an Internal Lane Standard 600 (Promega). Data was analyzed using GeneMapper® v 4.0 software (Applied Biosystems). Appropriate positive and negative controls were used.

- Please see the Pdf file *Additional Information* sent with the results for percent match calculations and additional information about interpreting the STR profile results.

Raw Data Table:

Sample Name	Marker	Allele 1	Allele 2	Size 1	Size 2	Height 1	Height 2	Peak Area 1	Peak Area 2
OVCAR5	AMEL	X		103.92		343		2357	
OVCAR5	CSF1PO	10		333.57		1823		12366	
OVCAR5	D13S317	10	13	183.77	195.79	231	246	1379	1536
OVCAR5	D16S539	11		286.72		1662		10916	
OVCAR5	D21S11	31		227.28		162		1105	
OVCAR5	D5S818	11	13	130.4	138.68	796	668	4977	4099
OVCAR5	D7S820	10		228.1		469		2971	
OVCAR5	TH01	7	9.3	165.75	176.7	204	367	1317	2433
OVCAR5	TPOX	8	11	268.84	280.91	217	384	1766	2568
OVCAR5	vWA	16		146.2		860		5313	

FAF services are for research purposes only. Any data or samples generated by the facility may not be used for human diagnostic or therapeutic use. Results may not be returned to patients, placed into medical records, or be used to make clinical decisions.

grcf at Johns Hopkins DNA Services/FAF

Laura Kasch
Johns Hopkins University
2760 Light House Point East, Suite 201
Baltimore, MD 21224
Email: lkasch@jhmi.edu
https://grcf.jhmi.edu/

JOHNS HOPKINS MEDICINE

Cell Line Short Tandem Repeat (STR) Profile Report

Principal Investigator: Tian-Li Wang
Institution: Johns Hopkins University, Pthology
Cell Line ID: OVCAR8
Report Date: September 8, 2019
Submitting Researcher: Tian-Li Wang
Sample Type Submitted: Customer extracted DNA Cultured cells
GeneSifter Tracking ID: 159598
Profile Kit Requested: GenePrint 10 (Promega)

STR Profile:

Loci	OVCAR8
AMEL	X
CSF1PO	11
D13S317	12
D16S539	13
D21S11	28
D5S818	12
D7S820	12
TH01	7
TPOX	8
vWA	16,17

» Loci with one allele listed are homozygous for that STR marker.

e-Signature Director of Laboratory:

Laura Kasch

Digitally signed by Laura Kasch
DN: cn=Laura Kasch, o=Genetic Resources Core Facility, ou=Johns Hopkins University, email=lkasch@jhmi.edu, c=US
Date: 2019.09.08 20:04:23 -05'00'

STR Graphic Profile:

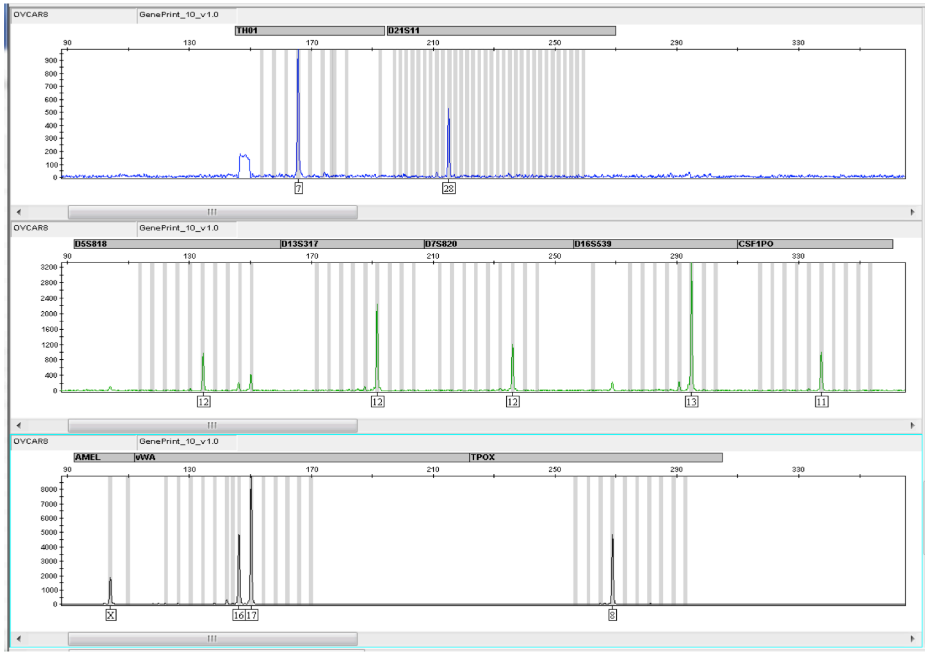

Peaks that appear to be legitimate stutter or are spectral bleed-through artifacts are not labeled.

- This report provides a STR profile for the cell line submitted, it does not automatically authenticate the cell line. Authentication is determined by comparison to the STR profile of the original donor tissue or cell stock. This STR profile can be used to verify the cell line is human, evaluate profile consistency between provisionally related cell isolates, detect cross-contamination with another human cell line (intra-species contamination), and to compare to profile databases, which we strongly recommend. This STR report does not rule out cross-contamination with an inter-species cell line (non-human).
- Please see the Pdf file *Additional Information* sent with the results for more information about interpreting your STR profile and links to profile databases.

GRCF DNA Services are for research purposes only. Any data or samples generated by the facility may not be used for human diagnostic or therapeutic use. Results may not be returned to patients, placed into medical records, or be used to make clinical decisions.

grcf at Johns Hopkins DNA Services/FAF

Laura Kasch
Johns Hopkins University
2760 Light House Point East, Suite 201
Baltimore, MD 21224
Email: lkasch@jhmi.edu
https://grcf.jhmi.edu/

JOHNS HOPKINS MEDICINE

Principal Investigator: Tian-Li Wang
Institution: Johns Hopkins University
Cell Line ID: OVCAR8
Report Date: September 8, 2019

Procedure:

A Promega GenePrint 10 Kit is used to PCR amplify eight short tandem repeat (STR) loci plus a gender determining marker, Amelogenin. The PCR product is electrophoresed on an ABI Prism® 3730xl Genetic Analyzer using an ILS600 internal lane standard (Promega). Data is analyzed using GeneMapper® v 4.0 software (Applied Biosystems). An allelic ladder that contains fragments of the same lengths as many of the known alleles for the loci is used to determine the amplified sample fragments alleles. Appropriate positive and negative controls are used.

Raw Data Table:

Sample Name	Marker	Allele 1	Allele 2	Size 1	Size 2	Height 1	Height 2	Peak Area 1	Peak Area 2
OVCAR8	AMEL	X	X	104.03	104.03	1865	1865	11473	11473
OVCAR8	CSF1PO	11	11	337.31	337.31	1001	1001	7200	7200
OVCAR8	D13S317	12	12	191.58	191.58	2239	2239	13745	13745
OVCAR8	D16S539	13	13	294.76	294.76	3318	3318	22705	22705
OVCAR8	D21S11	28	28	215.07	215.07	528	528	3227	3227
OVCAR8	D5S818	12	12	134.48	134.48	973	973	5940	5940
OVCAR8	D7S820	12	12	236.02	236.02	1216	1216	8096	8096
OVCAR8	TH01	7	7	165.67	165.67	985	985	6196	6196
OVCAR8	TPOX	8	8	268.87	268.87	4843	4843	31597	31597
OVCAR8	vWA	16	17	146.2	150.28	4851	8949	31140	56763

GRCF DNA Services are for research purposes only. Any data or samples generated by the facility may not be used for human diagnostic or therapeutic use. Results may not be returned to patients, placed into medical records, or be used to make clinical decisions.

grcf at Johns Hopkins DNA Services

Genetic Resources Core Facility
Johns Hopkins University
2760 Light House Point East, Suite 201
Baltimore, Maryland 21224

JOHNS HOPKINS MEDICINE

Additional Information

Electropherogram Interpretation

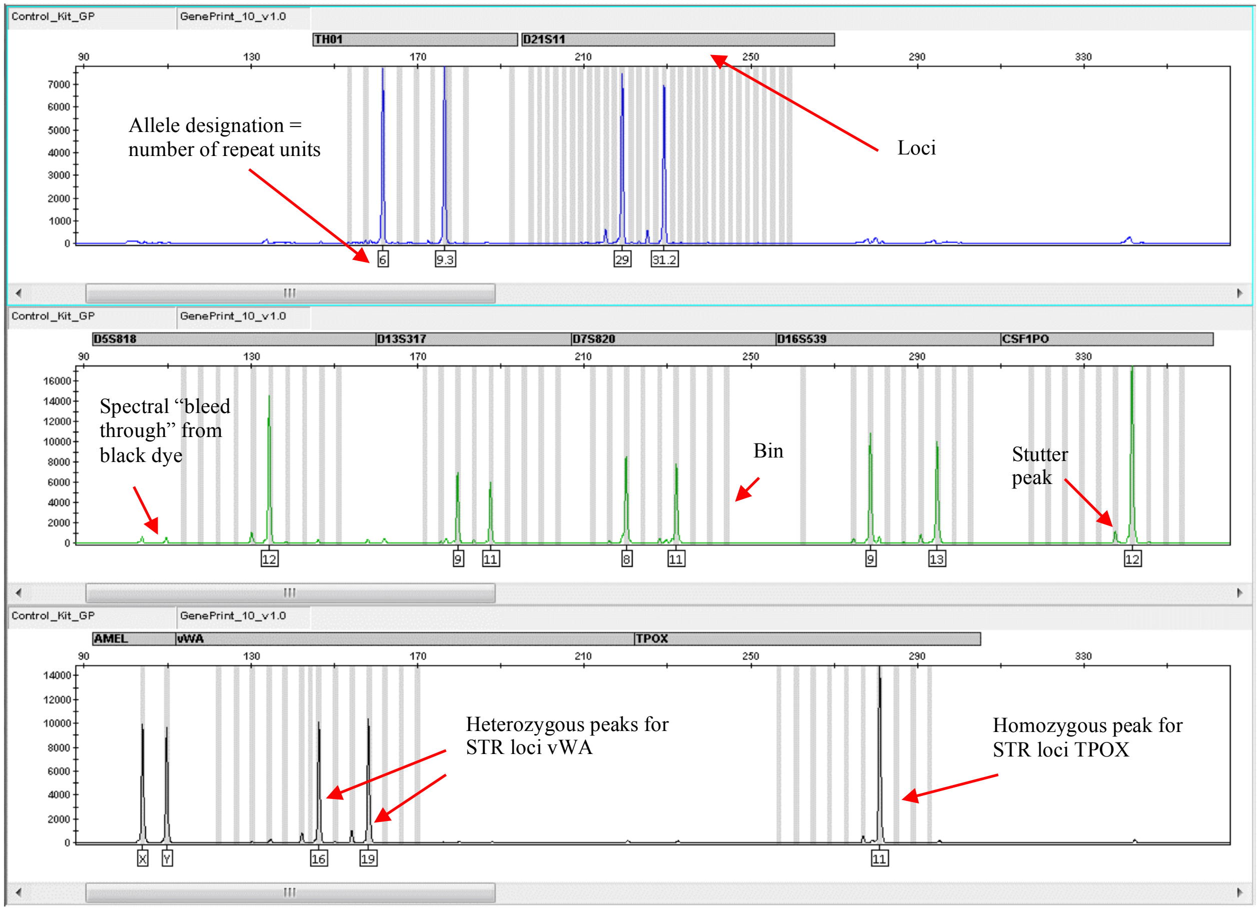

Stutter peaks can appear immediately before or after a true allele peak and are caused by the forward or backward slippage of the DNA polymerase during PCR amplification of a DNA strand. The designation of a stutter peak is based on the relative height of the stutter to the allele peak. This results in fragments of one repeat less or greater than the true allele.

- Some cell lines can be genetically unstable (loss of heterozygosity, allele duplication, genetic drift) and, therefore, STR profiles may vary from the parental line with the number of passages. In some cell lines it is common to see peak height imbalances and the presence of three (and sometimes more) alleles for a loci. If there are more than two peaks at several loci, a mixture of cell lines may be present. If your cell line is misidentified or appears to be contaminated, we suggest testing an earlier passage or, if possible, obtaining a new sample from a repository.

(Continued on next page)

grcf at Johns Hopkins
DNA Services

Genetic Resources Core Facility
Johns Hopkins University
2760 Light House Point East, Suite 201
Baltimore, Maryland 21224

JOHNS HOPKINS
MEDICINE

Cell Line Databases and Percent Match

- The GRCF carries out STR profiling following *ANSI/ATCC ASN-0002-2011, Authentication of Human Cell Lines: Standardization of STR Profiling* guidelines. The profile determined for your line can be used for comparison to the STR profile of the cell line from which it was originally derived in order to confirm authenticity or to establish an identity profile for a newly generated cell line. The GRCF uses the below databases for comparisons. Verification tools on the websites can be used to determine relatedness of your line to those held by the repositories. Search these sites by entering the profile of your line that was generated by GRCF.

  Leibniz Institute DSMZ German Collection of Microorganisms and Cell Cultures http://www.dsmz.de/services/services-human-and-animal-cell-lines/online-str-analysis.html

  American Tissue Culture Collection http://www.atcc.org/CulturesandProducts/CellBiology/STRProfileDatabase/tabid/174/Default.aspx

- The algorithms used in the ANSI/ATCC ASN-0002-2011 report, and by these repositories, calculate percent match or evaluation value (EV) differently. The GRCF reports percent match or EV as presented by the databases. Below are the algorithms. The ANSI/ATCC ASN-0002-2011 report gives the following guidelines for authentication: if ≥ 80% of the alleles in your STR profile match the profile of the donor, then the line can be considered related and derived from a common ancestor; between 55% to 80% match, they are most likely unrelated, but additional study may be needed to determine relatedness; and below 55% match the lines are unrelated.

  ANSI/ATCC ASN-0002-2011 states that, "The matching algorithm to determine percent % match between two cell lines equal = the number of shared alleles in both STR profiles divided ÷ by the total number of alleles in the questioned profile (homozygous alleles are counted as one allele)." The questioned profile is the line submitted for authentication.

  > Please note that the % match generated can very depending on the number of alleles genotyped in the questioned and reference samples, therefore, results should be interpreted carefully. The formula will provide a higher percent match if all of the alleles in the questioned sample match the reference sample alleles and the reference sample exhibits more alleles than the shared number of alleles.

  ATCC uses equation percent match = the number of shared alleles x 2 ÷ total alleles.

  DSMZ uses the algorithm: EV (Evaluation value) = (number of generated peaks cell line A) x 2 ÷ total number of peaks of cell line A + B
  DSMZ notes that EVs may be >1 because the number of alleles analyzed per cell line can vary.

  The International Cell Line Authentication Committee (ICLAC) http://iclac.org/ recommends using the algorithm: percent match = the number of shared alleles x 2 ÷ the total number of alleles in the test sample profile + the total alleles in the reference profile.

- If the profile of your cell line established by the GRCF is an exact match to that of a reference profile (profile of the tissue the cell line was originally derived), your line is considered related to the donor and authentic. If the profile is significantly different from a reference profile (<80%), the line is not authentic. Possibilities that could lead to a different profile are misidentification, mislabeling, or contamination from another cell line. If there are no matches to cell lines within the ATCC and DSMZ databases, your line is not misidentified as, or contaminated with any of the cell lines from these repositories. However, if your cell line is a novel line, a direct comparison to the donor is needed to determine if it is unique.

- STR analysis will not discriminate between monozygotic twins or cells derived from different tissues of the same donor or between sublines of the same parental cell line.

- The GRCF DNA Services strongly suggest you check that your line is not known to be a misidentified line. A database of misidentified lines curated by the ICLAC can be obtained at http://iclac.org/wp-content/uploads/Cross-Contaminations-v8_0.pdf (version 8.0, 1/12/2016). Capes-Davis A, Theodosopoulos G, Atkin I, Drexler HG, Kohara A, MacLeod RAF, Masters JR, Nakamura Y, Reid YA, Reddel RR, Freshney RI (2010). Check your cultures! A list of cross-contaminated or misidentified cell lines. Int J Cancer 127 (1): 1-8 [PMIPMID 20143388D 20143388].

- Many helpful resources for authentication of your cell line can be found on the International Cell Line Authentication Committee website http://iclac.org/.

Helpful Links

- Many helpful resources for authentication of your cell line can be found on the International Cell Line Authentication Committee website http://iclac.org/.
- Implementing Rigor and Transparency in NIH & AHRQ Research Grant Applications https://grants.nih.gov/grants/guide/notice-files/NOT-OD-16-011.html
- Journals with cell line authentication requirements https://www.scoop.it/t/cell-line-contamination/?tag=journals+asking+authentication%20%20

grcf at Johns Hopkins
DNA Services/FAF

Laura Kasch-Semenza
Johns Hopkins University
2760 Light House Point East, Suite 201
Baltimore, Maryland 21224
Phone: 410-614-3830
Email: lkasch@jhmi.edu

JOHNS HOPKINS MEDICINE

STR Profile Report for Cell Line Authentication

Principal Investigator: Tian Li Wang
Institution: Johns Hopkins University
Cell Line ID: SKOV3
Report Date: May 31, 2015
Submitting Researcher: Jin Jung
Sample Type Submitted: DNA
GeneSifter Tracking ID: 120058
Profile Kit Requested: GenePrint 10 (Promega)

STR Profile:

Loci	SKOV3
AMEL	X
CSF1PO	11
D13S317	8,11
D16S539	12
D21S11	30,31,31.2
D5S818	10,11
D7S820	13,14
TH01	9,9.3
TPOX	8,10,11
vWA	17,18

STR Graphic Profile:

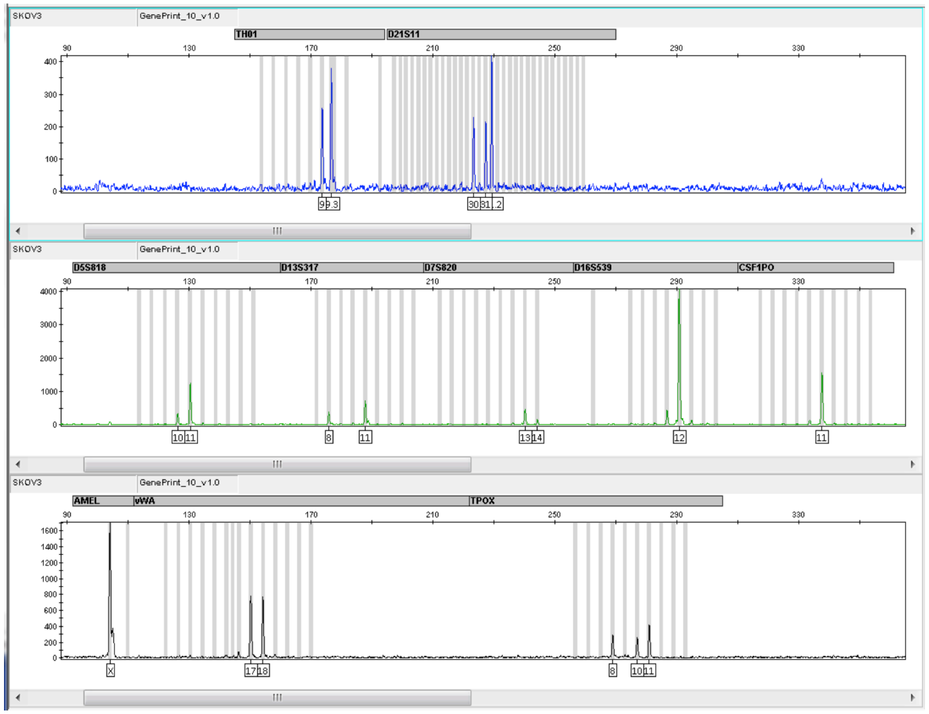

Peaks that appear to be legitimate stutter or are spectral bleed-through artifacts are not labeled.

e-Signature Director of Laboratory:

Laura Kasch
Digitally signed by Laura Kasch
DN: cn=Laura Kasch, o=Genetic Resources Core Facility, ou=Johns Hopkins University, email=lkasch@jhmi.edu, c=US
Date: 2015.05.31 20:20:12 -05'00'

FAF services are for research purposes only. Any data or samples generated by the facility may not be used for human diagnostic or therapeutic use. Results may not be returned to patients, placed into medical records, or be used to make clinical decisions.

grcf at Johns Hopkins
DNA Services/FAF

Laura Kasch-Semenza
Johns Hopkins University
2760 Light House Point East, Suite 201
Baltimore, Maryland 21224
Phone: 410-614-3830
Email: lkasch@jhmi.edu

JOHNS HOPKINS MEDICINE

Principal Investigator: Tian Li Wang
Institution: Johns Hopkins University
Cell Line ID: SKOV3
Report Date: May 31, 2015

Procedure:
A Promega GenePrint 10 Kit was used to polymerase chain (PCR) amplify eight short tandem repeat (STR) loci plus a gender determining marker, Amelogenin. The PCR product was electrophoresed on an ABI Prism® 3730xl Genetic Analyzer using an Internal Lane Standard 600 (Promega). Data was analyzed using GeneMapper® v 4.0 software (Applied Biosystems). Appropriate positive and negative controls were used.

- Please see the Pdf file *Additional Information* sent with the results for percent match calculations and additional information about interpreting the STR profile results.

Raw Data Table:

Sample Name	Marker	Allele 1	Allele 2	Allele 3	Size 1	Size 2	Size 3	Height 1	Height 2	Height 3	Peak Area 1	Peak Area 2	Peak Area 3
SKOV3	AMEL	X			103.99			1709			10820		
SKOV3	CSF1PO	11			337.58			1555			10569		
SKOV3	D13S317	8	11		175.81	187.74		381	729		2361	4505	
SKOV3	D16S539	12			290.84			4076			27739		
SKOV3	D21S11	30	31	31.2	223.28	227.27	229.28	227	214	419	1413	1285	2538
SKOV3	D5S818	10	11		126.25	130.45		349	1260		2077	7651	
SKOV3	D7S820	13	14		240.19	244.28		480	152		3065	1048	
SKOV3	TH01	9	9.3		173.7	176.63		257	379		1674	2354	
SKOV3	TPOX	8	10	11	268.99	277.04	280.94	291	261	417	2102	1767	2745
SKOV3	vWA	17	18		150.19	154.22		786	771		4909	4711	

FAF services are for research purposes only. Any data or samples generated by the facility may not be used for human diagnostic or therapeutic use. Results may not be returned to patients, placed into medical records, or be used to make clinical decisions.
