# Supplementary material for: Phosphodiesterase 7: a potential novel therapeutic target in ovarian cancer
Source: Front Pharmacol. 2025 Jun 4;16:1566330. doi: 10.3389/fphar.2025.1566330 (PMC12174393; doi:10.3389/fphar.2025.1566330)
Supplement: Supplementary file 8 [file DataSheet8.pdf]

S2 Table : Primers used in the gene expression analysis by RT-qPCR.

Gene	Primer Sequence (5' →3')
GAPDH	Forward:CAGCCTCAAGATCATCAGCA<br>Reverse:ACAGTCTTCTGGGTGGCAGT
ATAD2	Forward:TCCGGTGGTAGGTTTCAACT<br>Reverse:TTCCTCAGTCTGGAGCACATC
CCL2	Forward:AGGTGACTGGGGCATTGAT<br>Reverse:GCCTCCAGCATGAAAGTCTC
CCND2	Forward:ACGGTACTGCTGCAGGCTAT<br>Reverse:AGCTGCTGGCTAAGATCACC
CRELD2	Forward:AGTCCGGCACATTACAGGTC<br>Reverse:CTTCGAAGGAACGGAAGATG
CYP1B1	Forward:CTGCACTCGAGTCTGCACAT<br>Reverse:TATCACTGACATCTTCGGCG
MUC16	Forward:CTCTCAGCCTCCCAAGTGTC<br>Reverse:GTGTCCATGGTGGGGAATAC
STMN1	Forward:AGCTGCTTCAAGACCTCAGC<br>Reverse:ATCTGTTCCAGAATTCCCCC
VNN2	Forward:TTACCAAACGCCCATCTTTC<br>Reverse:GTTCTCCCTCAGTGGCACAT
IL1-α	Forward:CATCCTCCACAATAGCAGACAG<br>Reverse:GAGTTTCCTGGCTATGGGATAG
IL1-β	Forward:CAAAGGCGGCCAGGATATAA<br>Reverse:CTAGGGATTGAGTCCACATTCAG
IL-6	Forward:CATTTGTGGTTGGGTCAGG<br>Reverse:AGTGAGGAACAAGCCAGAGC
GP130	Forward:TGCCTCCAGAAAAACCTAAAAA<br>Reverse:TTTGTCTCCAAGTGTGTTTCC
NANOG	Forward:GCCCTGCACCGTCACCC<br>Reverse:ACCAGGTCTTCACCTGTTTGT
OCT4	Forward:GCCCTGCACCGTCACCC<br>Reverse:GCTTGGCAAATTGCATCGAGT
PDE7-A	Forward:TCCTTGCAGAGACAGACACTTC<br>Reverse:CCGAGCAGGATTTGAATCAG
